# Mechanistic Insights into the Regio‐ and Stereoselectivities of Testosterone and Dihydrotestosterone Hydroxylation Catalyzed by CYP3A4 and CYP19A1

**DOI:** 10.1002/chem.201905272

**Published:** 2020-04-28

**Authors:** Junhao Li, Yun Tang, Weihua Li, Yaoquan Tu

**Affiliations:** ^1^ Department of Theoretical Chemistry and Biology KTH Royal Institute of Technology Roslagstullsbacken 15 10691 Stockholm Sweden; ^2^ Shanghai Key Laboratory of New Drug Design East China University of Science and Technology Meilong Road 130 200237 Shanghai P.R. China

**Keywords:** C−H activation, density functional calculations, hydroxylation, molecular modeling, P450, steroids

## Abstract

The hydroxylation of nonreactive C−H bonds can be easily catalyzed by a variety of metalloenzymes, especially cytochrome P450s (P450s). The mechanism of P450 mediated hydroxylation has been intensively studied, both experimentally and theoretically. However, understanding the regio‐ and stereoselectivities of substrates hydroxylated by P450s remains a great challenge. Herein, we use a multi‐scale modeling approach to investigate the selectivity of testosterone (TES) and dihydrotestosterone (DHT) hydroxylation catalyzed by two important P450s, CYP3A4 and CYP19A1. For CYP3A4, two distinct binding modes for TES/DHT were predicted by dockings and molecular dynamics simulations, in which the experimentally identified sites of metabolism of TES/DHT can access to the catalytic center. The regio‐ and stereoselectivities of TES/DHT hydroxylation were further evaluated by quantum mechanical and ONIOM calculations. For CYP19A1, we found that sites 1β, 2β and 19 can access the catalytic center, with the intrinsic reactivity 2β>1β>19. However, our ONIOM calculations indicate that the hydroxylation is favored at site 19 for both TES and DHT, which is consistent with the experiments and reflects the importance of the catalytic environment in determining the selectivity. Our study unravels the mechanism underlying the selectivity of TES/DHT hydroxylation mediated by CYP3A4 and CYP19A1 and is helpful for understanding the selectivity of other substrates that are hydroxylated by P450s.

## Graphical abstract

**Selective insertion**: The results of a theoretical study on the selective hydroxylation of testosterone and dihydrotestosterone by CYP3A4 and CYP19A1 is reported (see scheme).

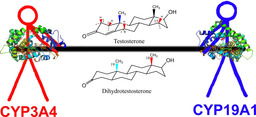

## Introduction

The human cytochrome P450 superfamily includes 57 isoforms of heme‐enclosed enzymes that catalyze the redox reactions of a variety of endogenous and exogenous compounds.^1^ Cytochromes P450s (P450s) are classified as monooxygenases because in most reactions an oxygen atom is inserted into the C−H bond and the substrate is thereby hydroxylated.^2^ The molecular mechanism of this reaction has been a subject of intense studies both experimentally and theoretically^3^ and is currently recognized as a hydrogen atom transfer (HAT) followed by a radical “rebound” step.2a, 4 However, details of the mechanism leading to regio‐ and stereoselective P450 mediated hydroxylation are still not clear.4d, 5

In the human body, hydroxylation reactions catalyzed by P450s are important in phase I metabolism.1b Studying the mechanisms of the regio‐ and stereoselectivities of the hydroxylation reactions is beneficial for understanding the occurrence of reactive phase I metabolites and for finding ways to avoid the occurrence.1b For instance, by studying the regioselectivity of tienilic acid hydroxylation, which occurs at site 5, the mechanism for the occurrence of the reactive metabolites was disclosed.^6^ Many experimental and theoretical studies have been carried out for predicting the selectivity of P450 catalyzed hydroxylation reactions.^7^ Given the complexities of the P450 pocket and catalytic cycle and the diversity of substrates, understanding the selectivity of P450 hydroxylation remains a great challenge.3c, 7

As an important case,3c, 8 the mechanism of selective hydroxylation reactions of steroid substrates catalyzed by two P450 isoforms, CYP3A4 and CYP19A1, is still unclear. CYP3A4 is the most abundant P450 and is responsible for the metabolism of about 50 % of clinically used drugs.1b, 8a It also plays important roles in the regulation of endogenous steroids, the main component of hormones.^9^ CYP19A1, also known as steroid aromatase, mainly converts androgens into estrogens and is therefore involved in the pathological progress of estrogen‐dependent diseases, such as breast and ovary cancers.^10^ Downregulation of CYP19A1’s activity has been deemed as a promising strategy for treating such diseases.^11^

**Figure 1:**
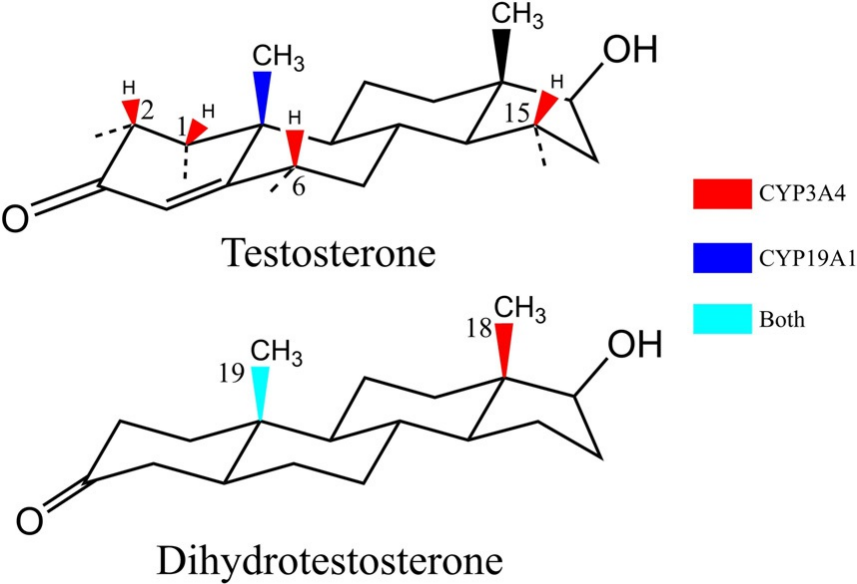
Chemical structures of TES and DHT and the corresponding SOMs. The bonds connecting the α hydrogen atoms are represented as dashed lines. The SOMs of TES/DHT by CYP3A4 and CYP19A1 are colored in red and blue, respectively; the SOM that can be hydroxylated by both CYP3A4 and CYP19A1 is colored in cyan.

In this work, we focus on the hydroxylation of two important steroid molecules, testosterone (TES) and dihydrotestosterone (DHT), catalyzed by CYP3A4 and CYP19A1. TES is a necessary male sex hormone and anabolic steroid.^12^ In a natural biotransformation process, TES is transformed either into DHT by the 5α‐steroid reductase or into estradiol by the aromatase.^13^ DHT is an androgen and is more potent than TES because it can directly activate the androgen receptor,^14^ although DHT and TES share similar chemical structures and conformations. In experiments, TES and DHT are hydroxylated by CYP3A4 at different sites. TES is hydroxylated at sites 6β, 2β, 15β, and 1β,^15^ whereas DHT is hydroxylated only at sites 18 and 19.^16^ In contrast, both TES and DHT are hydroxylated at site 19 by CYP19A1 (Figure 1).^16^ To date, the mechanism of selective hydroxylation of TES/DHT by CYP3A4 and CYP19A1 has remained elusive.3c

Here, we used a multi‐scale modeling approach, which involves molecular dockings, molecular dynamics (MD) simulations, quantum mechanics (QM) calculations, and quantum mechanics/molecular mechanics (QM/MM) calculations, to investigate the mechanism underlying the selective hydroxylation of TES and DHT by CYP3A4 and CYP19A1. The accessibility of each potential site of metabolism (SOM) to an enzyme's reaction center was evaluated by 100‐ns long MD simulations. The intrinsic reactivities of the potential SOMs were explored by using a prevailing truncated heme model. Together with the QM/MM calculations, our results unravel the mechanism underlying the selectivity of hydroxylation of TES and DHT by CYP3A4/CYP19A1.

## Results and Discussion

### Binding modes of TES

There are several sites of TES that could potentially be oxidized by P450s. The experimentally determined oxidation sites of TES by CYP3A4 are 6β, 2β, 15β and 1β, and the corresponding reaction rates in the recombinant CYP3A4 enzyme are 83, 11, 5.0, and 4.8 min^−1^, respectively.15b By contrast, the experimentally determined oxidation site of TES by CYP19A1 is the 19 site.^16^

Since the crystal structure of the CYP3A4‐TES complex is not available, molecular docking was used to predict the initial binding mode of TES with CYP3A4. The experimental SOMs of TES are distributed in different rings, which means that multiple binding modes of TES could exist in the CYP3A4 active site. To consider the effect of active site residues on the binding of TES, multiple CYP3A4 crystal structures were used for docking.

**Figure 2:**
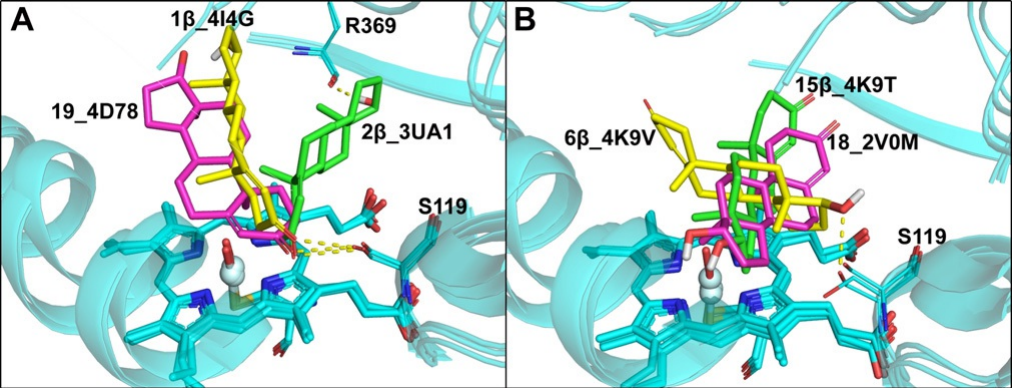
Binding modes predicted by molecular docking. A) The three representative 17‐OHUP binding modes: 194D78 (magenta), 1β4I4G (yellow) and 2β3UA1 (green); B) The three representative 17‐OHDOWN binding modes: 6β4K9V (yellow), 15β4Κ9Τ (green) and 182V0M (magenta).

We have analyzed the root mean square fluctuation (RMSF) for each residue averaged over the 11 CYP3A4 crystal structures (Figure S1). The RMSFs for about 80 % of the residues are below 1.0 Å and those for the active site residues are mostly above 1.0 Å, especially for the residues of the F‐F′ helix, which show high RMSFs (ca. 4–5 Å). We therefore believe that the difference in the selected crystal structures is large enough for generating reasonable binding modes for TES to CYP3A4. By using these structures as the docking receptors, all experimental SOMs and the angular methyl groups were found to expose to the oxo moiety in the top ranked poses, named 6β‐4K9V, 2β‐3UA1, 1β‐4I4G, 15β‐4K9T, 18‐2V0M, and 19‐4D78. As shown in Figure 2, two distinct binding modes of TES, 17‐OH‐UP and 17‐OH‐DOWN, were identified. In the 17‐OH‐UP binding mode, the 17‐OH group points to the upper hall of the active sites, as shown in 2β‐3UA1, 1β‐4I4G and 19‐4D78. The 3‐ketone group forms a hydrogen bond with Ser119, which is a key residue for the interactions between CYP3A4 and its substrates.^17^ In addition, the 17‐OH group in the 2β‐3UA1 mode forms a hydrogen bond with the backbone atoms of Arg369 (Figure 2 A). In the 17‐OH‐DOWN binding mode, 17‐OH points to the B‐C loop, as shown in 6β‐4K9V, 15β‐4K9T and 18‐2V0M. The hydrogen bond between 17‐OH and Ser119 was only observed in the 6β‐4K9V mode.

### Accessibility profiles of TES and DHT

MD simulations have been widely used in the study of P450 enzymes for a broad range of interests, including flexible conformational sampling,^18^ ligand binding free energy calculation,^19^ conformational transitions,^20^ allosteric regulation,^21^ and substrate accessibility evaluation.^22^ The accessibility profile obtained from the equilibrated MD simulations (Figure S2) can be used to describe the extent of a site exposure to the oxo moiety of Cpd I and can provide useful information for understanding P450 catalytic selectivity.^22^, ^23^ The evaluation of the accessibility profile was based on the distance between the SOM hydrogen atom and the oxo atom of Cpd I (denoted as “H‐oxo”) as well as the angle formed by the SOM hydrogen, oxo, and iron atoms (denoted as “H‐oxo‐FE”).^22^ Based on the geometry from the typical HAT species for C−H hydroxylation,2a the conformation for an accessible site, which is also referred to as the near attack conformation,^24^ should satisfy the criterion of the H‐oxo distance in the range of 2.0–3.5 Å and the H‐oxo‐FE angle above 120°.

**Figure 3:**
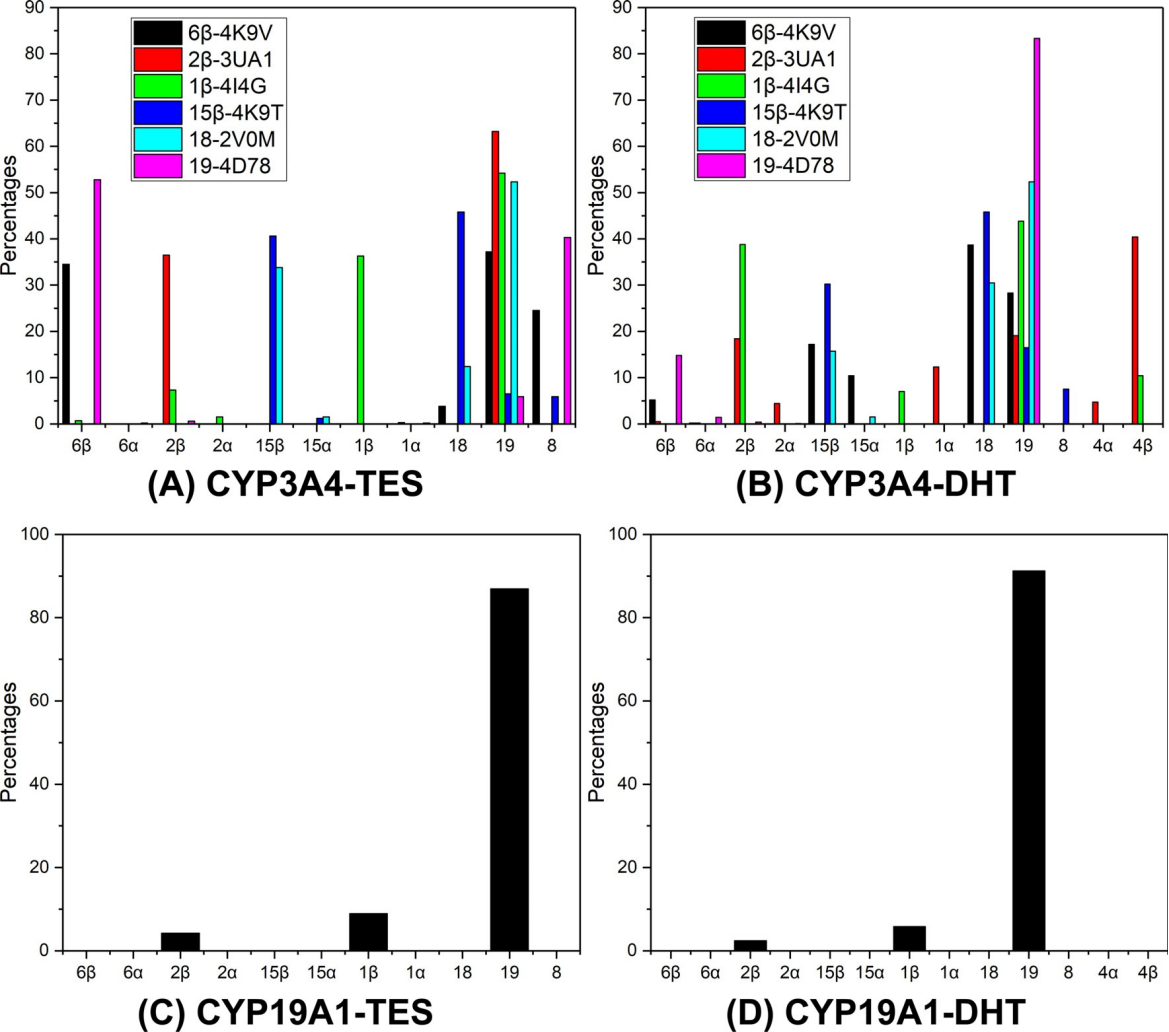
Accessibility profiles from the MD simulations. The percentage for a site is the number of snapshots in which the site was accessed divided by the number of snapshots for all the accessed sites.

By applying the criterion, the accessibility for each site in the 12 CYP3A4 systems (corresponding to six docked TES and six superimposed DHT binding modes) and two CYP19A1 systems was evaluated as summarized in Figure 3. All CYP3A4 systems exhibited multiple accessible sites (the DHT‐2β‐3UA1 system has the largest number of sites, in which sites 2β, 2α, 1α, 19, 4α and 4β are accessible, see the red columns in Figure 3 Β), indicating that the binding modes of TES/DHT underwent changes frequently during the 100‐ns MD simulations. This is not surprising because the size of the CYP3A4 binding pocket is relatively large, which allows different sites of TES/DHT to access the oxo moiety. In some systems (TES‐1β‐4I4G, DHT‐6β‐4K9V, DHT‐2β‐3UA1, DHT‐18‐2V0M and DHT‐19‐4D78, Figure 3 A and B), even the steroid scaffold could flip, leading to a small proportion (less than 5 %) of α sites exposed to the Cpd I oxo moiety.

For the 19‐4D78 system, the 6β site of TES is the dominant accessible site (ca. 54 %, magenta column in Figure 3 A), reflecting the strong preference for 6β hydroxylation in 4D78, which explains the experimental results. Nevertheless, for the 6β‐4K9V, 2β‐3UA1 and 1β‐4I4G systems, site 19 has the highest accessibility, followed by the respective 6β, 2β and 1β sites (see the black, red, and green columns in Figure 3 A). It seems that there exists competition between site 19 and sites 6β, 2β, and 1β for accessing the oxo moiety, since the three hydrogen atoms of site 19 are very close to sites 6β, 2β and 1β, respectively (Figure S3). These results also reflect the intricacy in predicting the TES binding modes in the large and flexible active site of CYP3A4. The complexity has also been observed experimentally, which indicated that CYP3A4 can bind up to three TES molecules and alter the binding kinetics.^25^

Similarly, it becomes more complicated to predict the binding modes of DHT in the CYP3A4 binding pocket. For the 2β‐3UA1 system, the additional 4β and 4α sites in ring A were also the accessible sites (see in Figure 3 B). For the 6β‐4K9V and 2β‐3UA1 systems, the most accessible sites are 18 and 4β, respectively. Similar to the CYP3A4‐TES system, site 18 was more optimal to access to the oxo moiety in the 15β‐4K9T systems (see the blue columns in Figure 3 A and B). As we can see, not all systems show the results matching the experimental selective profile of DHT hydroxylation by CYP3A4 (e.g., the 2β‐3UA1 system). Nevertheless, sites 18 and 19 were still the dominating accessible sites in all the MD simulations of CYP3A4‐DHT (Figure 3 B). Thus, the accessibility profile provides an explanation of why CYP3A4 mainly hydroxylates DHT at sites 18 and 19, instead of the reactive β sites.

In the MD simulations of the CYP19A1‐TES/DHT systems, the frequencies of shifting accessible sites are much lower than those in the CYP3A4 systems, because more than 85 % of the observed accessible sites are located at site 19 (Figure 3 C and D). The 6β site did not access the active site, which also means that site 19 is much more competitive than site 6β in accessing the oxo moiety (Figure S2). Unlike in CYP3A4, replacement of TES with DHT hardly changed the binding mode in CYP19A1, because the hydrogen bonds between TES/DHT and Asp309 and Met374 of CYP19A1 (see E–F in Figure S4) are more stable than that between TES/DHT and Ser119 of CYP3A4 (see A–D in Figure S4). Thus, our analysis of the accessibility profiles explains why the more reactive 6β or 2β site is not hydroxylated in CYP19A1 and agrees with the aromatic mechanism of CYP19A1.^26^

After inspection of the accessibility profiles, we conclude that the potential SOMs of TES/DHT are in competition for being exposed to the reaction center. The regio‐ and stereoselectivities of TES/DHT hydroxylation reactions could be explained by the accessibility profiles. However, MD simulations only reflected the “competition” between certain sites. The mechanisms underlying such site “competition” will be further clarified by the subsequent QM and QM/MM calculations.

### Activation barriers

#### Intrinsic reactivity of potential SOMs

The HAT process for the P450 catalyzed C−H hydroxylation is a rate‐limiting step and the corresponding activation barrier governs the reaction.2a Both the intrinsic reactivity of the substrate and the environment of the enzyme active site can affect the activation barrier that dominates the selectivity of hydroxylation.^7^ Here, we firstly investigate the intrinsic reactivity of the potential SOMs of TES to understand its role in the selectivity of TES hydroxylation. Since DHT has a similar scaffold to TES, we believe that the intrinsic reactivity of a DHT site could be deduced from that of the corresponding TES site.

The activation barriers for the 6β, 6α, 2β, 2α, 15β, 15α, 1β, 1α, 8, 18 and 19 sites were calculated and are presented in Table 1. The activation barriers for the 6β, 2β, 15β and 1β sites are 5.3, 9.9, 10.2 and 13.5 kcal mol^−1^, respectively, which are in good agreement with the experimental data.15b For the four β sites, the activation barriers are also very close to those calculated in the previous study when the dispersion interaction is not considered.^27^ The high reactivity of the 6β and 2β sites do not come as a surprise because of the electron‐delocalization effect of the conjugated carbonyl moiety, which significantly stabilizes the TS structures. Both 2β and 6β are next to the conjugated carbonyl moiety, but the 2β site is closer to the ketone group, which makes it less reactive than the 6β site. The activation barriers for all the α sites are higher than the corresponding β sites. Especially, the activation barrier for the 6α site is ca. 7 kcal mol^−1^ higher than that of the 6β site. Compared with the TS structures for the α sites, the TS structures for the β sites can be stabilized by the interactions between the angular methyl group and Cpd I, which lowered the activation barriers for the β sites. For sites 8, 18 and 19, the activation barriers are higher than those for the α and β sites when the dispersion correction was not considered.

**Table 1:** Activation barriers for the potential SOMs of TES [kcal mol^−1^].

Site	DFT^[a]^	D3‐DFT^[b]^
1α	18.7	14.4
1β	19.1	13.5
2α	15.4	13.4
2β	13.0	9.9
6α	18.1	12.2
6β	10.3	5.3
15α	17.9	14.9
15β	15.5	10.2
18	20.4	13.0
19	22.8	18.3
8	21.1	10.0

[a] The activation barrier is the energy difference between the reactant complex (RC) and transition state (TS) calculated using the B3LYP functional with the BS2 basis set. The energies were corrected with ZPE using the BS1 basis set. [b] The activation barriers were calculated using the B3LYP‐D3 functional and Becke–Johnson damping with the BS2 basis set. The energies were also corrected with ZPE using the BS1 basis set.

Since the C−H bond‐dissociation energy (BDE) contributes significantly to the intrinsic reactivity of hydroxylation,^28^ the BDEs of all the C−H bonds of TES were calculated (Table S1).The BDEs for sites 6, 2, 15 and 1 show the same trend as in the work by other research groups^27^, ^29^ and are in accordance with the experimental distribution of the TES hydroxylation sites.15b However, due to the planar shape of the carbon radical, we are unable to use BDE to predict the stereoselectivity, that is, α or β hydroxylation, in the TES hydroxylation.

#### Activation barriers predicted by ONIOM calculations

Even though it has been revealed by the QM calculations that sites 18 and 19 of TES are much less reactive, these sites can still be preferentially hydroxylated by different isoforms of P450s. The MD simulation results have been used to explain the selectivity of the above hydroxylation reactions. To further understand the mechanism underlying the selectivity of TES/DHT hydroxylation reactions catalyzed by CYP3A4 and CYP19A1, ONIOM calculations were performed. For CYP3A4, we chose the snapshots from the MD simulations of the 19‐4D78 and 6β‐4K9V systems to represent the binding modes of 17‐OH‐UP and 17‐OH‐DOWN, respectively. For CYP19A1, the representative snapshots from the MD simulations were used for the ONIOM calculations.

### The 19‐4D78‐TES/DHT system

**Figure 4:**
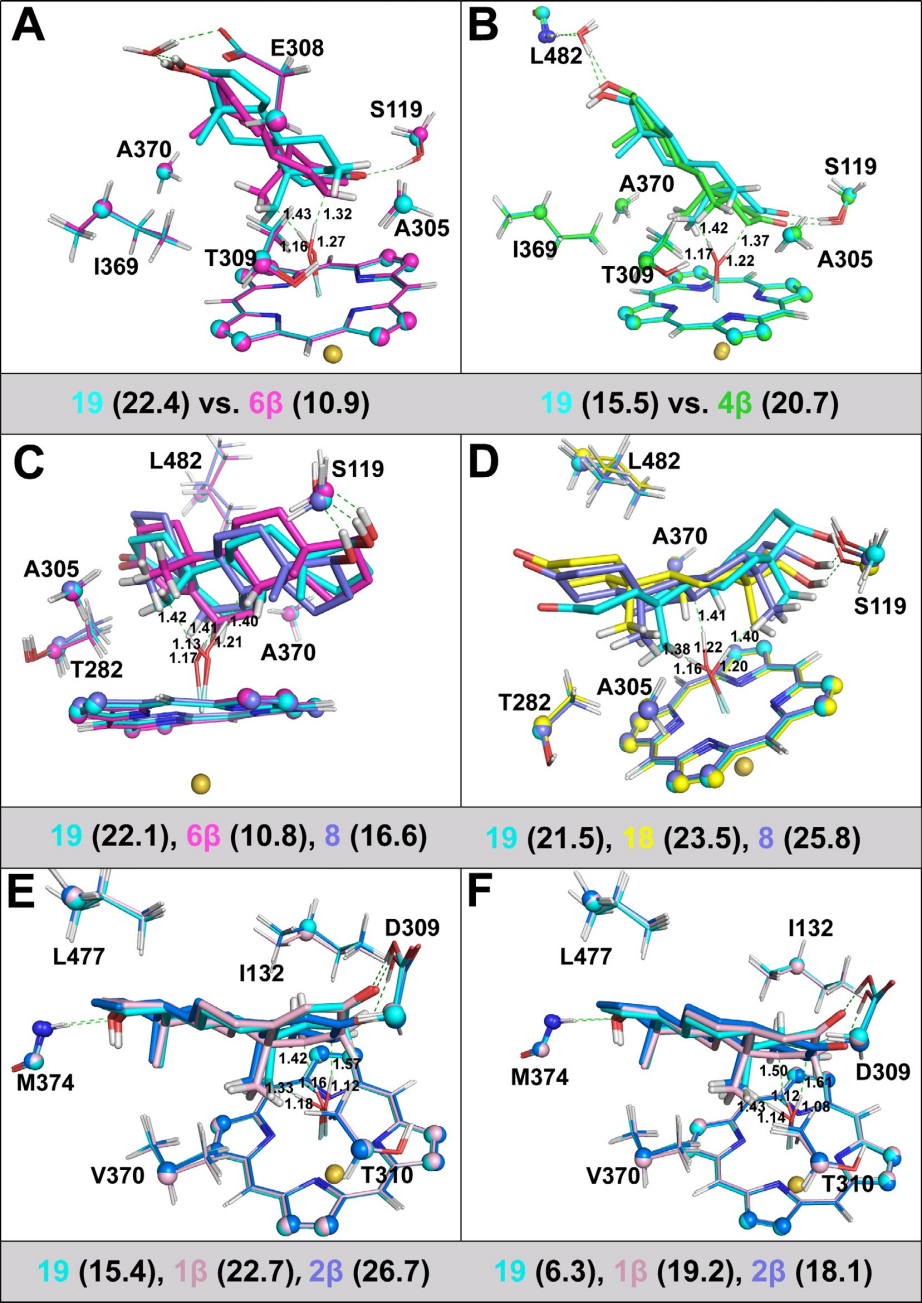
A) TS structures for sites 19 (in cyan) and 6β (in magenta) of TES in the 194D78 system. B) The TS structures for sites 19 (in cyan) and 4β (in green) of DHT in the 194D78 system. C) The TS structures for sites 19 (in cyan), 6β (in magenta) and 8 (in blue) of TES in the 6β4K9V system. D) The TS structures for sites 19 (in cyan), 18 (in yellow), and 8 (in blue) of DHT in the 6β4K9V system. E) The TS structures for sites 19 (in cyan), 1β (in light pink) and 2β (in blue) of TES in the CYP19A1 system. F) The TS structures for sites 19 (in cyan), 1β (in light pink) and 2β (in blue) of DHT in the CYP19A1 system. The activation barrier (in kcal mol^−1^) for each site is shown in parentheses. The H_abstracted_−oxo and H_abstracted_−C_site_ distances are in Å.

In MD simulations, the 17‐OH group of TES/DHT was close to the upper region of the active site and was able to interact with the side chain of Glu308 (Figure 4 A) or the backbone of Leu482 (Figure 4 B) via a water bridge. Meanwhile, the hydrogen bond between the 3‐ketone group and the side chain of Ser119 remains stable during the MD simulations. This binding mode favors sites 19 and 4β of DHT or sites 19 and 6β of TES to approach the oxo moiety. PES scanning indicated that the hydroxylation at site 6β of TES and site 19 of DHT is more favorable than at other sites. Significantly, the activation barriers for sites 6β of TES (10.9 kcal mol^−1^, Figure 4 A) and 19 of DHT (15.5 kcal mol^−1^, Figure 4 B) are lower than those for the other sites, which are in good agreement with the experimental observations.^16^

It should be noted that the 6β site of TES in the 17‐OH‐UP mode has a higher HAT barrier than that from the QM calculation (10.9 vs. 5.3 kcal mol^−1^, Figure 4 A and Table 1). Although the orientations of TES in the reactant complex (RC) and transition state (TS) for site 6β from the ONIOM calculations are similar to those from the QM calculations, it is likely that the smaller “H‐oxo‐FE” angle and more overlap between ring A of TES and the heme plane (Figure S5A) contribute to the lower barrier of 6β in the QM model. The 4β site of DHT is in the α‐position of the 3‐ketone group, which could have similar reactivity to the 2β site. However, for the 4β site, the hydrogen bond between the 3‐ketone group and Ser119 provides a steric hindrance, which raises the barrier. Although the binding modes of TES/DHT in the CYP3A4 active site are very similar (Figure S5B), there still exists rather significant difference between TES and DHT in “H‐oxo” and “H‐oxo‐FE” for the same site, which contributes to the change of the hydroxylation selectivity.

### The 6β‐4Κ9V‐TES/DHT system

In the 17‐OH‐DOWN mode, TES/DHT is parallel to the heme plane. The activation barriers for sites 19, 6β and 8 of TES are 22.1, 10.8 and 16.6 kcal mol^−1^ (Figure 4 C), respectively. Notably, the activation barrier for the 6β site of TES in this system is almost the same as in the 19‐4D78 system (10.9 kcal mol^−1^), suggesting that the 6β site of TES is rather reactive and its activation barrier is hardly affected by the binding modes. The high reactivity of the 6β site has been predicted in the QM model and is considered as the main driving force for the selective hydroxylation of TES by CYP3A4.

**Figure 5:**
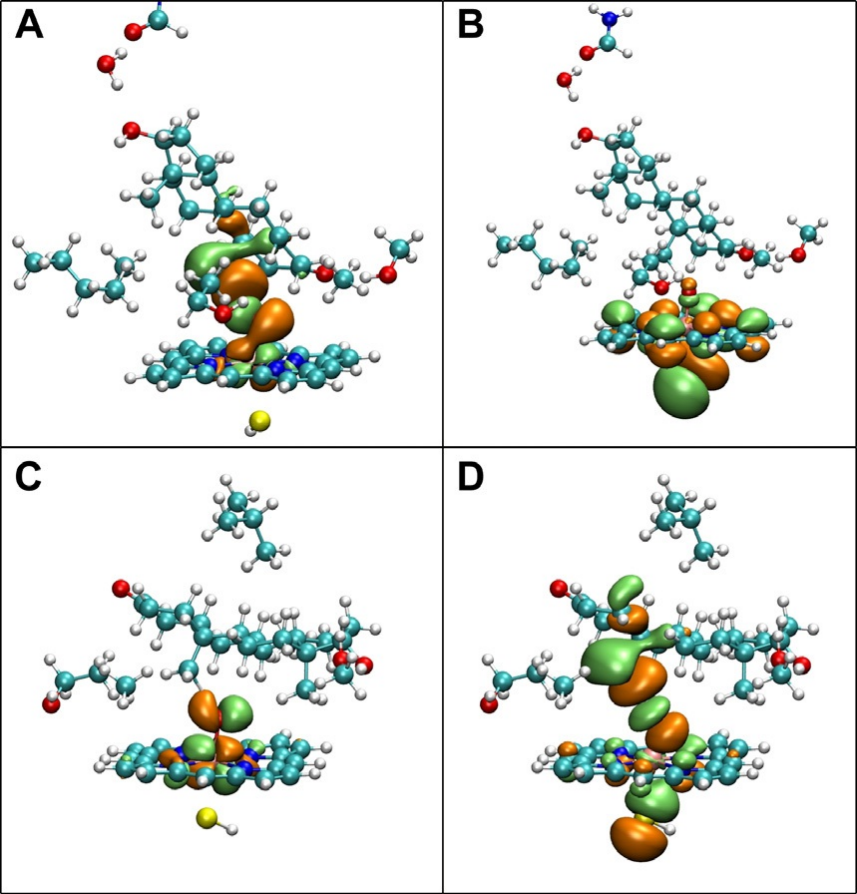
Spin natural orbital (SNO) distributions for the TS structures for site 19 in the CYP3A4‐DHT systems: A) the α electron density in 17‐OHUP; B) the β electron density in 17‐OHUP; C) the α electron density in 17‐OHDOWN; D) the β electron density in 17‐OHDOWN. The SNOs were generated by the Multiwfn 3.6 package^30^ and visualized using VMD 1.9.3^31^ with isovalue=0.02.

Similar to the 19‐4D78‐DHT system, the 6β site of DHT could not access the oxo moiety either, since the “H‐oxo‐iron” angle was almost linear and unfavorable for shifting electrons to the substrate. For sites 19, 18 and 8, the activation barriers were 21.5, 23.5 and 25.8 kcal mol^−1^ (Figure 4 D), respectively. The results from 19‐4D78 and 6β‐4K9V demonstrate that the preference of DHT hydroxylation is at the angular methyl group, although the activation barriers can be different in the two systems (15.5 vs. 21.5 kcal mol^−1^ in 19‐4D78 and 6β‐4K9V for site 19 of DHT). By using the spin natural orbital (SNO) analysis,^32^ we could further explain the significant difference in the activation barrier for site 19 in the 19‐4D78 and 6β‐4K9V DHT systems. As depicted in Figure 5, the electron distribution contributing to the HAT process can be differentiated for the two systems. In the 19‐4D78 system the HAT process was dominated by the α electrons (Figure 5 A), whereas in the 6β‐4K9V system it was dominated by the β electrons (Figure 5 D). The SNO distributions indicate that the 17‐OH‐UP binding mode favors the shift of the α electrons to the substrate and lowers the C−H activation barrier for site 19 of DHT.

### The CYP19A1‐TES/DHT system

The hydroxylation of site 19 is the first step in CYP19A1 mediated TES aromatization, which finally produces estrogens.10a The mechanism of the last step in aromatization is still controversial and has attracted much attention.^33^ However, less attention has been paid to the question of why site 19 is easier for hydroxylation than the more reactive adjacent sites 2β and 1β in the first step. Interestingly, upon replacing TES with DHT, the hydroxylation sites unexpectedly switch to sites 18 and 19 for CYP3A4, while for CYP19A1, the hydroxylation still occurs at site 19.

From the QM calculations, we learned that the 6β site of TES is most reactive. However, this site is far away from the Fe atom and even beyond the “6 Å” rule in the crystal structure of CYP19A1‐TES,^34^ which shows a binding mode similar to that in 17‐OH‐DOWN where the steroid scaffold is parallel to the heme plane. In the active site of CYP19A1, the scaffold of TES is perpendicular to helix I and stabilized by the two hydrogen bonds formed by TES with the two distant residues in the edges of the active site, which are the protonated side chain of Glu308 in helix I and backbone of Met374 in the β3 sheet (Figure S6). The PES scanning was able to locate the TSs for sites 19, 2β and 1β of TES. Of the three sites, site 19 has the lowest activation barrier (15.4 kcal mol^−1^, Figure 4 E), showing that hydroxylation at site 19 is most favorable. Cheng et al. observed that CYP19A1 is about 10^3^‐fold more efficient than CYP3A4 in generating the 19‐hydroxylated DHT.^16^ As we can see from Figure 4, the activation barriers correlate with the experimental observations.

**Figure 6:**
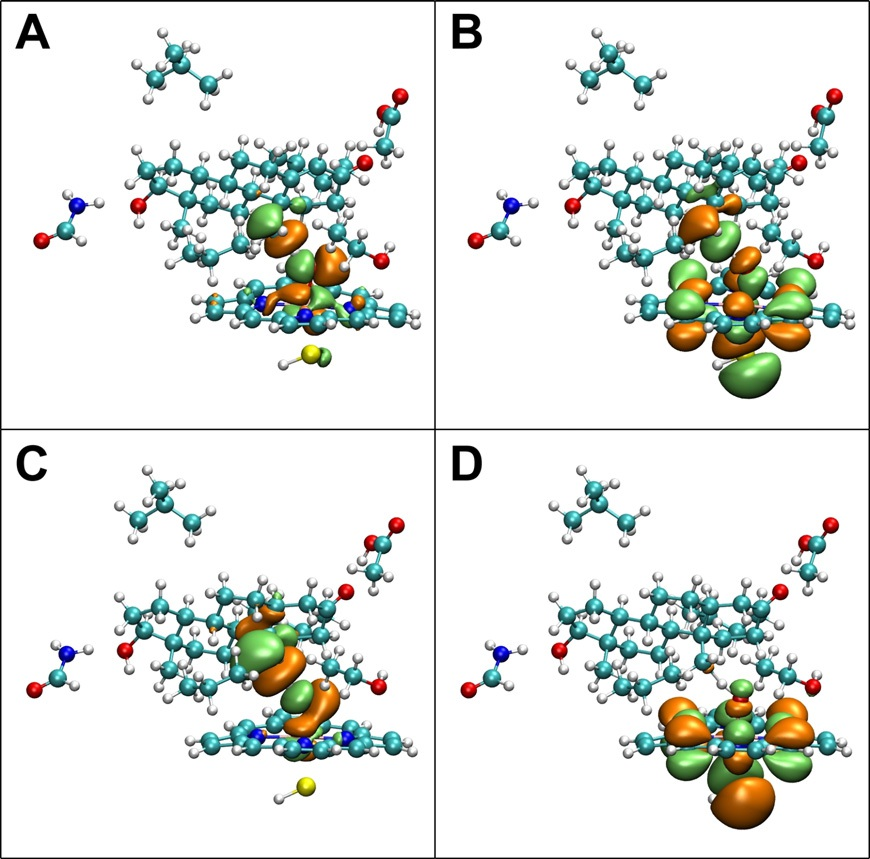
Spin natural orbital (SNO) distributions for the TS structures for site 19 in the CYP19A1 systems: A) the α electron density in CYP19A1‐TES; B) the β electron density in CYP19A1‐TES; C) the α electron density in CYP19A1‐DHT; D) the β electron density in CYP19A1‐DHT. The SNOs were generated by the Multiwfn 3.6 package^30^ and visualized using VMD 1.9.3^31^ with isovalue=0.02.

For each site in DHT, the corresponding activation barrier is lower than that in TES (Figures 4 E and F). Replacing TES with DHT slightly shortened the “H‐oxo” distance in the TSs, which could be one of the reasons for the lower activation barriers for DHT. Additionally, the SNO analysis of the TS for site 19 of TES indicates that the α and β electrons contribute almost equally to the HAT step (Figure 6 A and B), whereas for site 19 of DHT, the HAT step is mainly determined by the behavior of the α electrons (Figure 6 C and D). Referring to our previous SNO analysis of the 17‐OH‐DOWN system, we believe that a modest difference in the P450 active site configuration could significantly affect the site‐preference of the HAT step.

## Conclusion

CYP3A4 and CYP19A1 exhibited different regio‐ and stereoselectivities of hydroxylation towards TES/DHT. In this work, we examined these reactions in depth by taking into account the ligand reactivity and enzyme environment.

By using the docking approach, we found that there exist two binding modes of CYP3A4‐TES; namely, 17‐OH‐UP and 17‐OH‐DOWN. MD simulations verified the stability of these binding modes. Furthermore, 6β was the most accessible site in the TES‐19‐4D78 system, with the accessibilities of sites 18 and 19 being significantly higher than other sites, especially in the DHT systems. From the QM calculations, we found that all the β sites are more reactive than the corresponding α sites, which explains the stereo‐selectivity of TES hydroxylation by CYP3A4. The results from the ONIOM calculations indicate that the hydroxylation of TES at the 6β site is independent of the binding mode, which explains why the 6β site is a major SOM. In the CYP3A4‐DHT systems, site 19 is more reactive in the 17‐OH‐UP binding mode than in 17‐OH‐DOWN because the activation barrier for site 19 is lower in 17‐OH‐UP.

Although the crystal structure of CYP19A1‐TES has provided some clues for understanding the selectivity of hydroxylation, the structure still cannot be used to explain why the more reactive juxtaposition sites of TES/DHT are not the first choice of hydroxylation.^35^ Our MD simulations and ONIOM calculations indicate that site 19 of DHT is easier to be hydroxylated by CYP19A1 than that of TES.

In conclusion, our study has revealed the mechanism of the TES/DHT hydroxylation selectivity mediated by CYP3A4 and CYP19A1. Our results are also useful for understanding the regio‐ and stereoselectivities for the hydroxylation of other steroid molecules catalyzed by P450s.

## Experimental Section

### Molecular docking

The initial model of CYP3A4 in complex with TES was obtained by molecular docking, which was executed by using GOLD Suite 5.2.^36^ There are more than 30 crystal structures of CYP3A4 available at present. Based on the shape of the active sites, 11 crystal structures were selected for molecular docking and the root mean square fluctuation for each residue averaged over these structures was analyzed. The PDB codes of these structures are 1TQN,^37^ 2V0M,^38^ 3NXU,^38^ 3UA1,^39^ 4D78,^40^ 4I4G,^41^ 4K9T,^42^ 4K9V,^42^ 4K9W,^42^ 5TE817b and 5VC0.^43^ They were prepared using the protein preparation wizard in Schrödinger suite 2016‐1.^44^ The coordinates of missing residues were added by Prime^45^ and the protonation states of ionizable residues were assigned by Protassigner,^46^ while all co‐crystalized ligands and water molecules were removed. The Cpd I form of heme was manually prepared in Maestro 10.5.^47^

The docking center was set on the Cpd I oxo atom and the space within 15 Å around the center was defined as the docking site. Fifty docked poses were generated for each docking and ranked by ChemScore with the scoring template parameterized for heme‐proteins.^48^ Because DHT is highly similar to TES in both chemical structure and conformation, the initial CYP3A4‐DHT complexes were obtained by superimposing DHT onto each of the top‐ranked TES poses. The initial structure of the CYP19A1‐TES complex was taken from the crystal structure (PDB code: 5JKW).^49^ Similarly, DHT was superimposed to TES to obtain the CYP19A1‐DHT complex.

### MD simulations

All the complexes obtained from the above step, that is, 12 CYP3A4‐TES/DHT and 1 CYP19A1‐TES/DHT, were subjected to 100‐ns MD simulations. The *pmemd.cuda* module^50^ of Amber16^51^ was used for the MD simulations with random seeds for the initial velocities. The Amber14SB^52^ and general Amber force field (GAFF)^53^ were applied for the protein and the ligands, respectively. The crystallographic water molecules were recovered for the docked/superimposed CYP3A4‐TES/DHT complexes, with the protonation states of ionizable residues remaining the same as in the docking procedure. Similarly, PROPKA 3.1 in Schrödinger Suite was employed to determine the ionizable residues’ protonation states in CYP19A1.^54^ The TES and DHT geometries were optimized by using Gaussian 09 at the B3LYP/6‐31G* level, followed by using the standard restrained electrostatic potential (RESP) fitting procedure to derive the atomic charges.^55^ The truncated octahedron TIP3P water box and counterions were used to solvate and neutralize each system, respectively.^56^ The force field parameters for the Cpd I moiety were adopted from Shahrokh's work.^57^

For each system, the energy optimization was carried out using the gradually decreased force constant restraints on the heavy atoms of the protein and ligand. The temperature of the system was then gradually raised from 0 K to 300 K in 80 ps using the NVT ensemble with the restraints the same as in energy minimization. An 80‐ps equilibration at 300 K without constraints on atoms was carried out using the NPT ensemble. Finally, the unrestrained production run was conducted for 100 ns using the NPT ensemble with a 2‐fs time step and 10‐Å cutoff for the nonbond interactions.

All covalent bonds containing a hydrogen atom were constrained using the SHAKE algorithm.^58^ The particle mesh Ewald (PME) method^59^ was used to handle the long‐range electrostatic interactions. A collision frequency of 1.0 ps^−1^ was adopted to control the temperature. The *cpptraj* module^60^ and the python package MDAnalysis^61^ were used for the trajectory analysis.

### QM calculations

A truncated Compound I (Cpd I) model2b with the doublet spin state was used to investigate the reactivity of TES’ potential SOMs. Density functional theory (DFT) calculations were performed to estimate the activation barriers for breaking the corresponding C−H bonds of potential SOMs with the B3LYP functional^62^ implemented in the Gaussian 09 package (Rev. D.01).^63^ For the geometry optimization, the basis set BS1 was used, where the LANL2DZ pseudopotential/basis set was used for the iron and 6–31 g(d) basis set for the rest of atoms.^64^ Transition state (TS) structures were located by flexible potential energy surface (PES) scanning, followed by full geometrical optimization and verified by vibrational frequency calculations. The single‐point (SP) energies were calculated at the same level of theory as geometry optimization using the larger basis set 6–311+g(d,p)/LANL2DZ (denoted as BS2), with the polarizable continuum model (PCM, *ϵ*=4) for recovering the solvent effect.^65^ Dispersion correction was included by using the B3LYP‐D3 functional^66^ with Becke–Johnson damping^67^ for the SP energies. The frequency calculations were employed using the same level of theory and basis set as geometry optimizations and the corresponding thermal zero‐point energies (ZPE) were added to the SP energies. The intrinsic reaction coordinate (IRC) calculations^68^ were performed at the B3LYP level using BS1 to confirm that a TS structure was indeed connecting the reactant and product.

### QM/MM (ONIOM) calculations

For TES and DHT, the potential SOMs are located on the A, B, D rings and the angular methyl groups. In this study, QM/MM calculations were carried out to evaluate the activation barriers of potential SOMs in the protein environment. The QM/MM calculations were accomplished using the ONIOM method in Gaussian 09.^69^ The representative snapshot, which is closest to the major cluster extracted from the MD simulation of each of the CYP3A4 and CYP19A1 complexes, was first energetically minimized by the *pmemd* module of Amber16. The geometrical optimization was then carried out with the ONIOM method. Thereafter, the TS structures for the SOMs were located using the flexible PES scanning. Each TS structure was further confirmed to have only one imaginary frequency. The procedure of preparing input files is similar to our previous study using the TAO package.^70^ Since the reactivities predicted by *S*=1/2 and *S*=3/2 states for Cpd I mediated reactions are generally similar, only the doublet state of Cpd I was considered.2b, 71 The net charge for each layer was determined by the *chargesum* module in TAO. The atoms in the QM region and the corresponding link atoms were optimized using the same level of theory and basis set as in the QM calculations. The SP energy was calculated using BS2 without applying any implicit solvent model and corrected using the same B3LYP‐D3 functional as in the QM calculations. For the molecular mechanics part, the AMBER force field was employed. Only the mechanical embedding scheme was considered in the geometry optimizations and single‐point energy calculations.

The ONIOM calculations were performed using six representative MD snapshots, including four snapshots for CYP3A4; namely, TES‐19‐4D78, DHT‐19‐4D78, TES‐6β‐4K9V and DHT‐6β‐4K9V, and two snapshots for CYP19A1 complexes, for predicting the activation barriers of potential SOMs. For each system, the atoms of the nearest residues in the bottom area of the binding site together with the substrate and some other residues were selected as the QM region atoms. For 19‐4D78, atoms forming the hydrogen‐bond network TES‐water‐Glu308 and DHT‐water‐Leu482, together with Ser119, Ala305, Thr309, Ile369 and Ala370, were included in the QM region. For 6β‐4K9V, the atoms of Ser119, Thr282, Ala305, Ala370 and Leu482 were included in the QM regions. For the QM regions of the CYP19A1 systems, Ile132 and Thr310 were treated as the bottom area residues and Asp309, Val370, Met374 and Leu477 were included.

## Conflict of interest

The authors declare no conflict of interest.

## Supporting information

As a service to our authors and readers, this journal provides supporting information supplied by the authors. Such materials are peer reviewed and may be re‐organized for online delivery, but are not copy‐edited or typeset. Technical support issues arising from supporting information (other than missing files) should be addressed to the authors.

Supplementary

## References

[1a] I. G. Denisov , T. M. Makris , S. G. Sligar , I. Schlichting , Chem. Rev. 2005, 105, 2253–2278; 15941214 10.1021/cr0307143

[1b] F. P. Guengerich , Chem. Res. Toxicol. 2008, 21, 70–83. 18052394 10.1021/tx700079z

[2a] B. Meunier , S. P. de Visser , S. Shaik , Chem. Rev. 2004, 104, 3947–3980; 15352783 10.1021/cr020443g

[2b] S. Shaik , S. Cohen , Y. Wang , H. Chen , D. Kumar , W. Thiel , Chem. Rev. 2010, 110, 949–1017. 19813749 10.1021/cr900121s

[3a] A. B. McQuarters , M. W. Wolf , A. P. Hunt , N. Lehnert , Angew. Chem. Int. Ed. 2014, 53, 4750–4752; 10.1002/anie.201402404 24711286

[3b] P. R. Ortiz de Montellano , Chem. Rev. 2010, 110, 932–948; 19769330 10.1021/cr9002193 PMC2820140

[3c] F. P. Guengerich , ACS Catal. 2018, 8, 10964–10976. 31105987 10.1021/acscatal.8b03401 PMC6519473

[4a] J. T. Groves , G. A. McClusky , J. Am. Chem. Soc. 1976, 98, 859–861;

[4b] F. Ogliaro , N. Harris , S. Cohen , M. Filatov , S. P. de Visser , S. Shaik , J. Am. Chem. Soc. 2000, 122, 8977–8989;

[4c] S. Shaik , S. P. de Visser , F. Ogliaro , H. Schwarz , D. Schröder , Curr. Opin. Chem. Biol. 2002, 6, 556–567; 12413538 10.1016/s1367-5931(02)00363-0

[4d] K. D. Dubey , S. Shaik , Acc. Chem. Res. 2019, 52, 389–399. 30633519 10.1021/acs.accounts.8b00467

[5a] S. Kille , F. E. Zilly , J. P. Acevedo , M. T. Reetz , Nat. Chem. 2011, 3, 738; 21860465 10.1038/nchem.1113

[5b] C. G. Acevedo-Rocha , C. G. Gamble , R. Lonsdale , A. Li , N. Nett , S. Hoebenreich , J. B. Lingnau , C. Wirtz , C. Fares , H. Hinrichs , A. Deege , A. J. Mulholland , Y. Nov , D. Leys , K. J. McLean , A. W. Munro , M. T. Reetz , ACS Catal. 2018, 8, 3395–3410;

[5c] V. B. Urlacher , M. Girhard , Trends Biotechnol. 2019, 37, 882–897. 30739814 10.1016/j.tibtech.2019.01.001

[6] P. M. Dansette , C. Amar , P. Valadon , C. Pons , P. H. Beaune , D. Mansuy , Biochem. Pharmacol. 1991, 41, 553–560. 1997003 10.1016/0006-2952(91)90627-h

[7] J. Kirchmair , A. H. Göller , D. Lang , J. Kunze , B. Testa , I. D. Wilson , R. C. Glen , G. Schneider , Nat. Rev. Drug Discovery 2015, 14, 387. 25907346 10.1038/nrd4581

[8a] F. P. Guengerich in Cytochrome P450: Structure, Mechanism, and Biochemistry, Vol. 2 (Ed. P. R. Ortiz de Montellano), Springer, Cham, 2015, pp. 523–785;

[8b] T. Niwa , N. Murayama , Y. Imagawa , H. Yamazaki , Drug Metab. Rev. 2015, 47, 89–110. 25678418 10.3109/03602532.2015.1011658

[9] J. M. Pascussi , S. Gerbal-Chaloin , L. Drocourt , P. Maurel , M. J. Vilarem , Biochim. Biophys. Acta Gen. Subj. 2003, 1619, 243–253. 10.1016/s0304-4165(02)00483-x 12573484

[10a] K. J. Ryan , Biochim. Biophys. Acta 1958, 27, 658–659; 13535661 10.1016/0006-3002(58)90408-6

[10b] Y. F. Li , W. Hu , S. Q. Fu , J. D. Li , J. H. Liu , J. J. Kavanagh , Int. J. Gynecol. Cancer 2008, 18, 600. 17894799 10.1111/j.1525-1438.2007.01075.x

[11] A. M. H. Brodie , Pharmacol. Ther. 1993, 60, 501–515. 8073072 10.1016/0163-7258(93)90033-a

[12] J. Toppari , J. C. Larsen , P. Christiansen , A. Giwercman , P. Grandjean , L. J. Guillette , B. Jégou , T. K. Jensen , P. Jouannet , N. Keiding , H. Leffers , J. A. McLachlan , O. Meyer , J. Müller , E. Rajpert-De Meyts , T. Scheike , R. Sharpe , J. Sumpter , N. E. Skakkebaek , Environ. Health Perspect. 1996, 104, 741–803. 8880001 10.1289/ehp.96104s4741 PMC1469672

[13] A. D. Mooradian , J. E. Morley , S. G. Korenman , Endocr. Rev. 1987, 8, 1–28. 3549275 10.1210/edrv-8-1-1

[14] D. W. Russell , J. D. Wilson , Annu. Rev. Biochem. 1994, 63, 25–61. 7979239 10.1146/annurev.bi.63.070194.000325

[15a] D. J. Waxman , C. Attisano , F. P. Guengerich , D. P. Lapenson , Arch. Biochem. Biophys. 1988, 263, 424–436; 3259858 10.1016/0003-9861(88)90655-8

[15b] J. A. Krauser , M. Voehler , L.-H. Tseng , A. B. Schefer , M. Godejohann , F. P. Guengerich , Eur. J. Biochem. 2004, 271, 3962–3969. 15373842 10.1111/j.1432-1033.2004.04339.x

[16] Q. Cheng , C. D. Sohl , F. K. Yoshimoto , F. P. Guengerich , J. Biol. Chem. 2012, 287, 29554–29567. 22773874 10.1074/jbc.M112.390047 PMC3436178

[17a] V. H. Teixeira , V. Ribeiro , P. J. Martel , Biochim. Biophys. Acta Proteins Proteomics 2010, 1804, 2036–2045; 10.1016/j.bbapap.2010.06.008 20601222

[17b] I. F. Sevrioukova , T. L. Poulos , Proc. Natl. Acad. Sci. USA 2017, 114, 486–491. 28031486 10.1073/pnas.1616198114 PMC5255590

[18] J. Li , J. Cai , H. Su , H. Du , J. Zhang , S. Ding , G. Liu , Y. Tang , W. Li , Mol. Biosyst. 2016, 12, 868–878. 26761525 10.1039/c5mb00784d

[19a] W. Li , Y. Tang , T. Hoshino , S. Neya , J. Mol. Graphics Modell. 2009, 28, 170–176; 10.1016/j.jmgm.2009.06.002 19596602

[19b] W. Li , H. Ode , T. Hoshino , H. Liu , Y. Tang , H. Jiang , J. Chem. Theory Comput. 2009, 5, 1411–1420. 26609728 10.1021/ct900018t

[20] S. C. Dodani , G. Kiss , J. K. B. Cahn , Y. Su , V. S. Pande , F. H. Arnold , Nat. Chem. 2016, 8, 419. 27102675 10.1038/nchem.2474 PMC4843824

[21] A. H. Follmer , M. Mahomed , D. B. Goodin , T. L. Poulos , J. Am. Chem. Soc. 2018, 140, 16222–16228. 30376314 10.1021/jacs.8b09441

[22] J. Li, H. Zhang, G. Liu, Y. Tang, Y. Tu, W. Li, *Front. Pharmacol* **2018**, *9*. 10.3389/fphar.2018.01065 PMC6167488 30319412

[23] S. Bonomo , F. S. Jørgensen , L. Olsen , Chem. Eur. J. 2017, 23, 2884–2893. 28078726 10.1002/chem.201605094

[24] S. Hur , T. C. Bruice , Proc. Natl. Acad. Sci. USA 2003, 100, 12015–12020. 14523243 10.1073/pnas.1534873100 PMC218705

[25] I. G. Denisov , B. J. Baas , Y. V. Grinkova , S. G. Sligar , J. Biol. Chem. 2007, 282, 7066–7076. 17213193 10.1074/jbc.M609589200

[26a] E. Caspi , T. Arunachalam , P. A. Nelson , J. Am. Chem. Soc. 1983, 105, 6987–6989;

[26b] C. D. Sohl , F. P. Guengerich , J. Biol. Chem. 2010, 285, 17734–17743. 20385561 10.1074/jbc.M110.123711 PMC2878537

[27] Y. Zhang , P. Morisetti , J. Kim , L. Smith , H. Lin , Theor. Chem. Acc. 2008, 121, 313–319.

[28] J. M. Mayer , Acc. Chem. Res. 1998, 31, 441–450.

[29] K. L. M. Drew , J. Reynisson , Eur. J. Med. Chem. 2012, 56, 48–55. 22960693 10.1016/j.ejmech.2012.08.017

[30] T. Lu , F. Chen , J. Comput. Chem. 2012, 33, 580–592. 22162017 10.1002/jcc.22885

[31] W. Humphrey , A. Dalke , K. Schulten , J. Mol. Graphics 1996, 14, 33–38. 10.1016/0263-7855(96)00018-5 8744570

[32] M. Levy , Proc. Natl. Acad. Sci. USA 1979, 76, 6062–6065. 16592733 10.1073/pnas.76.12.6062 PMC411802

[33a] J. C. Hackett , R. W. Brueggemeier , C. M. Hadad , J. Am. Chem. Soc. 2005, 127, 5224–5237; 15810858 10.1021/ja044716w

[33b] F. K. Yoshimoto , F. P. Guengerich , J. Am. Chem. Soc. 2014, 136, 15016–15025. 25252141 10.1021/ja508185d PMC4210144

[34] C. Oostenbrink in Drug Metabolism Prediction (Ed.: J. Kirchmair), Wiley-VCH, Weinheim, 2014, pp. 243–264.

[35] S. S. Oh , C. H. Robinson , J. Steroid Biochem. Mol. Biol. 1993, 44, 389–397. 8476752 10.1016/0960-0760(93)90242-o

[36] G. Jones , P. Willett , R. C. Glen , A. R. Leach , R. Taylor , J. Mol. Biol. 1997, 267, 727–748. 9126849 10.1006/jmbi.1996.0897

[37] J. K. Yano , M. R. Wester , G. A. Schoch , K. J. Griffin , C. D. Stout , E. F. Johnson , J. Biol. Chem. 2004, 279, 38091–38094. 15258162 10.1074/jbc.C400293200

[38] M. Ekroos , T. Sjögren , Proc. Natl. Acad. Sci. USA 2006, 103, 13682. 16954191 10.1073/pnas.0603236103 PMC1564212

[39] I. F. Sevrioukova , T. L. Poulos , J. Biol. Chem. 2012, 287, 3510–3517. 22157006 10.1074/jbc.M111.317081 PMC3271004

[40] P. Kaur , A. R. Chamberlin , T. L. Poulos , I. F. Sevrioukova , J. Med. Chem. 2016, 59, 4210–4220. 26371436 10.1021/acs.jmedchem.5b01146 PMC4998966

[41] I. F. Sevrioukova , T. L. Poulos , J. Med. Chem. 2013, 56, 3733–3741. 23586711 10.1021/jm400288z PMC4534002

[42] I. F. Sevrioukova , T. L. Poulos , Biochemistry 2013, 52, 4474–4481. 23746300 10.1021/bi4005396

[43] I. F. Sevrioukova , Biochemistry 2017, 56, 3058–3067. 28590129 10.1021/acs.biochem.7b00334 PMC5858725

[44] Schrödinger LLC, New York, NY, **2016**.

[45] Prime, Schrödinger LLC, New York, NY, **2016**.

[46] Protassigner, Schrödinger LLC, New York, NY, **2016**.

[47] Maestro 10.5 Release **2016**, Schrödinger, LLC, New York, NY.

[48] S. B. Kirton , C. W. Murray , M. L. Verdonk , R. D. Taylor , Proteins Struct. Funct. Bioinf. 2005, 58, 836–844. 10.1002/prot.20389 15651036

[49] D. Ghosh , C. Egbuta , J. Lo , J. Steroid Biochem. Mol. Biol. 2018, 181, 11–19. 29476820 10.1016/j.jsbmb.2018.02.009 PMC5997392

[50a] A. W. Götz , M. J. Williamson , D. Xu , D. Poole , S. Le Grand , R. C. Walker , J. Chem. Theory Comput. 2012, 8, 1542–1555; 22582031 10.1021/ct200909j PMC3348677

[50b] R. Salomon-Ferrer , A. W. Götz , D. Poole , S. Le Grand , R. C. Walker , J. Chem. Theory Comput. 2013, 9, 3878–3888. 26592383 10.1021/ct400314y

[51] D. A. Case, I. Y. Ben-Shalom, S. R. Brozell, D. S. Cerutti, T. E. Cheatham III, V. W. D. Cruzeiro, T. A. Darden, R. E. Duke, D. Ghoreishi, M. K. Gilson, H. Gohlke, A. W. Goetz, D. Greene, R. Harris, N. Homeyer, S. Izadi, A. Kovalenko, T. Kurtzman, T. S. Lee, S. LeGrand, P. Li, C. Lin, J. Liu, T. Luchko, R. Luo, D. J. Mermelstein, K. M. Merz, Y. Miao, G. Monard, C. Nguyen, H. Nguyen, I. Omelyan, A. Onufriev, F. Pan, R. Qi, D. R. Roe, A. Roitberg, C. Sagui, S. Schott-Verdugo, J. Shen, C. L. Simmerling, J. Smith, R. Salomon-Ferrer, J. Swails, R. C. Walker, J. Wang, H. Wei, R. M. Wolf, X. Wu, L. Xiao, D. M. York, P. A. Kollman, *Amber 2016*; *University of California*, *San Francisco*, *CA*, **2016**.

[52] J. A. Maier , C. Martinez , K. Kasavajhala , L. Wickstrom , K. E. Hauser , C. Simmerling , J. Chem. Theory Comput. 2015, 11, 3696–3713. 26574453 10.1021/acs.jctc.5b00255 PMC4821407

[53] J. Wang , R. M. Wolf , J. W. Caldwell , P. A. Kollman , D. A. Case , J. Comput. Chem. 2004, 25, 1157–1174. 15116359 10.1002/jcc.20035

[54a] C. R. Søndergaard , M. H. M. Olsson , M. Rostkowski , J. H. Jensen , J. Chem. Theory Comput. 2011, 7, 2284–2295; 26606496 10.1021/ct200133y

[54b] M. H. M. Olsson , C. R. Søndergaard , M. Rostkowski , J. H. Jensen , J. Chem. Theory Comput. 2011, 7, 525–537. 26596171 10.1021/ct100578z

[55] J. Wang , P. Cieplak , P. A. Kollman , J. Comput. Chem. 2000, 21, 1049–1074.

[56] W. L. Jorgensen , J. Chandrasekhar , J. D. Madura , R. W. Impey , M. L. Klein , J. Chem. Phys. 1983, 79, 926–935.

[57] K. Shahrokh , T. E. Cheatham 3rd , G. S. Yost , Biochim. Biophys. Acta Gen. Subj. 2012, 1820, 1605–1617. 10.1016/j.bbagen.2012.05.011 PMC3404218 22677141

[58] J.-P. Ryckaert , G. Ciccotti , H. J. C. Berendsen , J. Comput. Phys. 1977, 23, 327–341.

[59a] T. Darden , D. York , L. Pedersen , J. Chem. Phys. 1993, 98, 10089–10092;

[59b] U. Essmann , L. Perera , M. L. Berkowitz , T. Darden , H. Lee , L. G. Pedersen , J. Chem. Phys. 1995, 103, 8577–8593.

[60] D. R. Roe , T. E. Cheatham , J. Chem. Theory Comput. 2013, 9, 3084–3095. 26583988 10.1021/ct400341p

[61a] N. Michaud-Agrawal , E. J. Denning , T. B. Woolf , O. Beckstein , J. Comput. Chem. 2011, 32, 2319–2327; 21500218 10.1002/jcc.21787 PMC3144279

[61b] M. L. R. J. Gowers , J. Barnoud , T. J. E. Reddy , M. N. Melo , S. L. Seyler , D. L. Dotson , J. Domanski , S. Buchoux , I. M. Kenney , and O. Beckstein , Proceedings of the 15th Python in Science Conference, Austin, 2016, pp. 98–105.

[62a] C. Lee , W. Yang , R. G. Parr , Phys. Rev. B 1988, 37, 785–789; 10.1103/physrevb.37.785 9944570

[62b] A. D. Becke , J. Chem. Phys. 1993, 98, 5648–5652.

[63] M. Frisch, G. Trucks, H. Schlegel, G. Scuseria, M. Robb, J. Cheeseman, G. Scalmani, V. Barone, B. Mennucci, G. Petersson in Gaussian09. Revision D. 01, Gaussian Inc., Wallingford, CT, USA, Vol. **2013**.

[64] P. J. Hay , W. R. Wadt , J. Chem. Phys. 1985, 82, 270–283.

[65a] S. Miertuš , E. Scrocco , J. Tomasi , Chem. Phys. 1981, 55, 117–129;

[65b] E. Cancès , B. Mennucci , J. Tomasi , J. Chem. Phys. 1997, 107, 3032–3041.

[66] S. Grimme , J. Antony , S. Ehrlich , H. Krieg , J. Chem. Phys. 2010, 132, 154104. 20423165 10.1063/1.3382344

[67] S. Grimme , S. Ehrlich , L. Goerigk , J. Comput. Chem. 2011, 32, 1456–1465. 21370243 10.1002/jcc.21759

[68] C. Gonzalez , H. B. Schlegel , J. Phys. Chem. 1990, 94, 5523–5527.

[69a] S. Dapprich , I. Komáromi , K. S. Byun , K. Morokuma , M. J. Frisch , J. Mol. Struct. 1999, 461–462, 1–21;

[69b] L. W. Chung , W. M. C. Sameera , R. Ramozzi , A. J. Page , M. Hatanaka , G. P. Petrova , T. V. Harris , X. Li , Z. Ke , F. Liu , H.-B. Li , L. Ding , K. Morokuma , Chem. Rev. 2015, 115, 5678–5796. 25853797 10.1021/cr5004419

[70] P. Tao , H. B. Schlegel , J. Comput. Chem. 2010, 31, 2363–2369. 20340103 10.1002/jcc.21524

[71] S. Shaik , D. Kumar , S. P. de Visser , A. Altun , W. Thiel , Chem. Rev. 2005, 105, 2279–2328. 15941215 10.1021/cr030722j

