# Supplementary material for: Mechanistic Insights into the Regio‐ and Stereoselectivities of Testosterone and Dihydrotestosterone Hydroxylation Catalyzed by CYP3A4 and CYP19A1
Source: Chemistry. 2020 Apr 28;26(28):6214–23. doi: 10.1002/chem.201905272 (PMC7318132; doi:10.1002/chem.201905272)
Supplement: Supplementary file 1 — Supplementary [file CHEM-26-6214-s001.pdf]

Chemistry–A European Journal

Supporting Information

Mechanistic Insights into the Regio- and Stereoselectivities of Testosterone and Dihydrotestosterone Hydroxylation Catalyzed by CYP3A4 and CYP19A1

Junhao Li,[a] Yun Tang,[b] Weihua Li,*[b] and Yaoquan Tu*[a]

Supplementary Information

Mechanistic Insights into the Regio- and Stereoselectivities of Testosterone and Dihydrotestosterone Hydroxylation Catalyzed by CYP3A4 and CYP19A1

Junhao Li[1], Yun Tang[2], Weihua Li[2*], Yaoquan Tu[1*]

[1] *Division of Theoretical Chemistry and Biology, School of Engineering Sciences in Chemistry, Biotechnology and Health (CBH), KTH Royal Institute of Technology, SE-106 91 Stockholm, Sweden*

[2] *Shanghai Key Laboratory of New Drug Design, School of Pharmacy, East China University of Science and Technology, Shanghai 200237, China*

Supplementary Tables and Figures

Table S1. C–H bond dissociation energies (BDEs) of Testosterone (see the figure below the table for atom numbering).

Site	TES (a.u.)	ZPE_TES (a.u.)	Radical (a.u.)	Radical_ZPE (a.u.)	BDE (kcal/mol)	BDE_ZPE (kcal/mol)
**6**	-891.6049433576	0.440041	-890.9666165023	0.426361	-85.4	-76.8
**2**	/	/	-890.9510130360	0.426199	-95.2	-86.5
**14**	/	/	-890.9499002841	0.425591	-95.9	-86.8
**17**	/	/	-890.9489028468	0.426282	-96.5	-87.9
**9**	/	/	-890.9447531420	0.425993	-99.1	-90.3
**11**	/	/	-890.9414618391	0.424996	-101.2	-91.7
**16**	/	/	-890.9418882407	0.425424	-100.9	-91.7
**8**	/	/	-890.9422017742	0.42591	-100.7	-91.8
**15**	/	/	-890.9398723118	0.425091	-102.2	-92.8
**1**	/	/	-890.9392863187	0.425187	-102.5	-93.2
**7**	/	/	-890.9372149863	0.425059	-103.8	-94.4
**12**	/	/	-890.9373718148	0.425382	-103.7	-94.5
**18**	/	/	-890.9359319543	0.424884	-104.6	-95.1
**19**	/	/	-890.9343160860	0.424932	-105.7	-96.2
**4**	/	/	-890.9175868721	0.426968	-116.2	-107.9

SP energy calculated using B3LYP/6-311++g(2d,2p);

ZPE calculated using B3LYP/6-31g(d,p);

SP energy of the hydrogen atom: -0.50225698153 a.u. (B3LYP/6-311++g(2d,2p) level)

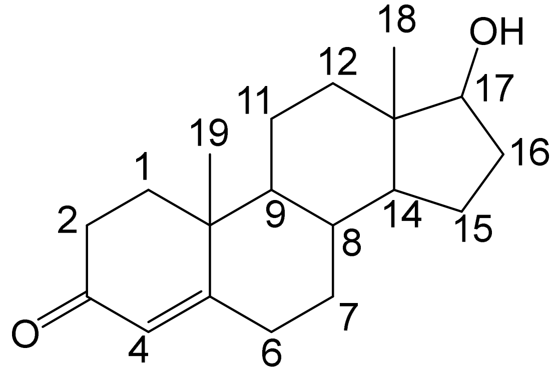

Table S2. Energies for each QM species calculated at the B3LYP level

Sites	SP_TS[a] (a.u.)	ZPE_TS[b] (a.u.)	SP_RC[a] (a.u.)	ZPE_RC[b] (a.u.)	EA[c] (kcal/mol)	ZPE_EA[d] (kcal/mol)
1α	-2477.6661382414	0.722123	-2477.7035822272	0.729773	23.5	18.7
1β	-2477.6662917822	0.722188	-2477.7036632453	0.729175	23.5	19.1
2α	-2477.6703818226	0.722049	-2477.7018753081	0.729065	19.8	15.4
2β	-2477.6752462858	0.722356	-2477.7029445109	0.729312	17.4	13.0
6α	-2477.6676008546	0.722836	-2477.7029974288	0.729405	22.2	18.1
6β	-2477.6794527600	0.722902	-2477.7025697197	0.729558	14.5	10.3
15α	-2477.6671319848	0.722015	-2477.7033265201	0.729686	22.7	17.9
15β	-2477.6732158743	0.722924	-2477.7044562323	0.729407	19.6	15.5
18	-2477.6665876695	0.723949	-2477.7044531067	0.729319	23.8	20.4
19	-2477.6611218774	0.722142	-2477.7045854482	0.729350	27.3	22.8
8	-2477.6640699786	0.722385	-2477.7046339968	0.729356	25.5	21.1

[a]. SP energy calculated using 6-311+g(d,p)/LANL2DZ/PCM, see Method for details

[b]. ZPE energy calculated using 6-31g(d)/LANL2DZ

[c]. activation barrier

[d]. activation barrier with ZPE correction

Table S3. Energies for each QM species calculated at the B3LYP-D3 level

Sites	SP_TS[a] (a.u.)	ZPE_TS[b] (a.u.)	SP_RC[a] (a.u.)	ZPE_RC[b] (a.u.)	EA[c] (kcal/mol)	ZPE_EA[d] (kcal/mol)
1α	-2477.9215788775	0.722123	-2477.9521764615	0.729773	19.2	14.4
1β	-2477.9186995009	0.722188	-2477.9471586413	0.729175	17.9	13.5
2α	-2477.9143914801	0.722049	-2477.9427504920	0.729065	17.8	13.4
2β	-2477.9219271979	0.722356	-2477.9446692453	0.729312	14.3	9.9
6α	-2477.9166150133	0.722836	-2477.9425963025	0.729405	16.3	12.2
6β	-2477.9338127590	0.722902	-2477.9489783868	0.729558	9.5	5.3
15α	-2477.9200193996	0.722015	-2477.9515103941	0.729686	19.8	14.9
15β	-2477.9265182724	0.722924	-2477.9492329441	0.729407	14.3	10.2
18	-2477.9231321023	0.723949	-2477.9492329556	0.729319	16.4	13.0
19	-2477.9126628059	0.722142	-2477.9490130403	0.729350	22.8	18.3
8	-2477.9261967660	0.722385	-2477.9490732275	0.729356	14.4	10.0

[a]. SP energy calculated using 6-311+g(d,p)/LANL2DZ/PCM, see Method for details

[b]. ZPE energy calculated using 6-31g(d)/LANL2DZ

[c]. activation barrier

[d]. activation barrier with ZPE correction

Table S4. Energies for each species in the CYP3A4 17–OH_UP systems calculated with the ONIOM model (unit: a.u.)

		BS1: b3lyp/6-31g(d)					BS2: b3lyp/6-311+g(d,p)				
		low_model	high_model	low_real	ONIOM extrapolation	ZPE	low_model	high_model	low_real	ONIOM extrapolation	QMD3[a]
	RC	0.12610464	-3331.61932008	-62.48135297	-3394.22677769	9.793738	0.1261047076	-3332.53581309	-62.48135287	-3395.14327067	-3332.86168274
TES	19	16.14564303	-3331.56966836	-46.45901555	-3394.17432693	9.786219	16.1457104454	-3332.48579069	-46.45894814	-3395.09044927	-3332.82121526
	6β	9.68101995	-3331.58037527	-52.92565897	-3394.18705419	9.786083	9.6810199528	-3332.50190635	-52.92565897	-3395.10858528	-3332.83741669
	RC	0.13643093	-3234.84536051	-62.81031454	-3297.79210599	9.904145	0.1371291508	-3235.74286479	-62.81031471	-3298.69030865	-3236.05511906
DHT	19	12.88410548	-3234.81075858	-50.06175138	-3297.75661544	9.894908	14.8687564401	-3235.69850718	-48.07710038	-3298.64436400	-3236.02268900
	4β	10.91492715	-3234.80918908	-52.02613973	-3297.75025595	9.899001	10.9149271477	-3235.69834355	-52.02613973	-3298.63941043	-3236.02328714

[a]. The SP energy of the QM region atoms with capped hydrogen atoms calculated at the B3LYP-D3 level

Table S5. Activation barriers calculated at different levels of theory (kcal/mol), the geometry features (Å or °) of the TS structures and the smallest vibrational frequency (“min_freq”) for the stationary points in the CYP3A4 17–OH_UP systems

		Activation Barriers					Geometry				min_freq
		BS1	BS1:ZPE	BS2	BS2:ZPE	BS2:D3:ZPE	H-OE	FE-OE	$H\text{-}C_{abs}$	H-OE-FE	
	RC										16.1
TES	19	32.9	28.2	33.1	28.4	22.4	1.16	1.77	1.43	121.1	-1933.6
	6β	24.9	20.1	21.8	17.0	10.9	1.27	1.76	1.32	128.1	-1869.0
	RC										18.8
DHT	19	22.3	16.5	28.8	23.0	15.5	1.17	1.76	1.42	123.6	-1932.5
	4β	26.3	23.0	31.9	28.7	20.7	1.22	1.72	1.37	126.1	-2055.6

Table S6. Energies for each species in the CYP3A4 17–OH_DOWN systems calculated with the ONIOM model (unit: a.u.)

		BS1: b3lyp/6-31g(d)					BS2: b3lyp/6-311+g(d,p)				
		low_model	high_model	low_real	ONIOM extrapolation	ZPE	low_model	high_model	low_real	ONIOM extrapolation	QMD3[a]
TES	RC	0.15274456	-2987.33194127	-63.63230879	-3051.11699462	8.762072	0.1527445078	-2988.09784910	-63.63230890	-3051.88290251	-2988.40034766
	19	21.51433947	-2987.28222461	-42.26873552	-3051.06529960	8.755342	21.5144522540	-2988.04903932	-42.26862263	-3051.83211420	-2988.36033178
	6β	14.88066078	-2987.29191012	-48.90298030	-3051.07555120	8.756700	14.8807114249	-2988.06916412	-48.90292957	-3051.85280512	-2988.37911855
	8	11.37371217	-2987.27840370	-52.40470880	-3051.05682467	8.755293	11.3737121695	-2988.05164686	-52.40470881	-3051.83006784	-2988.37374275
DHT	RC	0.18439867	-2988.53599411	-62.94931070	-3051.66970348	10.405372	0.1843988988	-2989.30319181	-62.94931049	-3052.43690119	-2989.60860325
	19	17.69602375	-2988.48575392	-45.43050983	-3051.61228749	10.398067	17.6959889954	-2989.26083536	-45.43054456	-3052.38736891	-2989.57418765
	18	12.37322678	-2988.48530613	-50.75234489	-3051.61087780	10.398424	12.3732530334	-2989.25797155	-50.75231858	-3052.38354316	-2989.57233489
	8	10.68778201	-2988.48036627	-52.43704021	-3051.60518849	10.397582	10.6877595990	-2989.24458334	-52.43706267	-3052.36940561	-2989.56856414

[a]. The SP energy of the QM region atoms with capped hydrogen atoms calculated at the B3LYP-D3 level

Table S7. Activation barriers calculated at different levels of theory (kcal/mol), the geometry features (Å or °) of the TS structures and the smallest vibrational frequency ("min_freq") for the stationary points in the CYP3A4 17–OH_DOWN systems

		Activation Barriers					Geometry				min_freq
		BS1	BS1:ZPE	BS2	BS2:ZPE	BS2:ZPE:D3	H-OE	FE-OE	$H\text{-}C_{abs}$	H-OE-FE	
TES	RC										16.4
	19	32.4	28.2	31.9	27.6	22.1	1.13	1.76	1.42	129.5	-1643.0
	6β	26.0	22.6	18.9	15.5	10.8	1.18	1.72	1.41	125.9	-1326.3
	8	37.8	33.5	33.2	28.9	16.6	1.21	1.77	1.40	149.8	-1874.0
DHT	RC										17.6
	19	36.0	31.4	31.1	26.5	21.5	1.16	1.75	1.38	138.9	-1572.8
	18	36.9	32.6	33.5	29.1	23.5	1.20	1.76	1.40	119.9	-1973.4
	8	40.5	35.6	42.4	37.5	25.8	1.22	1.77	1.41	153.5	-1922.6

Table S8. Energies for each species in the CYP19A1 systems calculated with the ONIOM model (unit: a.u.)

		BS1: b3lyp/6-31g(d)					BS2: b3lyp/6-311+g(d,p)				
		low_model	high_model	low_real	oniom: extr	ZPE	low_model	high_model	low_real	oniom: extr	QMD3[a]
TES	RC	0.10304194	-3467.09590828	-60.53623334	-3527.73518356	9.691382	0.1030419445	-3468.04682472	-60.53623334	-3528.68610000	-3468.40490773
	19	16.22900321	-3467.08987713	-44.40925453	-3527.72813487	9.683744	16.2290034545	-3468.01245587	-44.40925433	-3528.65071365	-3468.37379514
	1β	16.76450272	-3467.08051509	-43.87174159	-3527.71675940	9.683969	16.7645027187	-3468.00087666	-43.87174159	-3528.63712097	-3468.36438563
	2β	23.68509404	-3467.08497651	-36.94215769	-3527.71222825	9.686195	23.6850940441	-3468.00339621	-36.94215769	-3528.63064795	-3468.36914278
DHT	RC	0.11560553	-3468.34320413	-59.71908021	-3528.17788987	9.415460	0.1156055341	-3469.26569997	-59.71908021	-3529.10038571	-3469.62794339
	19	20.30618648	-3468.29960458	-39.54127138	-3528.14706244	9.406490	20.3061864845	-3469.22830969	-39.54127138	-3529.07576755	-3469.59611366
	1β	21.72497302	-3468.28644343	-38.11525225	-3528.12666870	9.407586	21.7249733347	-3469.21564509	-38.11525194	-3529.05587037	-3469.58397352
	2β	33.38385567	-3468.29811764	-26.44888701	-3528.13086033	9.410147	33.3839356892	-3469.22547036	-26.44880695	-3529.05821300	-3469.59559298

[a]. The SP energy of the QM region atoms with capped hydrogen atoms calculated at the B3LYP-D3 level

Table S9. Activation barriers calculated at different levels of theory (kcal/mol), the geometry features (Å or °) of the TS structures and the smallest vibrational frequency ("min_freq") for the stationary points in the CYP19A1 systems

		Activation Barriers					Geometry				min_freq
		BS1	BS1:ZPE	BS2	BS2:ZPE	BS2:ZPE:D3	H-OE	FE-OE	$H\text{-}C_{abs}$	H-OE-FE	
TES	RC										17.0
	19	4.4	-0.4	22.2	17.4	15.4	1.18	1.75	1.33	123.8	-1432.1
	1β	11.6	6.9	30.7	26.1	22.7	1.16	1.78	1.42	126.8	-1399.4
	2β	14.4	11.1	34.8	31.5	26.7	1.12	1.73	1.57	136.1	-1254.3
DHT	RC										17.4
	19	19.3	13.7	15.4	9.8	6.3	1.14	1.80	1.43	122.6	-1225.6
	1β	32.1	27.2	27.9	23.0	19.2	1.13	1.75	1.50	129.9	-1116.4
	2β	29.5	26.2	26.5	23.1	18.2	1.08	1.73	1.61	133.8	-568.1

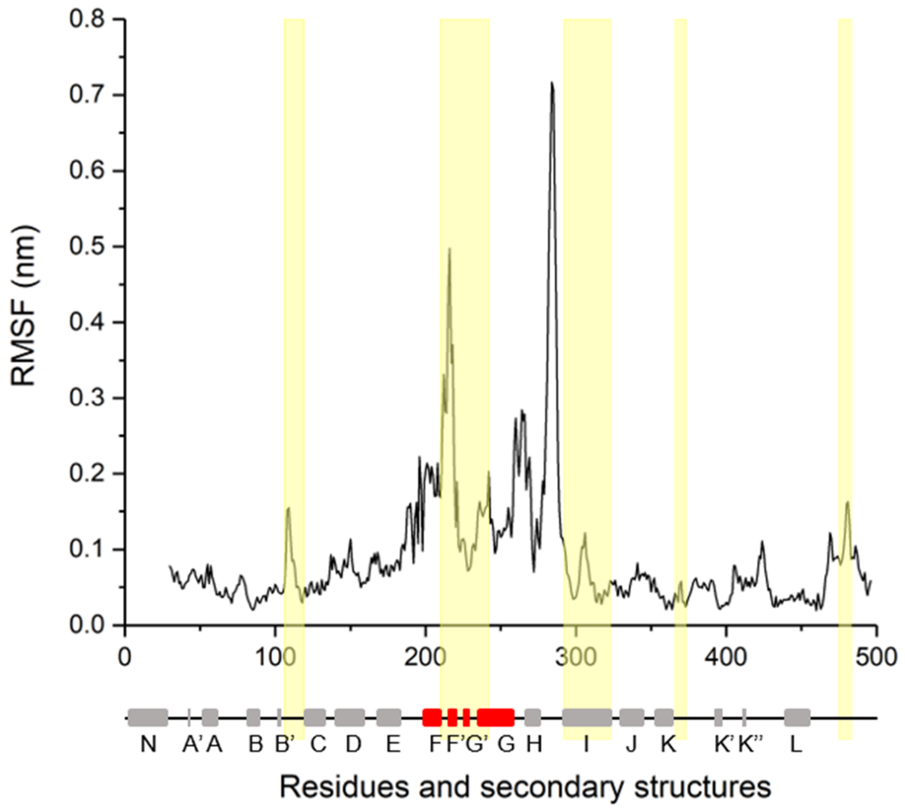

Figure S1. Root mean square fluctuation (RMSF) for each residue averaged over the 11 selected crystal structures of CYP3A4. The active site residues are highlighted in light yellow box, which are comprised of the B'-C loop, F-F'-G'-G segmentation, middle part of the I-helix, K-β3 loop and C-terminal loop.

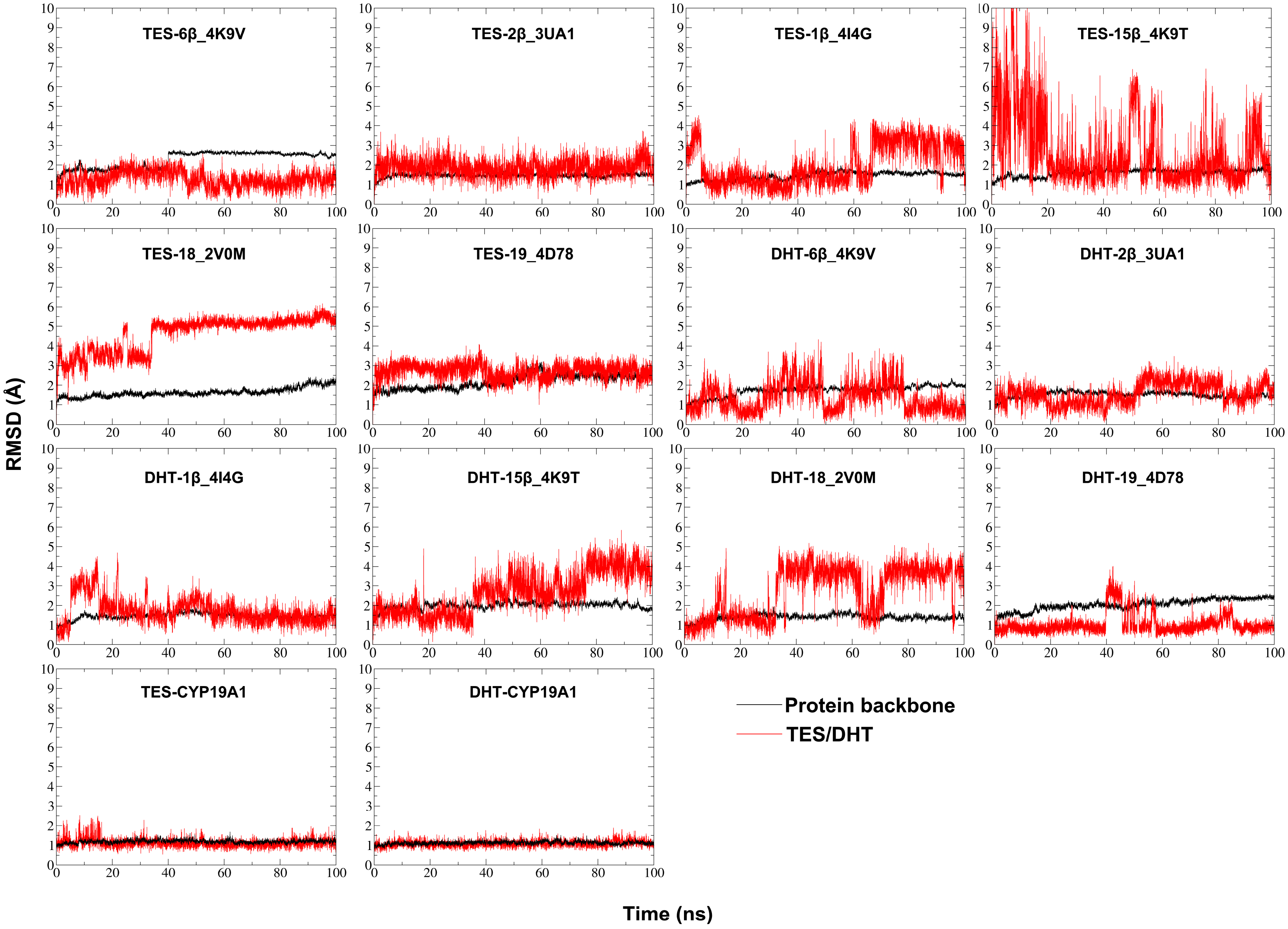

Figure S2. Time evolutions of RMSDs during the 100-ns MD simulations for all systems

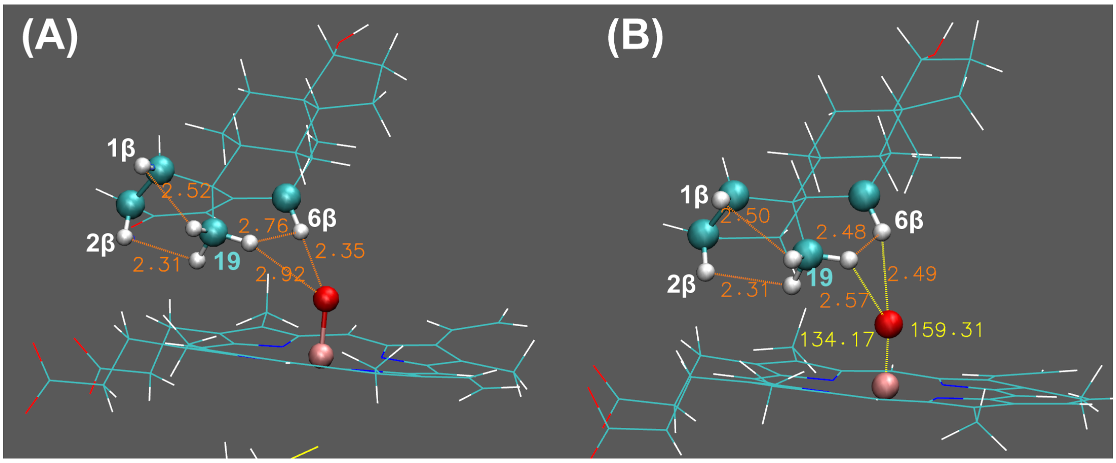

Figure S3. Structures of TES (A) and DHT (B) with the heme moiety obtained from the MD simulations of the CYP3A4 17–OH_UP systems. The distance (Å) and angles (°) are labeled in orange and yellow, respectively. The 19, 1β, 2β and 6β sites are labeled and depicted in spheres.

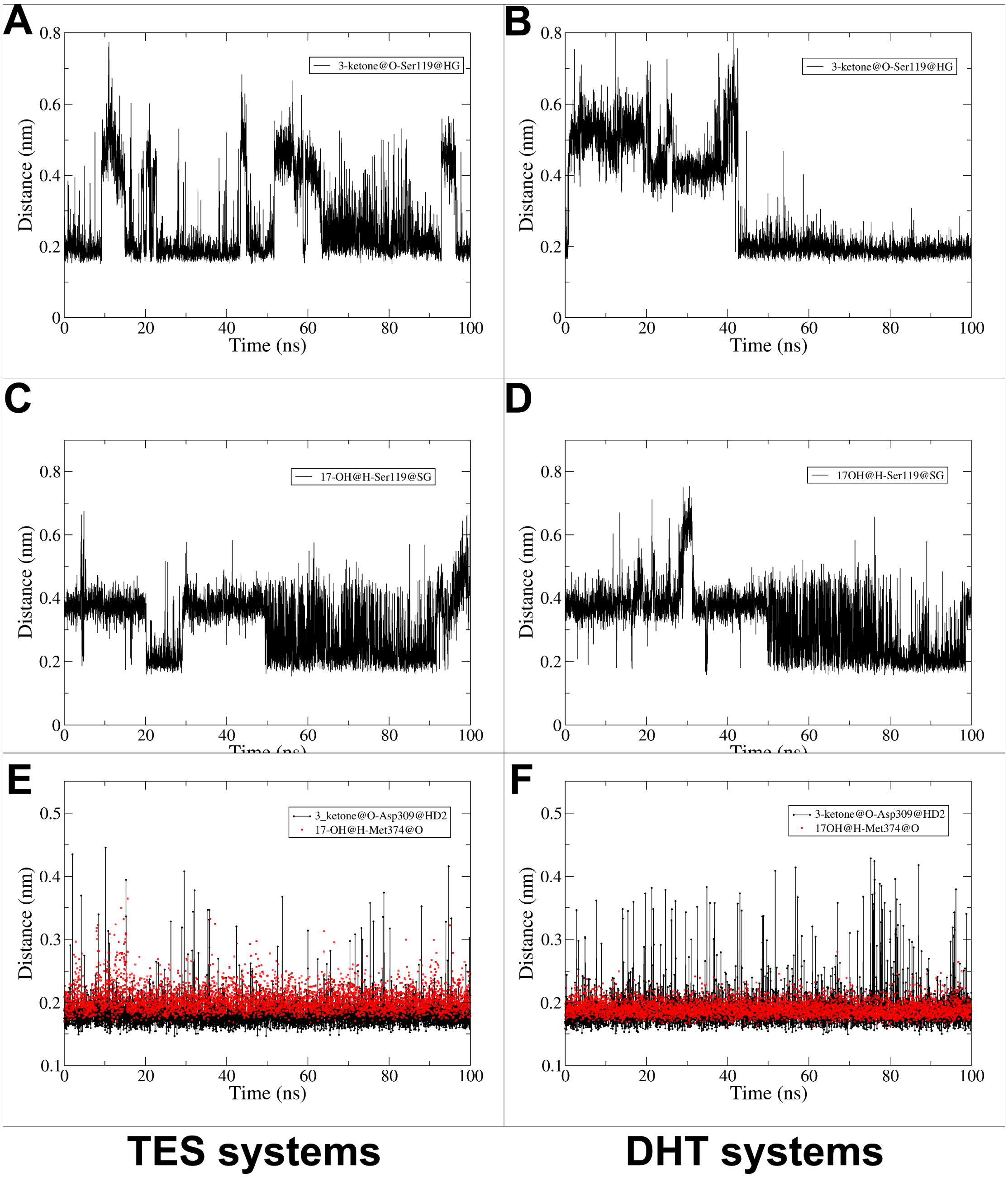

Figure S4. Time evolutions of the hydrogen-bond distances between the key residues and TES/DHT during the 100-ns MD simulations, (A), (B): in the 19_4D78 systems, corresponding to the 17–OH_UP binding modes; (C), (D): in the 6β_4K9V systems, corresponding to the 17–OH_DOWN binding modes; (E), (F): in the CYP19A1 systems with two stable hydrogen bonds.

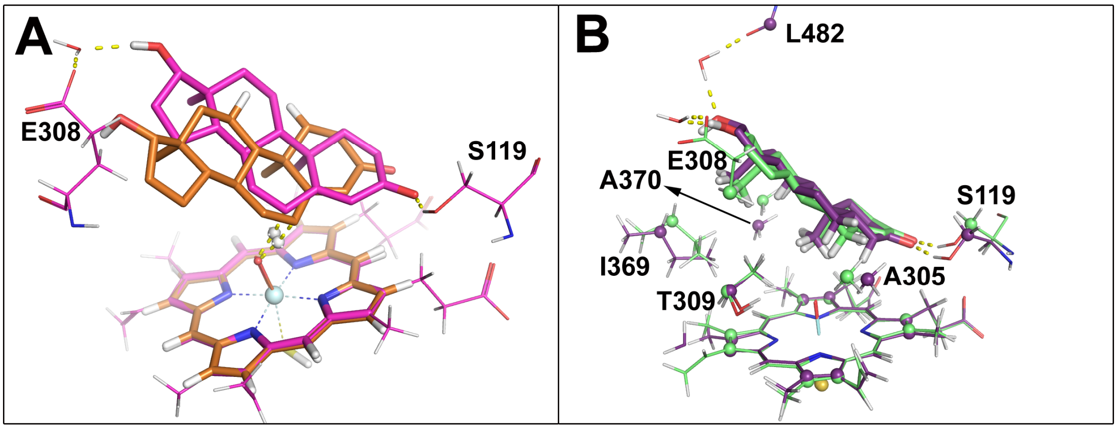

Figure S5. (A) Comparison of the TS structures for the 6β site of TES in the 19_4D78 system from the ONOIM calculations (in magenta) and that from the QM calculation (in orange). (B) Comparison of the RC structures for TES (in green) and DHT (in purple) in the 19_4D78 system.

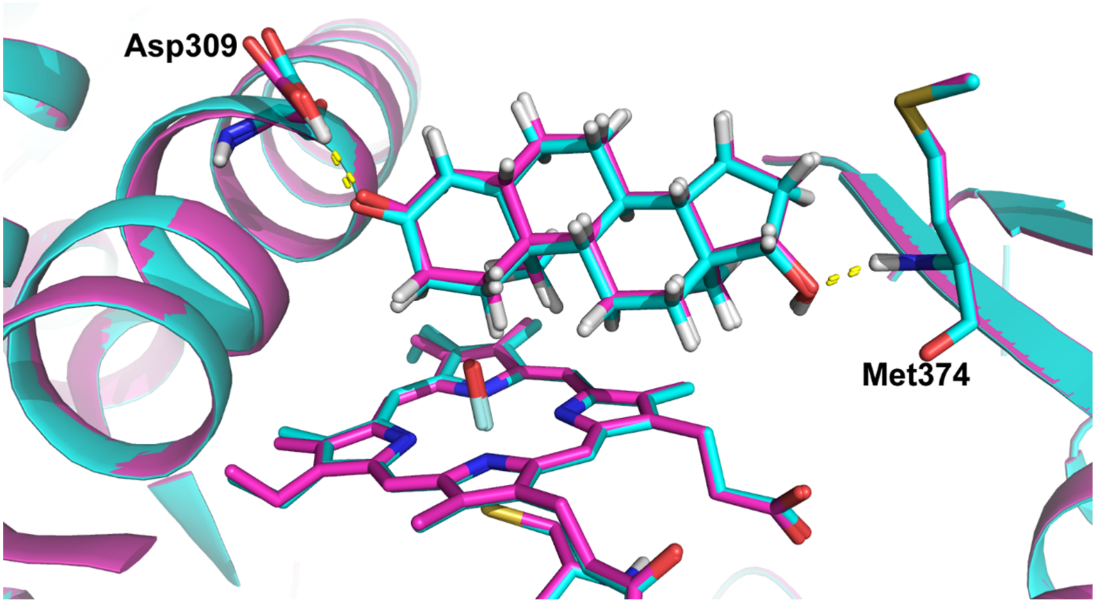

Figure S6. The reactant complexes of CYP19A1–TES (cyan) and CYP19A1–DHT (magenta).

Coordinates for the stationary points

QM Calculations

RC_6 β

S	4.396432	−1.981218	2.238496
N	3.138386	−2.414837	−0.380428
C	4.060647	−2.169612	−1.372549
C	2.714287	−3.711212	−0.574411
C	4.238735	−3.345059	−2.189568
C	3.408918	−4.301261	−1.692549
C	1.742901	−4.350928	0.177906
H	1.500419	−5.375051	−0.089495
C	1.045183	−3.789279	1.243953
N	1.217671	−2.516324	1.718640
C	0.331299	−2.379283	2.756810
C	−0.412330	−3.600559	2.947048
C	0.033501	−4.478955	2.006955
C	0.161012	−1.233053	3.521913
H	−0.608776	−1.244746	4.286954
C	0.876501	−0.052217	3.362962
C	0.701838	1.115389	4.193808
C	2.301621	1.439263	2.643491
N	1.849643	0.166356	2.424591
C	1.595514	2.042073	3.749021
C	3.295575	2.075567	1.906754
H	3.558925	3.087886	2.198226
C	3.964885	1.532443	0.820071
C	4.964728	2.226233	0.044718
N	3.760174	0.271887	0.315768
C	4.596988	0.158821	−0.764108
C	4.725387	−0.969211	−1.565215
H	5.424353	−0.914178	−2.394186
Fe	2.379045	−1.069220	0.929817
C	5.352039	1.375600	−0.943741
O	1.234693	−0.544954	−0.102643
C	−3.321670	2.589145	1.506574
C	−2.656216	1.760894	0.374828
C	−1.196379	2.242655	0.155040
H	−0.690724	1.687673	−0.639514
H	−1.170911	3.307741	−0.098755
H	−0.600281	2.099745	1.061132
H	−3.208547	3.658839	1.300525
H	−4.400535	2.380133	1.499224
C	−2.775564	2.285204	2.904942
H	−3.345845	2.805449	3.682399
H	−1.736552	2.630493	3.003956
C	−2.772208	0.799247	3.221820
O	−2.710213	0.387365	4.379463
C	−2.742497	−0.112097	2.074507
C	−2.610797	0.287259	0.788971
C	−2.323906	−0.728862	−0.287053
H	−2.446762	−1.739796	0.118234
H	−1.261306	−0.638836	−0.559979
C	−3.188363	−0.535334	−1.540104
H	−4.235203	−0.782623	−1.307384
H	−2.863731	−1.238184	−2.316878
C	−3.119157	0.906792	−2.060487
H	−2.081827	1.107361	−2.365987
C	−3.527333	1.899709	−0.932935
H	−4.547358	1.600047	−0.637126
C	−3.630256	3.358889	−1.444750
H	−2.627747	3.753527	−1.646677
H	−4.052657	3.996588	−0.660424
C	−4.495704	3.507610	−2.713528
H	−4.481752	4.551235	−3.053860
H	−5.542092	3.274069	−2.467184
C	−4.027251	2.560450	−3.826371
C	−2.650389	3.001011	−4.368158
H	−2.726478	4.009409	−4.792651
H	−1.884669	3.036763	−3.589131
H	−2.274159	2.333377	−5.152053
C	−4.042094	1.118414	−3.267916
H	−5.069191	0.957723	−2.895697
C	−3.892628	0.221148	−4.516634
H	−4.323455	−0.773316	−4.363856
H	−2.835058	0.071440	−4.766596
C	−4.624718	1.016067	−5.637738
H	−5.508666	0.493542	−6.016570
H	−3.969485	1.178304	−6.503431
C	−5.011702	2.390118	−5.014512
H	−6.028389	2.333916	−4.606830
O	−5.073688	3.462732	−5.942031
H	−4.233710	3.482744	−6.426790
H	3.967221	−3.256523	2.373126
H	6.085149	1.535701	−1.723883
H	3.262838	−5.315524	−2.041525
H	5.310886	3.232218	0.244515
H	4.917677	−3.411930	−3.030001
H	−0.280087	−5.500492	1.833702
H	1.773722	3.042665	4.121930
H	−0.023872	1.191198	4.992855
H	−1.166524	−3.748559	3.709183

H	−2.705032	−1.170494	2.322383

TS_6 β

S	49.004873	41.903108	44.896372
N	47.265177	41.424734	42.572169
C	47.975126	41.658529	41.417693
C	46.776358	40.145432	42.461496
C	47.953838	40.487136	40.574470
C	47.212236	39.548185	41.221753
C	45.966773	39.520860	43.399748
H	45.654132	38.502881	43.186418
C	45.533691	40.078717	44.599092
N	45.819304	41.350729	45.030047
C	45.234454	41.464470	46.267442
C	44.555110	40.241442	46.619559
C	44.739178	39.380345	45.580793
C	45.269434	42.600418	47.066902
H	44.721344	42.570764	48.003414
C	45.929368	43.783418	46.750236
C	45.957682	44.951305	47.596984
C	47.131162	45.279813	45.703712
N	46.644212	44.003523	45.604205
C	46.709541	45.882217	46.946039
C	47.920442	45.920808	44.753294
H	48.233597	46.936412	44.976917
C	48.339342	45.379936	43.544444
C	49.150964	46.073139	42.569477
N	48.044698	44.116185	43.107624
C	48.644262	43.990160	41.884204
C	48.607078	42.849210	41.090749
H	49.126360	42.889203	40.137868
Fe	46.855069	42.757735	44.043303
C	49.337523	45.209810	41.535381
O	45.535959	43.415566	43.148247
C	41.301823	45.989216	45.074140
C	42.304983	45.334839	44.086434
C	43.664672	46.081451	44.148856
H	44.407709	45.634863	43.483896
H	43.542185	47.137783	43.885828
H	44.083189	46.037942	45.158797
H	41.216361	47.059626	44.859450
H	40.308060	45.552999	44.900509
C	41.670390	45.802119	46.550856
H	40.877439	46.169194	47.211750
H	42.569845	46.380016	46.805614
C	41.953676	44.350555	46.906433
O	41.917452	43.948082	48.067667
C	42.328334	43.468524	45.800954
C	42.538692	43.879501	44.514975
C	43.073449	42.929176	43.540453
H	43.078525	41.898638	43.905902
H	44.357803	43.171031	43.469635
C	42.620706	43.056355	42.096026
H	41.626850	42.585961	41.998777
H	43.298100	42.485635	41.450635
C	42.527921	44.513153	41.622546
H	43.548838	44.912463	41.567069
C	41.694129	45.351140	42.635379
H	40.720680	44.836874	42.716065
C	41.392781	46.780388	42.115898
H	42.303324	47.389226	42.152235
H	40.676059	47.270594	42.783984
C	40.824206	46.814082	40.682072
H	40.692107	47.854937	40.358962
H	39.823600	46.356927	40.677508
C	41.722385	46.044291	39.704753
C	43.065267	46.779765	39.506815
H	42.882290	47.778743	39.092636
H	43.612168	46.913483	40.443518
H	43.737015	46.248986	38.822148
C	41.878883	44.601048	40.235566
H	40.849504	44.219348	40.356224
C	42.492567	43.820099	39.052203
H	42.253245	42.752793	39.093673
H	43.586594	43.898158	39.059205
C	41.889304	44.512351	37.794641
H	41.243176	43.845563	37.215049
H	42.677529	44.848678	37.108597
C	41.094491	45.746060	38.316784
H	40.045232	45.466792	38.471953
O	41.028058	46.833926	37.407361
H	41.935149	47.051391	37.141146
H	48.612912	40.631409	45.138226
H	49.894490	45.362909	40.619862
H	46.968870	38.542743	40.902703
H	49.521173	47.084319	42.680566
H	48.446823	40.412024	39.613648
H	44.386195	38.362131	45.477816
H	46.965829	46.884936	47.263593
H	45.463518	45.027520	48.556869
H	44.017454	40.080383	47.545088

H	42.496483	42.429873	46.071510

RC_6 α

S	3.588233	−4.125631	1.433232
N	3.527258	−2.379236	−1.060682
C	4.748477	−1.973245	−1.539924
C	2.957827	−3.119013	−2.064378
C	4.956144	−2.477507	−2.875646
C	3.842233	−3.186776	−3.202655
C	1.711281	−3.730205	−2.003075
H	1.383491	−4.281187	−2.879333
C	0.859007	−3.706887	−0.911240
N	1.106779	−3.050969	0.273942
C	−0.017909	−3.237069	1.047666
C	−0.976910	−4.054396	0.345496
C	−0.433050	−4.346841	−0.866368
C	−0.219334	−2.695453	2.306958
H	−1.180100	−2.859041	2.782652
C	0.698111	−1.909165	2.999276
C	0.466657	−1.372546	4.319170
C	2.497209	−0.790999	3.540913
N	1.935946	−1.545030	2.541450
C	1.591370	−0.686407	4.659910
C	3.749991	−0.197070	3.484362
H	4.075861	0.361818	4.356518
C	4.617665	−0.246310	2.399144
C	5.916421	0.380983	2.367732
N	4.369850	−0.892412	1.217960
C	5.477443	−0.690489	0.440427
C	5.658033	−1.190009	−0.845222
H	6.590891	−0.948240	−1.345655
Fe	2.673223	−1.837559	0.689799
C	6.451819	0.105012	1.147009
O	1.950769	−0.514550	0.076160
C	−4.577204	1.590705	1.510182
C	−3.409159	2.362069	0.837664
C	−3.165459	3.698023	1.590228
H	−2.383047	4.299929	1.118446
H	−4.077909	4.301923	1.625057
H	−2.851851	3.509951	2.622528
H	−5.430175	2.262007	1.657079
H	−4.914012	0.802796	0.822338
C	−4.210772	0.936216	2.847105
H	−5.042134	0.348905	3.250200
H	−3.972160	1.700332	3.601525
C	−2.995427	0.034443	2.712978
O	−2.848776	−0.962464	3.417649
C	−1.987525	0.463745	1.736195
C	−2.125469	1.529932	0.916560
C	−0.967050	1.962276	0.050960
H	−0.148634	1.238097	0.124465
H	−0.577237	2.916153	0.440511
C	−1.381108	2.170728	−1.413036
H	−1.643476	1.199381	−1.857335
H	−0.524925	2.553978	−1.980660
C	−2.578470	3.123340	−1.533980
H	−2.266278	4.109692	−1.158605
C	−3.769921	2.598775	−0.679827
H	−3.993942	1.596110	−1.081429
C	−5.050905	3.447646	−0.876756
H	−4.924568	4.427868	−0.401922
H	−5.893463	2.970782	−0.363788
C	−5.430482	3.649520	−2.358747
H	−6.311202	4.300563	−2.432978
H	−5.721187	2.681018	−2.791734
C	−4.256125	4.219394	−3.166224
C	−3.971738	5.678477	−2.749857
H	−4.852614	6.298727	−2.954756
H	−3.751122	5.775444	−1.683668
H	−3.122272	6.111480	−3.290743
C	−3.044138	3.274932	−2.988368
H	−3.400811	2.276575	−3.296597
C	−2.042675	3.726335	−4.074428
H	−1.367929	2.918631	−4.374129
H	−1.410824	4.545264	−3.708945
C	−2.952112	4.202699	−5.245298
H	−2.841134	3.586080	−6.142639
H	−2.707773	5.229753	−5.546384
C	−4.413722	4.152192	−4.709285
H	−4.863218	3.184070	−4.961684
O	−5.291613	5.100709	−5.295802
H	−4.875239	5.973783	−5.223142
H	2.719150	−4.351049	2.444268
H	7.414343	0.399172	0.748507
H	3.624805	−3.718039	−4.120392
H	6.346907	0.949675	3.182005
H	5.844709	−2.303311	−3.468871
H	−0.854262	−4.936756	−1.670384
H	1.802034	−0.145305	5.573609
H	−0.452225	−1.502299	4.874426
H	−1.937652	−4.351636	0.745615

H	-1.061942	-0.104607	1.728533

TS_6α

S	49.026537	42.506710	44.557039
N	47.442577	43.020274	42.124579
C	47.946792	43.947177	41.252207
C	47.308687	41.852017	41.416207
C	48.151440	43.346889	39.956633
C	47.762165	42.046735	40.060605
C	46.772171	40.665028	41.893051
H	46.728361	39.826102	41.205849
C	46.254613	40.477109	43.166007
N	46.230712	41.415898	44.161309
C	45.620950	40.829748	45.236941
C	45.239425	39.475038	44.909448
C	45.639411	39.254090	43.628471
C	45.436848	41.422517	46.477812
H	44.936187	40.839373	47.243725
C	45.866325	42.695700	46.825688
C	45.717833	43.268567	48.139918
C	46.749228	44.708303	46.750110
N	46.504134	43.588252	45.996130
C	46.273923	44.510792	48.096149
C	47.325316	45.881685	46.282723
H	47.474833	46.687094	46.994713
C	47.700105	46.108764	44.967333
C	48.272994	47.340530	44.486411
N	47.573349	45.212775	43.930634
C	48.081798	45.840361	42.816696
C	48.234084	45.269776	41.561991
H	48.647100	45.892269	40.774637
Fe	46.891011	43.335762	44.040628
C	48.500988	47.177487	43.153837
O	45.261313	43.669022	43.563900
C	40.589980	46.550764	44.869968
C	41.981143	47.000569	44.350935
C	42.523038	48.158097	45.231654
H	43.490153	48.526686	44.875546
H	41.825872	49.002233	45.247684
H	42.665116	47.824570	46.264617
H	39.936494	47.423382	44.977548
H	40.130039	45.904865	44.109451
C	40.633705	45.783639	46.195905
H	39.641554	45.420636	46.482947

H	40.973342	46.437424	47.012473
C	41.584560	44.598793	46.135487
O	41.422394	43.599600	46.824417
C	42.748099	44.745328	45.244662
C	42.972917	45.824072	44.462775
C	44.246240	45.966012	43.692887
H	44.824000	44.825556	43.662012
H	44.999625	46.548976	44.236574
C	44.090432	46.393444	42.248861
H	43.607129	45.585675	41.681257
H	45.075663	46.559022	41.800773
C	43.217714	47.663074	42.139948
H	43.750159	48.486684	42.636741
C	41.849662	47.425240	42.840282
H	41.407466	46.553491	42.329658
C	40.870306	48.607060	42.621093
H	41.199234	49.476110	43.203007
H	39.880686	48.341681	43.008270
C	40.722711	49.016324	41.140551
H	40.062422	49.889438	41.060217
H	40.231373	48.201969	40.588151
C	42.086569	49.298466	40.496061
C	42.706969	50.584596	41.082121
H	42.055017	51.439246	40.865481
H	42.824656	50.537554	42.167849
H	43.696650	50.802024	40.663894
C	42.972278	48.042680	40.673843
H	42.394408	47.208882	40.239107
C	44.169520	48.277042	39.726864
H	44.627413	47.337706	39.401908
H	44.956225	48.856115	40.226178
C	43.554623	49.077170	38.540799
H	43.591597	48.525398	37.596500
H	44.098284	50.015449	38.370145
C	42.084548	49.394610	38.945779
H	41.420993	48.611489	38.559436
O	41.563102	50.591015	38.389865
H	42.190692	51.305607	38.580641
H	48.776529	42.159711	45.838977
H	48.933845	47.879520	42.452470
H	47.764911	41.278049	39.298398
H	48.474863	48.205904	45.104882
H	48.545529	43.867260	39.092980
H	45.533018	38.356612	43.032622
H	46.350336	45.244233	48.888683
H	45.241519	42.770672	48.974531
H	44.738368	38.796934	45.588280

H	43.463398	43.928299	45.263866

RC_2 β

S	2.813590	−3.916042	2.798914
N	2.256147	−3.395844	−0.148859
C	3.321224	−3.558722	−1.000343
C	1.310922	−4.302611	−0.553431
C	3.042166	−4.599394	−1.959588
C	1.791990	−5.059703	−1.683590
C	0.066673	−4.483196	0.038391
H	−0.584617	−5.237570	−0.392417
C	−0.405422	−3.790310	1.141174
N	0.278196	−2.796034	1.804777
C	−0.576213	−2.344632	2.786507
C	−1.808048	−3.094620	2.759437
C	−1.701440	−3.990666	1.741794
C	−0.308715	−1.303004	3.659726
H	−1.093145	−1.012082	4.349122
C	0.876171	−0.574102	3.704645
C	1.134186	0.494672	4.639097
C	2.900693	0.141421	3.290641
N	1.959958	−0.774941	2.892532
C	2.398005	0.932001	4.388678
C	4.152232	0.302054	2.713969
H	4.803903	1.058237	3.141363
C	4.636385	−0.413513	1.624482
C	5.942407	−0.222481	1.042387
N	3.952481	−1.391953	0.954752
C	4.791351	−1.824655	−0.036006
C	4.501241	−2.831536	−0.950627
H	5.264398	−3.070500	−1.685179
Fe	2.056920	−1.983246	1.283272
C	6.038525	−1.100851	0.006903
O	1.384367	−0.883375	0.288506
C	−0.974963	2.881420	0.174058
C	−1.862917	2.106479	−0.837084
C	−1.000254	1.057947	−1.590835
H	−1.582718	0.500983	−2.331943
H	−0.171332	1.543105	−2.116037
H	−0.561859	0.332172	−0.899146
H	−0.053383	3.206766	−0.319869
H	−1.507900	3.793489	0.478582
C	−0.615629	2.084047	1.433454
H	−0.041854	2.689830	2.142333
H	0.011507	1.217445	1.177077
C	−1.853829	1.557731	2.131279
O	−1.910963	1.389708	3.347030
C	−2.971072	1.185743	1.250023
C	−2.976773	1.368317	−0.088331
C	−4.105568	0.811155	−0.921286
H	−4.874267	0.381998	−0.268680
H	−3.715514	−0.018766	−1.530113
C	−4.712340	1.862152	−1.862435
H	−5.226722	2.630260	−1.266191
H	−5.474549	1.391029	−2.495212
C	−3.635258	2.532442	−2.726910
H	−3.191828	1.760936	−3.373908
C	−2.527574	3.146104	−1.821473
H	−3.051809	3.866668	−1.170797
C	−1.492326	3.962207	−2.635132
H	−0.862498	3.283994	−3.222241
H	−0.815033	4.484401	−1.950621
C	−2.133231	5.001890	−3.578258
H	−1.352949	5.505316	−4.163713
H	−2.623732	5.781745	−2.977130
C	−3.179139	4.356854	−4.497990
C	−2.496612	3.428288	−5.525202
H	−1.813072	4.012549	−6.152980
H	−1.903696	2.641895	−5.051247
H	−3.216483	2.930050	−6.185085
C	−4.224216	3.639732	−3.611205
H	−4.601585	4.411749	−2.917983
C	−5.389965	3.319665	−4.573203
H	−6.346625	3.233611	−4.048346
H	−5.224134	2.363668	−5.085310
C	−5.375378	4.503906	−5.584158
H	−6.281592	5.115185	−5.530043
H	−5.309672	4.143105	−6.618793
C	−4.110348	5.347890	−5.246970
H	−4.380877	6.150630	−4.550288
O	−3.544861	6.031939	−6.354326
H	−3.397096	5.382291	−7.059351
H	2.264621	−3.470891	3.951654
H	6.868252	−1.260450	−0.669720
H	1.234505	−5.841759	−2.182932
H	6.676363	0.491180	1.394131
H	3.726043	−4.923466	−2.733586
H	−2.424663	−4.724970	1.410752
H	2.949993	1.721656	4.882237
H	0.418962	0.856375	5.364885
H	−2.636675	−2.936650	3.437563

H	-3.802278	0.682763	1.741176

TS_2 β

S	48.527686	41.842938	45.083176
N	47.186855	42.152745	42.427020
C	48.051966	42.156576	41.363675
C	46.335604	41.104628	42.215828
C	47.738735	41.076067	40.456176
C	46.667339	40.425164	40.983297
C	45.309777	40.724853	43.075578
H	44.707946	39.870112	42.781100
C	45.003036	41.321860	44.289422
N	45.640349	42.418408	44.825271
C	44.994301	42.687111	46.007950
C	43.945183	41.724386	46.237194
C	43.948666	40.880351	45.170662
C	45.287747	43.752969	46.844916
H	44.683313	43.866078	47.738400
C	46.280096	44.703332	46.622781
C	46.580285	45.791842	47.519663
C	47.940480	45.810722	45.727661
N	47.109143	44.733801	45.532545
C	47.613509	46.481409	46.963112
C	48.967603	46.193340	44.874712
H	49.546398	47.067429	45.158911
C	49.329439	45.549750	43.694911
C	50.411907	45.965050	42.836476
N	48.718458	44.435246	43.189543
C	49.381863	44.134257	42.032297
C	49.079968	43.071035	41.183430
H	49.698234	42.955682	40.297921
Fe	47.105579	43.496556	43.954585
C	50.445581	45.081199	41.800608
O	46.003937	44.412555	42.968716
C	44.514823	47.229101	42.540123
C	43.523432	46.713094	41.457001
C	44.227064	45.642177	40.578925
H	43.567921	45.253847	39.795815
H	45.113205	46.062490	40.092600
H	44.567271	44.806814	41.196377
H	45.506997	47.367367	42.096320
H	44.185646	48.232006	42.861713
C	44.639543	46.380887	43.790095
H	45.311024	46.789995	44.547394
H	45.338503	45.270993	43.448628
C	43.370343	45.875957	44.362330
O	43.246498	45.579834	45.554279
C	42.253274	45.720564	43.415185
C	42.301721	46.062407	42.111058
C	41.123362	45.773177	41.211683
H	40.288870	45.384281	41.806027
H	41.400513	44.975856	40.505678
C	40.690325	47.007717	40.406777
H	40.281326	47.764032	41.092957
H	39.879708	46.732739	39.720675
C	41.867687	47.614712	39.630694
H	42.207453	46.870256	38.895382
C	43.035937	47.948787	40.603429
H	42.610298	48.657302	41.334456
C	44.193865	48.693016	39.893166
H	44.726153	48.003726	39.227878
H	44.929998	49.022480	40.634857
C	43.728745	49.920869	39.082527
H	44.584081	50.370245	38.561416
H	43.346840	50.687183	39.773234
C	42.615885	49.550230	38.092286
C	43.173639	48.659746	36.961202
H	43.947091	49.206778	36.408738
H	43.635424	47.743504	37.337750
H	42.400782	48.355532	36.245643
C	41.461846	48.894039	38.886480
H	41.188699	49.624993	39.667530
C	40.279202	48.865485	37.892947
H	39.311171	48.858996	38.403728
H	40.312759	47.963310	37.269570
C	40.484058	50.146567	37.031740
H	39.676076	50.873667	37.159441
H	40.514059	49.905781	35.961174
C	41.852865	50.749149	37.467233
H	41.690596	51.495032	38.254809
O	42.531903	51.468219	36.449391
H	42.593459	50.892310	35.671272
H	48.105518	42.066196	46.348472
H	51.119425	45.058757	40.953670
H	46.144500	39.560440	40.594982
H	51.052497	46.818753	43.017316
H	48.278407	40.859891	39.542981
H	43.302417	40.033293	44.979131
H	48.122082	47.358562	47.342444
H	46.056966	45.988104	48.446293
H	43.292281	41.720517	47.100257

H	41.354786	45.271683	43.834506

RC_2α

S	3.665597	−3.382479	1.554415
N	1.386669	−2.329822	−0.178954
C	1.615709	−2.263367	−1.531881
C	0.327533	−3.185072	−0.019334
C	0.685063	−3.108506	−2.239843
C	−0.115143	−3.681095	−1.300358
C	−0.249927	−3.529591	1.197087
H	−1.090970	−4.215810	1.172057
C	0.167281	−3.080601	2.439313
N	1.206834	−2.204160	2.657448
C	1.246336	−2.001390	4.019237
C	0.228621	−2.788796	4.671129
C	−0.439944	−3.456181	3.692559
C	2.128605	−1.155984	4.672494
H	2.044524	−1.087510	5.752948
C	3.121248	−0.395236	4.061783
C	4.050866	0.449386	4.771273
C	4.431684	0.497519	2.555175
N	3.370932	−0.356091	2.715611
C	4.868387	1.002550	3.833613
C	5.007297	0.839224	1.337866
H	5.844879	1.529975	1.360430
C	4.592426	0.382909	0.093639
C	5.188922	0.776892	−1.160596
N	3.562115	−0.494241	−0.122282
C	3.492587	−0.662296	−1.477955
C	2.588426	−1.485750	−2.141570
H	2.632760	−1.495516	−3.224567
Fe	2.290163	−1.226934	1.251101
C	4.498435	0.132589	−2.139873
O	1.237690	0.007389	1.126304
C	−0.625530	1.859665	−1.121091
C	−1.834870	2.524348	−1.829678
C	−1.732626	4.068179	−1.699191
H	−2.591117	4.582257	−2.143017
H	−1.669448	4.367845	−0.648467
H	−0.834165	4.439435	−2.203462
H	−0.491532	2.285446	−0.122350
H	−0.843682	0.794949	−0.968323
C	0.696236	1.965944	−1.888549
H	1.484816	1.418999	−1.365583
H	1.026766	3.013493	−1.958460
C	0.565053	1.441829	−3.306019
O	1.498142	0.927220	−3.916562
C	−0.746951	1.644592	−3.943005
C	−1.823845	2.171016	−3.320496
C	−3.080839	2.466312	−4.102999
H	−2.979782	2.090496	−5.127459
H	−3.197781	3.558286	−4.183365
C	−4.337239	1.892875	−3.430927
H	−4.297761	0.794120	−3.463852
H	−5.226003	2.194163	−3.998943
C	−4.451490	2.349880	−1.969835
H	−4.569921	3.444035	−1.966356
C	−3.162444	1.965224	−1.186712
H	−3.084290	0.868769	−1.276885
C	−3.280727	2.272295	0.326990
H	−3.251598	3.356198	0.490748
H	−2.412393	1.862935	0.853523
C	−4.560390	1.698368	0.970366
H	−4.612875	1.996271	2.025506
H	−4.507469	0.599716	0.959355
C	−5.819409	2.140505	0.211675
C	−6.063217	3.652764	0.404546
H	−6.218028	3.868287	1.468619
H	−5.215743	4.259201	0.074873
H	−6.943116	4.010881	−0.142787
C	−5.663246	1.721065	−1.269023
H	−5.469494	0.634361	−1.248127
C	−7.076228	1.894129	−1.869189
H	−7.240451	1.243074	−2.733659
H	−7.231366	2.923447	−2.215790
C	−8.028566	1.554740	−0.684943
H	−8.630290	0.659763	−0.871510
H	−8.740033	2.369965	−0.500100
C	−7.118072	1.366392	0.564960
H	−6.860709	0.305848	0.674118
O	−7.735125	1.697462	1.799317
H	−8.094550	2.594852	1.719101
H	4.042516	−3.181547	2.837457
H	4.627518	0.185620	−3.212296
H	−0.931083	−4.378326	−1.441638
H	6.020283	1.463186	−1.258846
H	0.663937	−3.232925	−3.314748
H	−1.271095	−4.142627	3.791276
H	5.693033	1.689081	3.976578
H	4.064467	0.585944	5.845061
H	0.060071	−2.812588	5.740193

H	-0.793536	1.384171	-4.999121

TS_2α

S	49.482970	41.835822	45.011896
N	47.631075	41.766799	42.691667
C	48.235080	41.951850	41.474317
C	47.030017	40.533061	42.630014
C	48.028623	40.801115	40.629885
C	47.275976	39.922683	41.345238
C	46.288503	39.943173	43.645098
H	45.864226	38.964542	43.441081
C	46.063064	40.482247	44.905342
N	46.521071	41.700992	45.330040
C	46.076715	41.841682	46.622425
C	45.331355	40.671392	47.026607
C	45.319609	39.829085	45.959203
C	46.307265	42.951254	47.421197
H	45.898486	42.933647	48.427306
C	47.012969	44.092568	47.043989
C	47.225154	45.235050	47.898353
C	48.158415	45.543702	45.875931
N	47.595974	44.295899	45.821838
C	47.939539	46.138821	47.170964
C	48.835508	46.160895	44.829109
H	49.239419	47.151541	45.016974
C	49.047235	45.619661	43.567366
C	49.793993	46.270081	42.516705
N	48.612854	44.385146	43.152922
C	49.091325	44.231451	41.879932
C	48.918627	43.097126	41.093750
H	49.335883	43.118097	40.092479
Fe	47.518081	43.081677	44.210033
C	49.822396	45.404459	41.466880
O	45.965576	43.682806	43.737255
C	44.266199	45.959147	42.683476
C	43.173187	46.556256	41.747168
C	43.634794	47.939660	41.218289
H	42.884318	48.407861	40.572311
H	43.843879	48.628308	42.043194
H	44.554314	47.832822	40.633154
H	44.558930	46.697936	43.435556
H	43.807963	45.125345	43.232866
C	45.508812	45.431165	41.990310
H	45.890942	44.440852	42.828944
H	46.407026	46.050769	42.026077
C	45.317322	44.768703	40.676513
O	46.247910	44.282128	40.030294
C	43.948555	44.786509	40.123296
C	42.983902	45.633393	40.538347
C	41.715630	45.787844	39.732657
H	41.650398	44.986559	38.987765
H	41.778558	46.730460	39.165423
C	40.453612	45.836399	40.601925
H	40.295672	44.857280	41.077615
H	39.579103	46.023980	39.966915
C	40.569894	46.912812	41.689544
H	40.668570	47.889223	41.190717
C	41.828637	46.646457	42.565925
H	41.678143	45.636951	42.983334
C	41.914397	47.612005	43.774941
H	42.165826	48.621264	43.428206
H	42.734234	47.305845	44.433563
C	40.615708	47.674298	44.605448
H	40.723194	48.412190	45.411065
H	40.451748	46.702058	45.092967
C	39.401200	47.996778	43.724175
C	39.476536	49.450233	43.209255
H	39.471500	50.143373	44.059062
H	40.388551	49.646751	42.639745
H	38.633238	49.709834	42.558599
C	39.331653	46.941492	42.595554
H	39.310694	45.963933	43.108116
C	37.928894	47.131902	41.976891
H	37.559444	46.217478	41.502239
H	37.945065	47.906157	41.199767
C	37.045598	47.569417	43.182334
H	36.265589	46.839363	43.419760
H	36.528121	48.514849	42.974051
C	38.016201	47.762236	44.385296
H	38.070692	46.832576	44.964637
O	37.596727	48.732063	45.332694
H	37.406770	49.552771	44.851774
H	49.237843	41.929892	46.338088
H	50.291046	45.529323	40.499390
H	46.911112	38.947921	41.047748
H	50.240672	47.253162	42.594346
H	48.405705	40.703155	39.620264
H	44.860518	38.852265	45.875571
H	48.289724	47.119122	47.468199
H	46.867288	45.319794	48.916555
H	44.882199	40.532035	48.001719

H	43.807364	44.185122	39.227067

RC_15 β

S	3.732997	−2.402046	1.758955
N	0.993339	−3.219046	0.743770
C	0.991711	−4.037033	−0.359860
C	0.369701	−3.928861	1.736908
C	0.360926	−5.297435	−0.049302
C	−0.030563	−5.227964	1.251103
C	0.162629	−3.480359	3.035403
H	−0.352800	−4.149509	3.717355
C	0.583919	−2.260099	3.538190
N	1.239770	−1.288282	2.816059
C	1.433384	−0.239226	3.689204
C	0.917803	−0.574655	4.993891
C	0.388307	−1.823718	4.899062
C	2.014571	0.973505	3.359069
H	2.097167	1.716707	4.146295
C	2.497919	1.322218	2.100548
C	3.104575	2.592945	1.786643
C	3.052988	1.245799	−0.014177
N	2.479312	0.514949	0.994885
C	3.450040	2.545454	0.470023
C	3.237957	0.803738	−1.317720
H	3.714258	1.488513	−2.012717
C	2.867553	−0.443411	−1.805122
C	3.092959	−0.887089	−3.159524
N	2.256795	−1.428570	−1.074602
C	2.091621	−2.482268	−1.931120
C	1.508096	−3.702129	−1.601938
H	1.442512	−4.449561	−2.386641
Fe	1.610001	−1.297449	0.825200
C	2.611639	−2.157799	−3.237386
O	0.150887	−0.690317	0.436257
C	−5.459318	−0.023120	−1.103989
C	−3.907036	−0.022186	−1.054159
C	−3.422790	−0.686638	0.263866
H	−2.334725	−0.658878	0.371136
H	−3.862956	−0.188816	1.134491
H	−3.727504	−1.738491	0.296876
H	−5.859923	0.374365	−0.165161
H	−5.782520	0.664159	−1.898340
C	−6.077985	−1.398174	−1.377649
H	−7.167125	−1.339452	−1.473656
H	−5.873816	−2.088026	−0.545694
C	−5.512347	−2.028355	−2.640472
O	−6.162561	−2.802447	−3.332835
C	−4.111944	−1.697988	−2.944237
C	−3.353849	−0.839576	−2.226627
C	−1.886887	−0.681527	−2.538861
H	−1.636722	−1.250356	−3.442053
H	−1.297902	−1.111718	−1.715660
C	−1.478606	0.791411	−2.686994
H	−1.962494	1.222927	−3.576486
H	−0.395866	0.850522	−2.850171
C	−1.873871	1.598022	−1.443286
H	−1.316144	1.183287	−0.593129
C	−3.403258	1.467866	−1.182585
H	−3.889085	1.859508	−2.092750
C	−3.870496	2.368520	−0.011441
H	−3.496721	1.965203	0.936795
H	−4.963702	2.346789	0.060591
C	−3.418075	3.837465	−0.147686
H	−3.731761	4.408231	0.736265
H	−3.926369	4.293908	−1.010018
C	−1.900978	3.939698	−0.357724
C	−1.147401	3.529542	0.926471
H	−1.433296	4.195073	1.750732
H	−1.377834	2.508524	1.239687
H	−0.058553	3.582212	0.810553
C	−1.528166	3.085709	−1.592453
H	−2.158697	3.466333	−2.415496
C	−0.080920	3.509131	−1.928874
H	0.154997	3.358542	−2.987399
H	0.642125	2.917194	−1.355256
C	−0.024405	5.010480	−1.520048
H	0.146494	5.675350	−2.372824
H	0.794199	5.199541	−0.813221
C	−1.385506	5.324795	−0.830849
H	−2.090958	5.715383	−1.574228
O	−1.324681	6.347130	0.153136
H	−0.645253	6.092566	0.796953
H	4.201247	−1.345240	2.460777
H	2.603087	−2.826579	−4.088376
H	−0.537893	−5.977514	1.844631
H	3.564048	−0.294144	−3.932874
H	0.239983	−6.114622	−0.748627
H	−0.094512	−2.411608	5.669031
H	3.934037	3.305703	−0.129510
H	3.246177	3.400334	2.493658
H	0.958188	0.077059	5.857220

H	-3.681550	-2.242407	-3.782970

TS_15 β

S	49.416013	41.880188	44.629078
N	46.844406	41.469710	43.017004
C	47.078943	41.093187	41.721081
C	46.051958	40.489995	43.560414
C	46.413422	39.845163	41.432461
C	45.775501	39.470856	42.574828
C	45.572170	40.462083	44.863083
H	44.946101	39.619225	45.140493
C	45.827850	41.411304	45.844581
N	46.610809	42.522965	45.685611
C	46.589342	43.181393	46.887509
C	45.773086	42.459312	47.836844
C	45.300133	41.360588	47.189491
C	47.261897	44.365064	47.163383
H	47.166996	44.770704	48.166118
C	48.040619	45.075305	46.259166
C	48.727739	46.309286	46.564505
C	49.074404	45.688137	44.429690
N	48.271296	44.716655	44.956574
C	49.369321	46.691505	45.427187
C	49.545281	45.714920	43.120903
H	50.192456	46.541200	42.842404
C	49.281169	44.762520	42.146440
C	49.824085	44.801638	40.810400
N	48.472403	43.659644	42.299298
C	48.513624	42.994153	41.095381
C	47.855264	41.804954	40.814388
H	47.972486	41.389918	39.818061
Fe	47.519717	43.115866	43.965016
C	49.348704	43.705306	40.159067
O	46.042051	43.994682	43.716207
C	39.055818	44.090725	42.172297
C	40.463766	43.653210	42.659377
C	40.504843	43.656513	44.211422
H	41.497039	43.407269	44.599461
H	40.226599	44.637242	44.610393
H	39.801866	42.921962	44.618128
H	38.753329	45.006932	42.690744
H	39.120869	44.342211	41.104557
C	37.969659	43.024070	42.349306
H	37.012138	43.350684	41.931202
H	37.793107	42.822077	43.415976
C	38.363540	41.709554	41.695319
O	37.537516	40.933049	41.233607
C	39.802796	41.398545	41.708891
C	40.760036	42.227840	42.177446
C	42.189864	41.754609	42.277767
H	42.288450	40.764235	41.819789
H	42.451764	41.632723	43.340078
C	43.183060	42.744944	41.653489
H	43.027306	42.780496	40.564634
H	44.203901	42.388964	41.826167
C	43.001536	44.149000	42.242538
H	43.242659	44.099440	43.311925
C	41.535604	44.632104	42.041127
H	41.364372	44.595397	40.951675
C	41.331306	46.108354	42.467751
H	41.363030	46.187615	43.560257
H	40.331034	46.441176	42.170123
C	42.367631	47.080059	41.862658
H	42.202592	48.090916	42.256731
H	42.216284	47.140450	40.774648
C	43.803095	46.611638	42.142527
C	44.129121	46.731893	43.646862
H	44.093834	47.787199	43.945295
H	43.404773	46.201641	44.269372
H	45.110255	46.321580	43.904020
C	43.941194	45.172502	41.590417
H	43.623473	45.230725	40.527124
C	45.437155	44.932109	41.495574
H	45.773813	44.115534	40.853635
H	45.836234	44.450406	42.648982
C	46.089290	46.295145	41.307115
H	46.687903	46.344272	40.390283
H	46.785501	46.523682	42.128085
C	44.913773	47.319327	41.316026
H	44.547813	47.450731	40.289915
O	45.277395	48.624112	41.725180
H	45.664977	48.560319	42.612459
H	49.280596	42.001616	45.967841
H	49.543111	43.386797	39.142838
H	45.175274	38.587923	42.754136
H	50.489104	45.571082	40.439369
H	46.446113	39.334625	40.478437
H	44.656057	40.577891	47.569722
H	49.990313	47.562737	45.261702
H	48.709793	46.802476	47.528093
H	45.598028	42.768242	48.859550

H	40.062456	40.403200	41.352992

RC_15 α

S	−4.459210	−0.864408	0.839717
N	−3.002141	0.384488	−1.502433
C	−3.135359	−0.293368	−2.688840
C	−3.536067	1.628972	−1.718593
C	−3.780345	0.541244	−3.673259
C	−4.022709	1.737746	−3.072712
C	−3.620644	2.641594	−0.771274
H	−4.060891	3.582719	−1.086284
C	−3.219833	2.538332	0.551252
N	−2.640817	1.426012	1.119478
C	−2.383053	1.765712	2.429962
C	−2.837299	3.108475	2.695905
C	−3.351097	3.588240	1.531420
C	−1.756962	0.946656	3.355178
H	−1.613238	1.344488	4.355235
C	−1.289871	−0.341725	3.109208
C	−0.640710	−1.172740	4.093309
C	−0.791870	−2.223620	2.109748
N	−1.379770	−1.000217	1.911575
C	−0.329762	−2.343521	3.471122
C	−0.651660	−3.213174	1.144685
H	−0.141725	−4.128026	1.428332
C	−1.098776	−3.121240	−0.167347
C	−0.959439	−4.176403	−1.143166
N	−1.750130	−2.045263	−0.709330
C	−2.037195	−2.395470	−2.000483
C	−2.695477	−1.588406	−2.922145
H	−2.865685	−2.001063	−3.911995
Fe	−2.054675	−0.260550	0.161462
C	−1.552559	−3.726330	−2.282310
O	−0.610088	0.353005	−0.272591
C	3.457031	−2.079565	1.335117
C	3.744347	−1.318445	0.011463
C	5.276981	−1.263788	−0.228944
H	5.535805	−0.704014	−1.132548
H	5.787278	−0.788686	0.614821
H	5.689505	−2.272755	−0.340684
H	4.135784	−1.725634	2.118626
H	2.439971	−1.832125	1.666835
C	3.563618	−3.605946	1.221509
H	3.307886	−4.100216	2.164224
H	4.591529	−3.906752	0.970999
C	2.647683	−4.125238	0.128297
O	2.021476	−5.177441	0.236957
C	2.584128	−3.294785	−1.080346
C	3.098488	−2.048106	−1.170149
C	3.044489	−1.300592	−2.479291
H	2.504005	−1.895123	−3.224388
H	4.070017	−1.177929	−2.861020
C	2.407760	0.089656	−2.322278
H	1.347413	−0.020850	−2.059267
H	2.450419	0.617967	−3.282906
C	3.109763	0.908479	−1.229302
H	4.148553	1.088780	−1.548003
C	3.104221	0.120857	0.113521
H	2.040639	−0.051200	0.339792
C	3.678259	0.958350	1.282890
H	4.760403	1.088131	1.157170
H	3.546501	0.416354	2.226158
C	3.012790	2.344173	1.419217
H	3.490683	2.909468	2.230392
H	1.960078	2.208515	1.705521
C	3.068260	3.124867	0.098642
C	4.519293	3.546160	−0.219004
H	4.897950	4.199938	0.576298
H	5.199007	2.692359	−0.283297
H	4.601229	4.089023	−1.168045
C	2.413630	2.258116	−1.002814
H	1.405058	2.013872	−0.633794
C	2.215146	3.229010	−2.187485
H	1.394668	2.915667	−2.840377
H	3.116047	3.281891	−2.812180
C	1.931889	4.597948	−1.501455
H	0.922179	4.968582	−1.703988
H	2.622152	5.373980	−1.857981
C	2.147794	4.372383	0.024451
H	1.191747	4.116070	0.495836
O	2.584421	5.523691	0.733952
H	3.369665	5.867493	0.279962
H	−4.285448	−0.840111	2.180599
H	−1.655742	−4.229945	−3.235062
H	−4.493851	2.619126	−3.488525
H	−0.441137	−5.107915	−0.958681
H	−4.007953	0.235387	−4.686368
H	−3.783780	4.560956	1.335785
H	0.158938	−3.218477	3.880064
H	−0.457170	−0.883512	5.120200
H	−2.757817	3.606394	3.653752

H	2.082462	-3.744206	-1.934482

TS_15α

S	48.609737	42.579585	45.212764
N	47.284761	41.636642	42.709871
C	47.956377	41.981152	41.566581
C	47.196978	40.272686	42.700154
C	48.309637	40.796898	40.814370
C	47.832046	39.735223	41.515819
C	46.606396	39.503805	43.696441
H	46.613143	38.426403	43.559978
C	46.038999	39.986064	44.867882
N	45.922246	41.312024	45.214903
C	45.297924	41.328424	46.438630
C	45.031505	39.981818	46.883884
C	45.485115	39.150520	45.906710
C	44.948257	42.479338	47.133764
H	44.464448	42.352711	48.097754
C	45.168030	43.782940	46.696471
C	44.823006	44.965781	47.448832
C	45.809921	45.496820	45.496473
N	45.753428	44.124302	45.503401
C	45.219498	46.032100	46.700273
C	46.389946	46.271379	44.498114
H	46.355655	47.349444	44.625921
C	47.037624	45.787928	43.365887
C	47.648789	46.620313	42.355943
N	47.193395	44.464650	43.053629
C	47.883752	44.434698	41.873936
C	48.248652	43.281676	41.181879
H	48.796978	43.415153	40.253948
Fe	46.458807	42.891628	44.082056
C	48.177665	45.776508	41.427721
O	45.035059	42.806424	43.064053
C	42.270003	49.460764	41.220009
C	42.217846	47.949874	40.864125
C	40.966048	47.663891	39.991532
H	40.859004	46.600116	39.758669
H	40.050994	47.989734	40.496448
H	41.027847	48.200295	39.038792
H	41.292894	49.787637	41.591758
H	42.980740	49.597714	42.046938
C	42.701944	50.366193	40.061262
H	42.786908	51.411167	40.376184
H	41.958429	50.341933	39.251099
C	44.032403	49.923925	39.473811
O	44.825694	50.713803	38.977802
C	44.279063	48.472939	39.484440
C	43.461358	47.562081	40.055460
C	43.737197	46.087179	39.894084
H	44.692307	45.942479	39.377406
H	42.962156	45.650752	39.244434
C	43.729474	45.344324	41.237300
H	44.572728	45.690700	41.848026
H	43.892790	44.273659	41.077060
C	42.419530	45.589300	41.998230
H	41.593198	45.180076	41.397747
C	42.203314	47.118534	42.205916
H	43.091848	47.458518	42.763902
C	40.976902	47.432618	43.099645
H	40.051466	47.226413	42.549361
H	40.955340	48.502839	43.332708
C	40.960116	46.644023	44.426320
H	40.037474	46.865452	44.978657
H	41.793445	46.980265	45.060271
C	41.103049	45.136111	44.182757
C	39.845657	44.577649	43.483301
H	38.971707	44.710465	44.132709
H	39.625740	45.090017	42.543649
H	39.936128	43.510730	43.249022
C	42.415299	44.906919	43.372595
H	43.205703	45.401734	43.963002
C	42.650734	43.407436	43.500316
H	43.984183	43.146403	43.442412
H	42.356917	42.795333	42.643520
C	42.064653	42.985272	44.847802
H	42.805990	42.564840	45.535434
H	41.300641	42.206019	44.711946
C	41.416918	44.273779	45.435684
H	42.155592	44.807827	46.044002
O	40.338153	44.035346	46.325304
H	39.700208	43.462231	45.871727
H	48.153142	42.558548	46.485704
H	48.718252	46.022890	40.522790
H	47.900399	38.681746	41.276165
H	47.663226	47.702583	42.370072
H	48.850120	40.799067	39.876417
H	45.458999	38.068687	45.877066
H	45.143034	47.086221	46.934767
H	44.350100	44.962597	48.422599
H	44.553284	39.724842	47.820471

H	45.167642	48.151477	38.944329

RC_1 β

S	4.025923	−2.339203	1.841326
N	2.264994	−2.879659	−0.571566
C	2.852346	−2.661628	−1.793733
C	1.923974	−4.207064	−0.550685
C	2.892959	−3.886161	−2.556088
C	2.311351	−4.844827	−1.786164
C	1.306688	−4.860975	0.508609
H	1.087931	−5.916854	0.382582
C	0.969930	−4.282115	1.721220
N	1.165498	−2.959217	2.050049
C	0.656334	−2.810257	3.321986
C	0.160774	−4.073096	3.812325
C	0.352067	−4.983208	2.820041
C	0.604146	−1.617255	4.023957
H	0.166892	−1.642511	5.017403
C	1.061633	−0.386581	3.560608
C	1.003675	0.839939	4.318764
C	1.937967	1.171461	2.299496
N	1.636649	−0.166647	2.337247
C	1.549705	1.808967	3.533841
C	2.535016	1.827042	1.230939
H	2.721402	2.891029	1.341018
C	2.914625	1.234713	0.032402
C	3.548777	1.937545	−1.056895
N	2.756941	−0.089567	−0.279971
C	3.276160	−0.242607	−1.536295
C	3.328702	−1.439610	−2.243550
H	3.773423	−1.412343	−3.233701
Fe	1.822512	−1.487561	0.823536
C	3.775348	1.016848	−2.033512
O	0.344395	−1.181183	0.217158
C	−2.234891	1.059278	1.336999
C	−3.102854	1.061785	0.049682
C	−3.133837	−0.369500	−0.554283
H	−3.653145	−0.401056	−1.517779
H	−2.119230	−0.753080	−0.694712
H	−3.656877	−1.058420	0.117900
H	−1.290051	0.544657	1.140952
H	−1.990628	2.100070	1.593649
C	−2.917977	0.415529	2.548392
H	−2.296586	0.494707	3.446280
H	−3.079548	−0.658320	2.372772
C	−4.271514	1.042268	2.832764
O	−4.738759	1.118561	3.963060
C	−5.019668	1.499811	1.649522
C	−4.538799	1.474365	0.387573
C	−5.438077	1.830087	−0.771942
H	−6.404927	2.186977	−0.399205
H	−5.645458	0.915599	−1.349201
C	−4.799116	2.862005	−1.712956
H	−4.705219	3.824140	−1.187982
H	−5.460241	3.033980	−2.571415
C	−3.411317	2.406083	−2.184538
H	−3.540625	1.485191	−2.772765
C	−2.497526	2.112564	−0.958707
H	−2.456274	3.061293	−0.396367
C	−1.043792	1.782759	−1.377919
H	−1.004458	0.792245	−1.843828
H	−0.409169	1.709275	−0.489908
C	−0.432096	2.824676	−2.337038
H	0.575734	2.507816	−2.635176
H	−0.314280	3.781511	−1.805902
C	−1.325156	3.052980	−3.564403
C	−1.327613	1.802383	−4.469668
H	−0.310266	1.606691	−4.829741
H	−1.657357	0.904623	−3.941069
H	−1.978658	1.916314	−5.344546
C	−2.732187	3.461771	−3.067208
H	−2.571062	4.340408	−2.418246
C	−3.449237	3.987260	−4.330705
H	−4.241527	4.702723	−4.088730
H	−3.923857	3.165972	−4.881918
C	−2.305827	4.633177	−5.167400
H	−2.430039	5.714071	−5.286836
H	−2.269196	4.214528	−6.181550
C	−0.979903	4.301022	−4.420345
H	−0.728779	5.121715	−3.737382
O	0.159374	4.195514	−5.261657
H	−0.052118	3.560388	−5.963661
H	3.782789	−1.993361	3.125799
H	4.236835	1.159408	−3.002122
H	2.156649	−5.891804	−2.013160
H	3.785628	2.993629	−1.056044
H	3.314356	−3.980241	−3.548683
H	0.103272	−6.036632	2.814314
H	1.685483	2.859811	3.754957
H	0.597289	0.928946	5.318025
H	−0.279952	−4.222753	4.789576

H	-6.040057	1.825188	1.845165

TS_1 β

S	48.971253	42.514525	44.490411
N	47.102561	41.577773	42.353956
C	47.670381	41.532221	41.108044
C	46.673580	40.307262	42.620475
C	47.599557	40.190828	40.571608
C	46.973492	39.430767	41.508861
C	46.069065	39.901032	43.804814
H	45.786215	38.855425	43.883368
C	45.831779	40.702006	44.913569
N	46.100432	42.048620	45.000016
C	45.697236	42.433456	46.257346
C	45.186980	41.299526	46.989077
C	45.262882	40.228740	46.152874
C	45.727839	43.737681	46.732992
H	45.370020	43.908061	47.744007
C	46.166823	44.847445	46.015281
C	46.202046	46.194010	46.534198
C	47.028397	46.102039	44.442566
N	46.653819	44.812630	44.733062
C	46.735326	46.974608	45.554864
C	47.652952	46.510226	43.269963
H	47.900440	47.564064	43.182307
C	48.010102	45.685208	42.207722
C	48.679766	46.137919	41.011055
N	47.778138	44.338466	42.145110
C	48.275374	43.925290	40.940009
C	48.231972	42.620053	40.454478
H	48.663427	42.443116	39.473603
Fe	46.824816	43.218131	43.524108
C	48.846096	45.040446	40.222154
O	45.283440	43.377805	42.701163
C	43.285707	44.688583	43.528556
C	42.408567	44.984519	42.300373
C	42.380012	43.754329	41.348713
H	41.828734	43.966307	40.427308
H	43.396469	43.448588	41.091871
H	41.891566	42.903344	41.835202
H	44.427325	44.046123	43.138473
H	43.695822	45.598876	43.978586
C	42.692564	43.757222	44.554469
H	43.345267	43.623656	45.418793
H	42.514175	42.761937	44.122420
C	41.337874	44.278362	45.044449
O	40.946513	44.094810	46.187269
C	40.526354	44.955093	44.023230
C	40.965443	45.245310	42.780175
C	40.015724	45.842676	41.769843
H	39.045642	46.035121	42.241547
H	39.836122	45.104716	40.973388
C	40.579638	47.120175	41.132977
H	40.658840	47.904811	41.899712
H	39.880675	47.489964	40.372811
C	41.961063	46.867397	40.516839
H	41.836741	46.140710	39.699897
C	42.938540	46.282673	41.578939
H	42.998179	47.047820	42.371842
C	44.365859	46.113416	41.008949
H	44.373924	45.304593	40.269868
H	45.045544	45.786568	41.799071
C	44.918651	47.400399	40.363857
H	45.912563	47.204579	39.942132
H	45.054588	48.166443	41.142507
C	43.964137	47.949119	39.294179
C	43.941351	47.017000	38.063459
H	44.944058	46.966278	37.622210
H	43.652906	45.994198	38.318436
H	43.248352	47.360700	37.286474
C	42.575225	48.152447	39.944340
H	42.749763	48.814298	40.810690
C	41.788344	48.994006	38.915300
H	40.992583	49.581812	39.383773
H	41.307176	48.351001	38.167904
C	42.878214	49.892626	38.259649
H	42.725968	50.956734	38.465545
H	42.876068	49.787913	37.167002
C	44.244280	49.402431	38.826081
H	44.509267	49.997348	39.708670
O	45.345483	49.583408	37.948788
H	45.116930	49.173280	37.099974
H	48.728947	42.867123	45.773421
H	49.307955	44.975491	39.245262
H	46.738412	38.374601	41.474601
H	48.976156	47.160738	40.816875
H	47.983732	39.890658	39.605051
H	44.966578	39.204900	46.342090
H	46.933924	48.038649	45.573621
H	45.868157	46.483665	47.522339
H	44.808806	41.338987	48.002487

H	39.503537	45.184294	44.315453

RC_1α

S	47.400606	41.498855	45.306264
N	45.860348	43.119851	43.242739
C	46.529540	43.426257	42.083020
C	44.773724	42.374704	42.863195
C	45.853627	42.848792	40.946652
C	44.762198	42.196624	41.431168
C	43.813957	41.857725	43.725078
H	43.001609	41.289757	43.281991
C	43.816729	41.993378	45.103792
N	44.760021	42.684697	45.830545
C	44.356998	42.599449	47.145037
C	43.151914	41.813445	47.247701
C	42.817049	41.439274	45.983549
C	45.005457	43.192843	48.216176
H	44.572340	43.050004	49.201590
C	46.171470	43.948740	48.136892
C	46.845164	44.526287	49.274376
C	47.939994	44.976649	47.361999
N	46.853570	44.230588	46.983094
C	47.947438	45.163167	48.792280
C	48.900545	45.486838	46.498424
H	49.712591	46.057490	46.938950
C	48.903883	45.336955	45.117283
C	49.906720	45.891999	44.239753
N	47.964000	44.650342	44.394838
C	48.346727	44.757965	43.085814
C	47.682977	44.192096	42.001544
H	48.087162	44.396342	41.015246
Fe	46.282608	43.802565	45.096507
C	49.557899	45.535814	42.972658
O	45.372245	45.144228	44.941761
C	47.309405	48.956930	42.469606
C	45.766630	48.917960	42.290716
C	45.282819	50.265755	41.691162
H	44.194942	50.304787	41.582001
H	45.584220	51.104985	42.325925
H	45.716653	50.428304	40.698192
H	47.603262	49.908772	42.925184
H	47.600611	48.167853	43.175179
C	48.101981	48.749314	41.172313
H	49.180490	48.735995	41.357768
H	47.908018	49.566417	40.461875
C	47.700764	47.447464	40.504461
O	48.510386	46.717859	39.935718
C	46.268479	47.130816	40.560584
C	45.374071	47.795780	41.325268
C	43.909701	47.440194	41.258223
H	43.765610	46.583099	40.590832
H	43.360351	48.282450	40.809526
C	43.322093	47.154545	42.649536
H	43.776153	46.242283	43.058520
H	42.245131	46.967193	42.555793
C	43.581616	48.317151	43.618353
H	43.047306	49.203985	43.242524
C	45.107419	48.617823	43.693031
H	45.567804	47.677886	44.034271
C	45.439233	49.689072	44.761151
H	45.082117	50.672834	44.431779
H	46.526030	49.782182	44.866514
C	44.840384	49.368464	46.147011
H	45.066262	50.182479	46.848611
H	45.323588	48.464659	46.544639
C	43.327773	49.121605	46.060624
C	42.583587	50.434640	45.735298
H	42.762005	51.168771	46.530598
H	42.922052	50.886857	44.799353
H	41.500130	50.292562	45.645581
C	43.080735	47.992300	45.032746
H	43.686673	47.137986	45.373449
C	41.605842	47.589896	45.252154
H	41.413180	46.556831	44.946566
H	40.930020	48.225935	44.666135
C	41.386770	47.802780	46.779026
H	41.178993	46.867435	47.308083
H	40.530328	48.462438	46.971684
C	42.687550	48.470574	47.315859
H	43.372507	47.696825	47.682071
O	42.493283	49.318898	48.439223
H	41.801093	49.957887	48.207922
H	47.336690	41.386248	46.652353
H	50.041829	45.774355	42.034914
H	44.012286	41.637079	40.886788
H	50.758224	46.474340	44.568081
H	46.190336	42.938035	39.921871
H	41.974047	40.843579	45.657570
H	48.703293	45.712385	49.338835
H	46.507118	44.442799	50.299289
H	42.640651	41.589970	48.175337

H	45.944075	46.305097	39.930427

TS_1α

S	47.713946	42.068482	45.922304
N	47.444341	43.115172	42.992503
C	48.640718	43.469951	42.414846
C	46.871090	42.200676	42.141376
C	48.833747	42.747396	41.182439
C	47.731721	41.967853	41.008925
C	45.644254	41.577370	42.325389
H	45.316832	40.893779	41.548628
C	44.812169	41.741530	43.423485
N	45.066572	42.558867	44.489802
C	44.017653	42.393856	45.356359
C	43.071600	41.441279	44.818741
C	43.561788	41.040178	43.616641
C	43.877815	43.039912	46.574414
H	42.999646	42.804326	47.167855
C	44.766203	43.975266	47.093763
C	44.587650	44.657357	48.352957
C	46.472375	45.329531	47.323922
N	45.919821	44.396715	46.487334
C	45.647679	45.500097	48.496469
C	47.652581	46.022104	47.084549
H	47.975684	46.728876	47.842767
C	48.466287	45.873389	45.969209
C	49.721029	46.562027	45.786992
N	48.227464	45.032113	44.909384
C	49.319155	45.148244	44.084307
C	49.518531	44.420524	42.918349
H	50.431010	44.606249	42.360068
Fe	46.633219	43.837516	44.675979
C	50.251588	46.110322	44.617654
O	45.601175	44.984870	43.913769
C	46.175565	46.828359	42.363498
C	44.961494	47.758493	42.446659
C	45.292850	49.059817	41.647342
H	44.493329	49.801806	41.743285
H	46.219938	49.517027	42.005334
H	45.414267	48.839458	40.582099
H	47.090391	47.261706	42.773907
H	45.954628	45.854272	43.222684
C	46.388024	46.118882	41.049149
H	47.223617	45.415688	41.090818
H	46.636860	46.850770	40.258950
C	45.131449	45.389916	40.586709
O	45.183799	44.383772	39.892940
C	43.858450	46.017356	40.970232
C	43.752099	47.095327	41.775471
C	42.403559	47.724737	42.021812
H	41.619521	47.109011	41.567393
H	42.366533	48.701279	41.513392
C	42.133108	47.946546	43.516290
H	42.060963	46.973360	44.022420
H	41.164246	48.445276	43.642169
C	43.251153	48.775573	44.161318
H	43.257994	49.763976	43.675796
C	44.629524	48.081057	43.957621
H	44.546154	47.099883	44.444526
C	45.768276	48.842310	44.680652
H	45.962922	49.797080	44.176969
H	46.698251	48.267098	44.617964
C	45.468124	49.109006	46.170895
H	46.283349	49.695744	46.614783
H	45.443149	48.150180	46.706313
C	44.119921	49.817721	46.358457
C	44.196747	51.270765	45.842810
H	44.943189	51.827922	46.421837
H	44.498390	51.327000	44.793609
H	43.240786	51.800583	45.928325
C	43.026849	48.968795	45.667398
H	43.107440	47.963420	46.114798
C	41.696890	49.563253	46.181497
H	40.881890	48.833073	46.156546
H	41.379423	50.411528	45.562306
C	42.031620	50.024817	47.630729
H	41.469783	49.471095	48.389399
H	41.784679	51.084303	47.778020
C	43.565147	49.818952	47.808373
H	43.754292	48.829646	48.242312
O	44.179875	50.717001	48.720119
H	43.935751	51.618256	48.457354
H	48.529920	41.631536	44.937053
H	51.190534	46.381867	44.152214
H	47.500703	41.298001	40.191167
H	50.134032	47.281667	46.482348
H	49.697876	42.847998	40.538254
H	43.138463	40.333010	42.914899
H	45.867906	46.176613	49.312427
H	43.755877	44.498150	49.027314
H	42.159075	41.134065	45.313562

H	42.969731	45.565483	40.534326

RC_19

S	3.864446	-2.690528	0.774904
N	2.045177	-1.577963	-1.386442
C	2.680190	-0.919591	-2.410606
C	1.386732	-2.630599	-1.967561
C	2.423248	-1.580589	-3.667140
C	1.614405	-2.640377	-3.392833
C	0.621314	-3.572307	-1.289789
H	0.151489	-4.351515	-1.881910
C	0.430117	-3.612756	0.081539
N	0.940536	-2.702696	0.981025
C	0.476316	-3.097318	2.217151
C	-0.313573	-4.297828	2.093726
C	-0.345154	-4.614532	0.771395
C	0.710198	-2.425872	3.406194
H	0.274529	-2.844238	4.308382
C	1.452930	-1.256428	3.540852
C	1.686062	-0.582213	4.795261
C	2.687124	0.492621	3.092070
N	2.073132	-0.591060	2.517200
C	2.454276	0.505672	4.515860
C	3.435019	1.442720	2.410754
H	3.871564	2.246308	2.995981
C	3.674226	1.448820	1.041800
C	4.476980	2.436213	0.361517
N	3.202263	0.520750	0.152562
C	3.685720	0.894922	-1.071628
C	3.450530	0.225004	-2.267541
H	3.900430	0.637984	-3.165248
Fe	1.945379	-0.997843	0.546938
C	4.485930	2.090013	-0.954530
O	0.591095	-0.119735	0.337309
C	-3.879472	3.587614	1.050286
C	-2.983163	2.358067	0.739355
C	-1.501259	2.691821	1.064883
H	-0.822298	1.874615	0.805423
H	-1.176938	3.587646	0.524591
H	-1.379487	2.890556	2.135421
H	-3.454149	4.482951	0.584050
H	-4.862147	3.428658	0.584620
C	-4.092709	3.846519	2.545584
H	-4.784265	4.677842	2.717434
H	-3.144766	4.122349	3.030403
C	-4.627064	2.614728	3.259037
O	-5.341604	2.692279	4.251420
C	-4.179814	1.322262	2.718687
C	-3.406056	1.177172	1.620116
C	-2.885347	-0.184853	1.235029
H	-3.324302	-0.947675	1.888763
H	-1.797766	-0.210748	1.396951
C	-3.146349	-0.511489	-0.242505
H	-4.228942	-0.609539	-0.415515
H	-2.693860	-1.481050	-0.482616
C	-2.575537	0.581728	-1.154965
H	-1.487843	0.597793	-1.005331
C	-3.179115	1.966017	-0.776712
H	-4.269246	1.855320	-0.907829
C	-2.728894	3.080989	-1.754072
H	-1.667229	3.303793	-1.596884
H	-3.268635	4.008736	-1.533263
C	-2.954456	2.724742	-3.238419
H	-2.566278	3.528159	-3.878263
H	-4.035062	2.663769	-3.435627
C	-2.310476	1.378013	-3.595879
C	-0.770303	1.487910	-3.562001
H	-0.438036	2.242285	-4.286278
H	-0.393008	1.790471	-2.582239
H	-0.275478	0.541106	-3.808028
C	-2.874530	0.305427	-2.634569
H	-3.972368	0.361617	-2.741654
C	-2.449435	-1.038343	-3.267419
H	-3.123691	-1.854684	-2.988665
H	-1.445961	-1.327371	-2.933027
C	-2.468428	-0.754711	-4.798312
H	-3.219650	-1.348061	-5.329417
H	-1.502044	-0.997374	-5.259418
C	-2.748281	0.769673	-4.954637
H	-3.826399	0.934324	-5.070401
O	-2.185951	1.355004	-6.120146
H	-1.235671	1.160459	-6.115046
H	3.712112	-2.968491	2.089483
H	4.982678	2.583276	-1.780067
H	1.210623	-3.376671	-4.075778
H	4.965440	3.273031	0.843906
H	2.821390	-1.264175	-4.622707
H	-0.842371	-5.446290	0.289151
H	2.836427	1.255552	5.196446
H	1.304563	-0.912232	5.752912
H	-0.781564	-4.813802	2.922221

H	-4.473380	0.452007	3.303134

TS_19

S	48.806961	41.976513	44.209207
N	46.992226	42.468044	41.842322
C	47.477010	43.249684	40.826554
C	46.706422	41.252830	41.280468
C	47.507972	42.503628	39.589527
C	47.029285	41.261700	39.872368
C	46.165062	40.163889	41.956598
H	45.984389	39.260821	41.381362
C	45.826505	40.140721	43.300767
N	45.978754	41.194306	44.170718
C	45.467553	40.768641	45.372361
C	45.002815	39.404565	45.263281
C	45.228635	39.014920	43.980188
C	45.407539	41.534788	46.528082
H	44.975476	41.068944	47.408830
C	45.883986	42.835562	46.672097
C	45.890847	43.562715	47.918912
C	46.835519	44.767511	46.271110
N	46.455572	43.588262	45.677662
C	46.491034	44.759758	47.671812
C	47.438036	45.830753	45.609708
H	47.694870	46.705358	46.199907
C	47.713916	45.874412	44.247396
C	48.303796	47.001423	43.566630
N	47.458659	44.859008	43.361696
C	47.889555	45.308980	42.141540
C	47.887237	44.570085	40.961410
H	48.260498	45.065398	40.069895
Fe	46.642500	43.068917	43.746346
C	48.406833	46.652163	42.254706
O	45.000066	43.505082	43.297388
C	42.158382	47.302202	44.385691
C	42.565106	45.870495	43.894547
C	44.065763	45.712586	44.132807
H	44.593157	44.497306	43.690208
H	44.697768	46.405779	43.575857
H	44.353053	45.649183	45.182781
H	42.817981	48.050373	43.935118
H	41.145892	47.506653	44.010843
C	42.157976	47.466346	45.907778
H	41.787449	48.453335	46.203687
H	43.178331	47.381715	46.308876
C	41.307774	46.406810	46.592016
O	40.760264	46.608092	47.668969
C	41.228617	45.107193	45.908220
C	41.811107	44.821683	44.722965
C	41.773336	43.419313	44.173193
H	41.142254	42.788555	44.809892
H	42.791847	43.006203	44.204437
C	41.297091	43.382795	42.713265
H	40.246878	43.707522	42.655558
H	41.331043	42.349066	42.348772
C	42.169602	44.285505	41.831705
H	43.194087	43.895480	41.874584
C	42.153650	45.744131	42.375041
H	41.096763	46.058371	42.342248
C	42.932668	46.713235	41.451371
H	44.005738	46.497899	41.510515
H	42.808311	47.744948	41.799651
C	42.486316	46.636661	39.976234
H	43.104437	47.306816	39.364513
H	41.452136	47.002262	39.891949
C	42.548398	45.197038	39.447967
C	44.014994	44.728125	39.329161
H	44.558375	45.388863	38.641984
H	44.542664	44.745699	40.285579
H	44.098854	43.704026	38.947250
C	41.688909	44.305312	40.374798
H	40.686273	44.768954	40.385559
C	41.540381	42.979434	39.596355
H	40.636382	42.432112	39.881707
H	42.389460	42.314290	39.795434
C	41.515344	43.423333	38.103943
H	40.556953	43.214316	37.618168
H	42.278328	42.895020	37.517627
C	41.822565	44.950841	38.098797
H	40.881559	45.514154	38.109691
O	42.485604	45.415044	36.931909
H	43.283514	44.875894	36.815136
H	48.655915	41.822463	45.544121
H	48.806386	47.231152	41.431890
H	46.902551	40.418829	39.205015
H	48.597532	47.927455	44.044093
H	47.856745	42.893383	38.641726
H	45.012499	38.060380	43.517657
H	46.684473	45.571955	48.360769
H	45.492721	43.188879	48.853505
H	44.563403	38.836420	46.073252

H	40.689211	44.334227	46.452470

RC_18

S	2.670302	-3.124507	2.609414
N	0.591239	-2.982894	0.397591
C	1.005447	-3.413065	-0.839663
C	-0.449999	-3.801925	0.749765
C	0.211209	-4.537994	-1.271255
C	-0.696126	-4.776181	-0.286244
C	-1.165868	-3.724680	1.938613
H	-1.973491	-4.435959	2.080969
C	-0.918682	-2.829029	2.965716
N	0.054803	-1.854450	2.951782
C	-0.078120	-1.175659	4.143961
C	-1.135105	-1.757768	4.934477
C	-1.657376	-2.778644	4.203685
C	0.676447	-0.077882	4.522589
H	0.459645	0.365793	5.489530
C	1.689064	0.506146	3.766328
C	2.468964	1.642777	4.193047
C	3.111061	0.929285	2.159186
N	2.096455	0.083229	2.529764
C	3.354326	1.905270	3.193318
C	3.813260	0.860978	0.964177
H	4.598335	1.593995	0.805325
C	3.595455	-0.068420	-0.046168
C	4.351362	-0.126840	-1.273625
N	2.652340	-1.060907	-0.017897
C	2.792136	-1.745601	-1.194051
C	2.031344	-2.845400	-1.579672
H	2.255332	-3.290854	-2.544122
Fe	1.254386	-1.357353	1.397759
C	3.851603	-1.172557	-1.988701
O	0.161545	-0.353800	0.728740
C	-4.598602	2.818166	-0.542677
C	-3.423924	1.802507	-0.562305
C	-2.594412	1.932740	0.744722
H	-1.718606	1.277745	0.751186
H	-2.249197	2.962677	0.884855
H	-3.206167	1.669139	1.614582
H	-4.222292	3.814057	-0.284871
H	-5.011520	2.892588	-1.558426
C	-5.736870	2.442788	0.412269
H	-6.571811	3.147738	0.343136
H	-5.391310	2.467800	1.456146
C	-6.260283	1.041491	0.139923
O	-7.421850	0.722157	0.363861
C	-5.266288	0.075209	-0.350992
C	-3.978470	0.375422	-0.630107
C	-3.005901	-0.719551	-0.989953
H	-3.538929	-1.672879	-1.084343
H	-2.281435	-0.836877	-0.170786
C	-2.218623	-0.401336	-2.269452
H	-2.901766	-0.390410	-3.132429
H	-1.486690	-1.197681	-2.449758
C	-1.511585	0.955339	-2.154828
H	-0.789801	0.879856	-1.330759
C	-2.547642	2.075687	-1.845969
H	-3.258172	2.057107	-2.690133
C	-1.897899	3.482322	-1.847986
H	-1.253025	3.592756	-0.968571
H	-2.673964	4.250027	-1.752427
C	-1.072653	3.775831	-3.118246
H	-0.596162	4.761467	-3.034443
H	-1.748698	3.828946	-3.984536
C	-0.027882	2.680478	-3.373429
C	1.079677	2.727529	-2.297979
H	1.578994	3.704276	-2.325251
H	0.688589	2.591400	-1.286882
H	1.841954	1.953330	-2.444305
C	-0.763964	1.321802	-3.443841
H	-1.537074	1.445427	-4.222881
C	0.283344	0.348147	-4.028734
H	-0.184719	-0.492546	-4.551181
H	0.906104	-0.079247	-3.233866
C	1.130643	1.238364	-4.984843
H	1.043063	0.936149	-6.033402
H	2.198682	1.183303	-4.736561
C	0.626486	2.697949	-4.780355
H	-0.154538	2.922404	-5.516886
O	1.609203	3.697620	-5.008615
H	2.369352	3.494590	-4.441291
H	2.878773	-2.438846	3.756128
H	4.162779	-1.538777	-2.958573
H	-1.462526	-5.539296	-0.243960
H	5.160149	0.544236	-1.532949
H	0.345024	-5.063589	-2.207969
H	-2.469796	-3.445692	4.461580
H	4.107969	2.680691	3.144619
H	2.343173	2.157527	5.136895
H	-1.430467	-1.411455	5.916549

H	-5.621922	-0.950420	-0.432980

TS_18

S	49.037069	41.922246	44.969691
N	47.506791	41.395367	42.394701
C	48.346102	41.213751	41.325229
C	46.748007	40.251972	42.480486
C	48.106826	39.929448	40.711342
C	47.112716	39.334483	41.426926
C	45.773143	39.991679	43.434228
H	45.249473	39.043474	43.354851
C	45.420023	40.821177	44.493085
N	45.985428	42.034465	44.768857
C	45.359163	42.501910	45.892836
C	44.365794	41.552522	46.343764
C	44.401409	40.508585	45.472046
C	45.634712	43.714687	46.513887
H	45.067970	43.959057	47.407278
C	46.563399	44.647768	46.071124
C	46.814022	45.921171	46.708462
C	48.100686	45.651691	44.881284
N	47.363736	44.505675	44.967718
C	47.767986	46.546601	45.967908
C	49.037964	45.931684	43.891657
H	49.559697	46.881663	43.960216
C	49.381619	45.100754	42.835436
C	50.403554	45.409649	41.863931
N	48.832026	43.866379	42.569077
C	49.506538	43.382492	41.475149
C	49.279801	42.145685	40.882023
H	49.893693	41.876754	40.027522
Fe	47.413583	42.960886	43.653950
C	50.483752	44.343075	41.022245
O	46.033920	43.742667	42.827667
C	41.354538	46.669079	42.591046
C	42.030596	45.273042	42.671544
C	42.969211	45.211411	43.907384
H	43.577284	44.304044	43.922546
H	43.660392	46.060109	43.912782
H	42.387890	45.247675	44.835073
H	42.113969	47.453900	42.674272
H	40.896875	46.778644	41.597884
C	40.268954	46.899436	43.647794
H	39.777065	47.868875	43.518126
H	40.705377	46.901056	44.657299
C	39.211081	45.808363	43.609379
O	38.046901	46.011933	43.929741
C	39.689344	44.471865	43.218984
C	40.954557	44.194380	42.835806
C	41.372972	42.766129	42.588891
H	40.502821	42.104558	42.666927
H	42.074888	42.458900	43.379268
C	42.070403	42.596882	41.231488
H	41.350922	42.797973	40.423245
H	42.395907	41.555679	41.116291
C	43.267508	43.548251	41.112401
H	44.005113	43.280300	41.878641
C	42.818477	45.023520	41.328393
H	42.093154	45.234997	40.523666
C	44.004848	45.999629	41.134983
H	44.742394	45.834573	41.928557
H	43.662541	47.035845	41.230336
C	44.698600	45.839451	39.766341
H	45.546696	46.531424	39.687054
H	43.997590	46.107860	38.963442
C	45.152034	44.390639	39.561285
C	46.367170	44.045698	40.342648
H	47.146627	44.805817	40.386840
H	46.204769	44.021191	41.817584
H	46.746616	43.028934	40.303034
C	43.933291	43.443886	39.731914
H	43.190290	43.812368	39.004153
C	44.417770	42.080239	39.199564
H	43.583974	41.449047	38.878292
H	44.957580	41.523626	39.974370
C	45.360792	42.450759	38.018566
H	44.968956	42.130651	37.048059
H	46.343309	41.974190	38.128718
C	45.523016	43.993391	38.049244
H	44.748244	44.474169	37.441310
O	46.732917	44.489537	37.531188
H	47.464416	43.988670	37.927482
H	48.503819	42.165416	46.187187
H	51.144876	44.199407	40.176897
H	46.664058	38.360847	41.275803
H	50.984667	46.322964	41.851496
H	48.643363	39.545149	39.853017
H	43.808513	39.602760	45.479071
H	48.215944	47.519451	46.125652
H	46.312899	46.273300	47.601153
H	43.734963	41.684886	47.213560

H	38.953062	43.675006	43.307349

RC_8

S	4.042434	−1.837805	2.062871
N	1.981341	−2.338165	−0.113040
C	2.458632	−2.324246	−1.401224
C	1.463891	−3.592483	0.081148
C	2.245536	−3.606088	−2.028878
C	1.623061	−4.392138	−1.109823
C	0.879698	−4.046954	1.257480
H	0.504425	−5.065558	1.266148
C	0.757611	−3.314244	2.426450
N	1.167272	−2.009552	2.591764
C	0.820735	−1.668940	3.881550
C	0.211229	−2.793783	4.548054
C	0.169154	−3.810706	3.646335
C	1.007364	−0.420904	4.451987
H	0.679299	−0.288486	5.478465
C	1.579062	0.679497	3.818919
C	1.773933	1.965509	4.443740
C	2.527555	1.964825	2.324761
N	2.047050	0.696577	2.532583
C	2.364972	2.764811	3.514186
C	3.096758	2.421023	1.144201
H	3.442186	3.450155	1.123124
C	3.262537	1.669537	−0.013293
C	3.873782	2.164248	−1.222878
N	2.886738	0.361770	−0.167874
C	3.244182	0.016378	−1.442509
C	3.051451	−1.234841	−2.020766
H	3.387413	−1.366002	−3.044825
Fe	1.887601	−0.771916	1.160057
C	3.863103	1.133948	−2.113469
O	0.409535	−0.304636	0.663945
C	−5.299377	0.926748	0.158229
C	−3.830929	0.482791	−0.083178
C	−2.993868	0.710657	1.205487
H	−1.937496	0.463932	1.066412
H	−3.059217	1.754006	1.532241
H	−3.370617	0.085325	2.022428
H	−5.315203	1.930753	0.595977
H	−5.804884	1.001296	−0.814755
C	−6.105522	−0.026974	1.046450
H	−7.150849	0.287272	1.132579
H	−5.698201	−0.043087	2.068006
C	−6.068755	−1.452715	0.519380
O	−6.988763	−2.241171	0.701834
C	−4.830281	−1.844552	−0.169893
C	−3.792641	−1.013457	−0.413086
C	−2.511080	−1.552417	−0.997761
H	−2.641016	−2.607640	−1.265234
H	−1.723170	−1.510520	−0.231538
C	−2.032488	−0.734729	−2.206233
H	−2.743016	−0.852028	−3.038663
H	−1.068567	−1.129203	−2.549230
C	−1.901250	0.751109	−1.847193
H	−1.132735	0.833027	−1.067154
C	−3.257535	1.294164	−1.309337
H	−3.981548	1.132691	−2.126400
C	−3.212476	2.822456	−1.057296
H	−2.582814	3.035242	−0.185627
H	−4.214211	3.186563	−0.802996
C	−2.690441	3.628896	−2.264944
H	−2.631235	4.694053	−2.005085
H	−3.411642	3.549331	−3.091891
C	−1.330015	3.103598	−2.743575
C	−0.233266	3.411379	−1.700793
H	−0.160022	4.496099	−1.551896
H	−0.443604	2.963855	−0.726510
H	0.753228	3.043960	−2.006511
C	−1.480010	1.596240	−3.056635
H	−2.309056	1.527236	−3.783137
C	−0.195032	1.236646	−3.835445
H	−0.343561	0.378087	−4.498394
H	0.616417	0.969985	−3.147833
C	0.143244	2.537236	−4.621855
H	0.082141	2.403895	−5.706729
H	1.166313	2.874771	−4.410145
C	−0.869067	3.615930	−4.133959
H	−1.740924	3.627840	−4.799215
O	−0.380401	4.948524	−4.186867
H	0.449042	4.975213	−3.684692
H	3.991550	−1.326604	3.313733
H	4.240271	1.112877	−3.127822
H	1.300861	−5.420750	−1.208092
H	4.263332	3.165837	−1.352513
H	2.540204	−3.853362	−3.040619
H	−0.222696	−4.811237	3.776287
H	2.670061	3.799066	3.608332
H	1.491899	2.205998	5.460726
H	−0.140223	−2.785288	5.571791

H	-4.766772	-2.897005	-0.440786

TS_8

S	48.693483	42.123419	45.075977
N	46.336961	41.269884	43.386565
C	46.683999	40.794537	42.147140
C	45.697401	40.235555	44.029826
C	46.242049	39.430429	41.997805
C	45.636527	39.082582	43.166634
C	45.156307	40.281080	45.306106
H	44.661870	39.385741	45.669320
C	45.190558	41.380480	46.152057
N	45.787622	42.580713	45.872129
C	45.581520	43.380468	46.965753
C	44.822403	42.664712	47.964544
C	44.585895	41.422430	47.462794
C	46.070929	44.669256	47.124004
H	45.839667	45.183293	48.051612
C	46.873943	45.333711	46.207053
C	47.455845	46.635669	46.428277
C	48.085890	45.797429	44.436348
N	47.270252	44.844641	44.987467
C	48.215232	46.919044	45.334652
C	48.682400	45.719965	43.185039
H	49.321899	46.543367	42.882701
C	48.521880	44.674386	42.288687
C	49.154282	44.615475	40.994147
N	47.751395	43.551965	42.487972
C	47.909791	42.776709	41.365025
C	47.389304	41.503224	41.183752
H	47.600713	41.002243	40.244478
Fe	46.680227	43.109706	44.126430
C	48.767817	43.444478	40.418099
O	45.152000	43.815834	43.678435
C	39.983847	43.490279	43.009818
C	41.518795	43.250797	42.955260
C	42.064559	43.014596	44.388369
H	43.155790	42.975029	44.404603
H	41.751779	43.826492	45.054116
H	41.676841	42.077336	44.801436
H	39.754276	44.261759	43.752343
H	39.653380	43.881461	42.037371
C	39.170805	42.229200	43.320749
H	38.094135	42.425375	43.290419
H	39.394334	41.863733	44.333521
C	39.486773	41.104325	42.348070
O	38.666724	40.244449	42.051969
C	40.862121	41.081779	41.824308
C	41.804438	42.007144	42.108384
C	43.219252	41.818191	41.627337
H	43.285145	40.927970	40.993039
H	43.865704	41.640387	42.495348
C	43.759218	43.048175	40.884291
H	43.202429	43.180732	39.940376
H	44.807202	42.886852	40.608691
C	43.609955	44.318821	41.703538
H	44.474682	44.096909	42.698211
C	42.185987	44.506739	42.276948
H	41.584340	44.670115	41.362010
C	42.057443	45.809040	43.102497
H	42.649619	45.722709	44.019840
H	41.017176	45.944910	43.416565
C	42.501029	47.053372	42.307657
H	42.400222	47.952580	42.928379
H	41.826767	47.189515	41.448495
C	43.942136	46.904525	41.801442
C	44.931610	46.997357	42.980185
H	44.792706	47.954841	43.497806
H	44.794397	46.196393	43.707968
H	45.975269	46.937804	42.654665
C	44.048703	45.590183	40.987442
H	43.287679	45.697936	40.188044
C	45.408982	45.682216	40.268328
H	45.433161	45.081824	39.353352
H	46.214036	45.318556	40.912435
C	45.562416	47.204855	39.980639
H	45.564945	47.433307	38.910198
H	46.509484	47.588223	40.380472
C	44.372190	47.910837	40.698766
H	43.538370	48.029862	39.995854
O	44.645986	49.233293	41.130920
H	45.419622	49.196012	41.715171
H	48.734939	42.862136	46.206490
H	49.045424	43.043490	39.451674
H	45.182574	38.137598	43.436111
H	49.810991	45.378669	40.596501
H	46.394675	38.828138	41.111341
H	44.053533	40.598699	47.920865
H	48.809601	47.801696	45.135628
H	47.300052	47.235500	47.315913
H	44.528000	43.073471	48.922764

H	41.106695	40.218584	41.207945

QM Atoms in the ONIOM Calculations

TES_17-OH_UP:RC

C	39.902434	42.997193	40.324838
H	38.985320	43.598198	40.403804
H	39.926476	42.306364	41.179155
O	41.026005	43.860372	40.313518
H	41.707145	43.370376	40.819318
C	44.563091	49.504150	43.027046
H	44.571998	50.083996	42.098421
H	45.464902	48.889249	43.082070
H	43.683250	48.854180	43.023747

C	44.704159	51.381624	49.074280	C	46.581600	42.674905	41.525288
H	44.520781	50.998532	48.063941	H	46.018290	41.794519	41.231488
H	45.047813	50.527783	49.669316	C	47.443443	42.534649	42.605951
C	43.400584	51.909974	49.702604	C	47.578721	41.339611	43.404651
H	43.134577	52.885785	49.292107	C	48.896750	42.976775	44.221867
H	42.584756	51.218855	49.471797	N	48.259431	43.517125	43.123105
C	43.487527	52.005092	51.221827	C	48.464021	41.608060	44.394048
O	44.166758	52.896460	51.795370	C	49.807914	43.646705	45.028661
O	42.876529	51.150331	51.934252	H	50.238415	43.095824	45.857647
C	48.686651	49.109647	46.765945	C	50.229743	44.959750	44.870575
H	49.549237	48.439558	46.825039	C	51.150454	45.646131	45.754217
C	47.445569	48.250284	46.523277	N	49.837570	45.806382	43.860283
H	46.528353	48.851699	46.457523	C	50.484623	47.002946	44.076308
H	47.549303	47.690638	45.586978	C	50.330091	48.145761	43.306945
H	47.300770	47.528488	47.336193	H	50.885671	49.025277	43.607418
O	48.969068	49.992864	45.677931	Fe	48.341440	45.456552	42.513478
H	48.121880	50.402366	45.378491	C	51.323249	46.897510	45.251084
C	49.017861	44.595266	51.647772	O	47.196257	45.930692	43.574109
H	48.127997	45.182019	51.910150	C	42.954365	42.391668	45.584007
C	50.243326	45.346143	52.209544	C	43.730046	43.732620	45.673451
H	51.188809	44.874375	51.908944	C	45.260360	43.464203	45.657822
H	50.269376	46.379842	51.849505	H	45.842534	44.385015	45.738282
H	50.231337	45.393425	53.303952	H	45.543596	42.800302	46.480994
C	49.055116	44.485737	50.108359	H	45.561611	42.983872	44.724036
H	49.936848	43.909828	49.789834	H	43.308123	41.703633	46.358777
H	48.179515	43.926559	49.754896	H	41.894109	42.588744	45.794728
C	49.066765	45.849406	49.402891	C	43.066681	41.712920	44.218656
H	49.988834	46.406992	49.598953	H	42.406140	40.843348	44.141660
H	48.982934	45.727175	48.317506	H	44.084436	41.339839	44.054344
H	48.225168	46.468276	49.736389	C	42.732541	42.641399	43.075765
C	46.014558	40.795597	49.314208	O	42.419503	42.183709	41.968678
H	46.757526	40.052602	49.017132	C	42.883769	44.070534	43.307879
H	45.013949	40.444202	49.043515	H	42.638268	44.718785	42.470576
H	46.212446	41.737739	48.796728	C	43.399391	44.589708	44.448876
S	50.264264	44.977298	40.874980	C	43.745803	46.052941	44.506622
N	48.610086	47.315901	41.755897	H	43.327962	46.561350	43.630631
C	49.473433	48.282785	42.224214	H	44.839377	46.142875	44.416991
C	47.889852	47.882237	40.728095	C	43.279713	46.724966	45.804396
C	49.311125	49.488581	41.442639	H	42.180885	46.765536	45.816593
C	48.341677	49.239522	40.530144	H	43.631160	47.761904	45.826456
C	46.866416	47.268482	40.017086	C	43.761098	45.965462	47.045908
H	46.370558	47.883837	39.272979	H	44.860353	45.989938	47.055810
C	46.441031	45.951649	40.164959	C	43.278205	44.489019	46.979354
N	46.982950	45.045299	41.044120	H	42.177244	44.544958	46.922877
C	46.343242	43.849680	40.823486	C	43.617399	43.711704	48.273368
C	45.357419	44.006532	39.772710	H	44.697195	43.537973	48.324533
C	45.404904	45.305190	39.383461	H	43.152158	42.718764	48.246480

C	43.164640	44.429690	49.558847
H	43.503830	43.860631	50.429814
H	42.065016	44.447169	49.590893
C	43.688686	45.866628	49.627655
C	45.213316	45.870472	49.852557
H	45.635357	46.879484	49.823098
H	45.425309	45.461801	50.845849
H	45.748855	45.269324	49.111390
C	43.238592	46.599250	48.341313
H	42.139654	46.493763	48.309729
C	43.523143	48.085707	48.635753
H	42.874982	48.753609	48.058719
H	44.553684	48.343692	48.364820
C	43.299948	48.213983	50.171661
H	42.492899	48.904419	50.430143
H	44.195648	48.593906	50.670695
C	43.014840	46.778340	50.688807
H	41.932052	46.577360	50.619621
O	43.436957	46.534761	52.015935
H	43.583563	47.383656	52.468668
O	44.161449	49.073317	53.290025
H	43.640052	49.825099	52.930371
H	44.508028	49.415646	54.153937
H	39.844646	42.393781	39.389792
H	44.513209	50.191264	43.900324
H	45.532868	52.123590	49.024498
H	48.641989	49.668840	47.732772
H	48.902581	43.601698	52.161932
H	46.054889	40.955675	50.412818
H	50.546207	43.610769	40.855338
H	51.833074	47.668619	45.660014
H	48.001222	49.870589	39.821381
H	51.583391	45.224399	46.663381
H	49.870063	50.413370	41.582740
H	44.740170	45.774941	38.658684
H	48.703396	40.942943	45.221428
H	47.008633	40.420635	43.276589
H	44.652487	43.245657	39.420246

TES_17-OH_UP:TS_19

C	39.913238	42.984640	40.368352
H	38.962461	43.525161	40.489173
H	40.006611	42.283013	41.209700
O	40.982844	43.912303	40.333336
H	41.677710	43.512918	40.897437
C	44.615362	49.629899	42.866778
H	44.628709	50.273837	41.981994
H	45.523328	49.022626	42.886701
H	43.742847	48.974501	42.809114
C	44.694252	51.403006	49.088131
H	44.509866	51.022827	48.077021
H	45.042921	50.548267	49.680023
C	43.388913	51.919628	49.722438
H	43.113833	52.894629	49.315765
H	42.577402	51.221990	49.493285
C	43.484295	52.010833	51.241901
O	44.158004	52.908735	51.811273
O	42.888361	51.145761	51.954004
C	48.671602	49.139182	46.768413
H	49.528448	48.462057	46.827504
C	47.423515	48.289810	46.529557
H	46.513088	48.900809	46.454677
H	47.521094	47.714232	45.602259
H	47.269676	47.583256	47.354201
O	48.960331	50.020472	45.681054
H	48.115137	50.428062	45.373238
C	49.026113	44.590729	51.674608
H	48.147792	45.189463	51.948425
C	50.268254	45.323851	52.223862
H	51.204233	44.851181	51.896171
H	50.294199	46.363105	51.880343
H	50.279442	45.352745	53.318838
C	49.044484	44.481557	50.134680
H	49.885939	43.853129	49.807289
H	48.133007	43.976148	49.790490
C	49.137090	45.841449	49.427989
H	50.100165	46.331122	49.606246
H	49.027630	45.725136	48.344165
H	48.348870	46.519636	49.775709
C	45.999148	40.815341	49.332516
H	46.750379	40.087530	49.018103
H	45.001656	40.451395	49.066446
H	46.176189	41.767335	48.826341
S	50.118760	44.977238	40.938346
N	48.532524	47.224427	41.974074
C	49.408474	48.186890	42.424261
C	47.830549	47.779614	40.932203
C	49.253434	49.390456	41.632516
C	48.279435	49.139907	40.727308
C	46.847489	47.136480	40.188076
H	46.341387	47.742642	39.442984

C	46.486869	45.798032	40.289493
N	47.045758	44.887773	41.160050
C	46.426318	43.681434	40.920878
C	45.451058	43.833472	39.858266
C	45.479792	45.136733	39.484073
C	46.669049	42.515287	41.632980
H	46.122977	41.627022	41.331152
C	47.507243	42.385288	42.739067
C	47.650476	41.186924	43.533978
C	48.923495	42.844181	44.382883
N	48.281025	43.384903	43.284252
C	48.511238	41.468291	44.542981
C	49.825859	43.521171	45.193347
H	50.282568	42.963026	46.004008
C	50.221744	44.847583	45.051017
C	51.169806	45.527805	45.908601
N	49.787392	45.699576	44.067741
C	50.445402	46.889955	44.260637
C	50.289484	48.034161	43.484632
H	50.879091	48.900196	43.760051
Fe	48.353307	45.310463	42.675988
C	51.323856	46.783371	45.405752
O	47.028549	45.819360	43.727981
C	43.875588	42.690608	45.408182
C	44.314182	44.179379	45.511301
C	45.857869	44.300689	45.445894
H	46.451506	45.051052	44.382466
H	46.305511	44.878706	46.254840
H	46.369395	43.347999	45.311197
H	44.503274	42.075313	46.060502
H	42.847410	42.605188	45.786085
C	43.920050	42.136253	43.987788
H	43.553453	41.105415	43.938459
H	44.950325	42.138172	43.616851
C	43.119321	42.979135	43.027697
O	42.616061	42.480044	42.013342
C	43.078738	44.410395	43.299417
H	42.584715	45.026168	42.550759
C	43.679923	44.983324	44.369833
C	43.754176	46.480476	44.485926
H	43.201493	46.939969	43.659600
H	44.807329	46.770918	44.363622
C	43.228469	46.987301	45.837370
H	42.145097	46.805497	45.890544
H	43.364291	48.072177	45.904649
C	43.900950	46.280396	47.021070
H	44.970227	46.535489	47.013054
C	43.730168	44.747785	46.869072
H	42.641309	44.581963	46.800707
C	44.215815	43.965582	48.112432
H	45.311866	43.985941	48.152618
H	43.937936	42.910407	48.014970
C	43.637985	44.499052	49.441949
H	44.083360	43.949004	50.279538
H	42.556109	44.298142	49.468035
C	43.874800	46.004527	49.600162
C	45.372429	46.274466	49.834792
H	45.609023	47.341804	49.870931
H	45.659096	45.844658	50.800777
H	46.003587	45.825067	49.062826
C	43.292153	46.719397	48.357560
H	42.228204	46.425168	48.318750
C	43.325411	48.209735	48.740302
H	42.586916	48.800504	48.190821
H	44.303215	48.639485	48.498791
C	43.078429	48.211752	50.276205
H	42.164702	48.740915	50.558342
H	43.891866	48.717770	50.802701
C	43.043202	46.720086	50.706323
H	42.013536	46.338497	50.609471
O	43.457700	46.478558	52.036805
H	43.719279	47.317936	52.451417
O	44.167613	49.070153	53.334425
H	43.637690	49.817030	52.978191
H	44.521334	49.416315	54.194347
H	39.855237	42.390940	39.425884
H	44.552957	50.256202	43.783604
H	45.522546	52.145339	49.037288
H	48.629966	49.697004	47.736008
H	48.903621	43.596961	52.186992
H	46.047326	40.960471	50.433159
H	50.453663	43.620724	40.895956
H	51.851943	47.551977	45.796391
H	47.978625	49.750832	39.984664
H	51.649488	45.091374	46.786118
H	49.823118	50.311775	41.750901
H	44.812664	45.609560	38.762242
H	48.745381	40.811747	45.378557
H	47.100398	40.258002	43.389183
H	44.746031	43.074160	39.505217

TES_17-OH_UP:TS_6 β

C	39.734614	43.111563	40.358043	C	49.246551	49.421252	41.580406
H	38.768183	43.633084	40.390164	C	48.248682	49.178868	40.697003
H	39.792416	42.451369	41.234959	C	46.759909	47.206740	40.212274
O	40.764938	44.082272	40.349596	H	46.260835	47.816188	39.465030
H	41.526115	43.631900	40.765675	C	46.352971	45.882923	40.345038
C	44.596629	49.520277	43.013349	N	46.878932	44.977138	41.238754
H	44.596524	50.095063	42.081946	C	46.303060	43.763147	40.951082
H	45.503690	48.912814	43.063444	C	45.357595	43.910001	39.861623
H	43.718359	48.867390	43.023905	C	45.372789	45.219982	39.506831
C	44.741848	51.421908	49.037472	C	46.582999	42.578664	41.622540
H	44.565631	51.036029	48.026998	H	46.066433	41.681486	41.296273
H	45.077261	50.570603	49.638105	C	47.447568	42.444236	42.702308
C	43.437110	51.962726	49.653290	C	47.630314	41.240512	43.474755
H	43.190557	52.945751	49.249426	C	48.882448	42.910249	44.333393
H	42.611450	51.287917	49.407655	N	48.222533	43.448067	43.243497
C	43.510418	52.039368	51.173370	C	48.496391	41.525214	44.478173
O	44.178494	52.927932	51.764393	C	49.790252	43.584438	45.142642
O	42.902883	51.168909	51.868864	H	50.244039	43.022201	45.951530
C	48.809054	49.209969	46.703981	C	50.209017	44.900832	44.994061
H	49.694460	48.571761	46.762214	C	51.176676	45.569765	45.844361
C	47.609916	48.313104	46.424897	N	49.790364	45.753747	44.004931
H	46.671684	48.881771	46.385571	C	50.471034	46.934456	44.189840
H	47.731097	47.787388	45.472482	C	50.309037	48.074946	43.417365
H	47.495687	47.555596	47.208775	H	50.896382	48.944829	43.683524
O	49.072092	50.128637	45.639976	Fe	48.276075	45.369695	42.660759
H	48.211754	50.504028	45.332842	C	51.356674	46.815978	45.331427
C	48.953363	44.580922	51.658996	O	47.189864	45.904367	43.938277
H	48.045053	45.149732	51.898830	C	42.874787	42.416150	45.304102
C	50.151085	45.364922	52.234983	C	43.920166	43.549057	45.487318
H	51.111702	44.899022	51.976019	C	45.318508	42.934367	45.745150
H	50.173692	46.388728	51.847426	H	46.098645	43.696869	45.804580
H	50.104724	45.442587	53.326652	H	45.321840	42.357235	46.675380
C	49.025584	44.451965	50.121615	H	45.597710	42.257594	44.934620
H	49.927773	43.893532	49.830286	H	42.987351	41.682602	46.109034
H	48.171995	43.866419	49.757573	H	41.868829	42.846272	45.407388
C	49.021999	45.802875	49.392206	C	42.956830	41.701925	43.954143
H	49.919850	46.391785	49.608379	H	42.150019	40.970953	43.834624
H	48.979662	45.658146	48.307145	H	43.898428	41.145477	43.869362
H	48.151443	46.402628	49.683968	C	42.890333	42.679467	42.807676
C	45.953201	40.804698	49.341436	O	42.387810	42.355807	41.721932
H	46.700565	40.075124	49.022213	C	43.487785	43.985284	43.022545
H	44.953026	40.446942	49.077179	H	43.516629	44.644489	42.159969
H	46.136094	41.757500	48.837960	C	44.005481	44.404702	44.217285
S	50.123802	44.981454	40.992745	C	44.661179	45.714359	44.288961
N	48.516917	47.263405	41.936058	H	44.463534	46.313109	43.395334
C	49.410976	48.220337	42.368729	H	45.965917	45.598919	44.106157
C	47.786180	47.828177	40.917111	C	44.451014	46.525921	45.569153

H	43.497825	47.069674	45.450015
H	45.226522	47.292249	45.644586
C	44.370614	45.705455	46.871467
H	45.382965	45.368776	47.131199
C	43.454214	44.468450	46.671646
H	42.472643	44.870085	46.364752
C	43.217538	43.689299	47.988269
H	44.134185	43.165421	48.281847
H	42.463371	42.909979	47.827528
C	42.752737	44.580451	49.154832
H	42.658565	43.971564	50.058306
H	41.747172	44.969253	48.934190
C	43.701066	45.762387	49.381451
C	45.057842	45.285635	49.935533
H	45.743886	46.120940	50.107614
H	44.902716	44.802207	50.904841
H	45.558193	44.574262	49.270694
C	43.803063	46.536330	48.039364
H	42.760751	46.766751	47.759059
C	44.443957	47.881506	48.425275
H	44.199356	48.672523	47.708801
H	45.535592	47.802028	48.446818
C	43.884723	48.168536	49.847937
H	43.204694	49.023728	49.868547
H	44.686314	48.393273	50.555741
C	43.146115	46.880850	50.306117
H	42.074701	46.985924	50.065588
O	43.291757	46.581970	51.681620
H	43.419437	47.405299	52.183761
O	44.099747	49.024747	53.195458
H	43.620020	49.793619	52.815491
H	44.444030	49.371312	54.057779
H	39.757673	42.476839	39.441333
H	44.554447	50.213194	43.881706
H	45.573936	52.158481	48.991414
H	48.727139	49.739260	47.684327
H	48.852601	43.590466	52.183203
H	46.006250	40.948236	50.441500
H	50.450556	43.622690	40.943472
H	51.906832	47.575668	45.709847
H	47.929659	49.796084	39.967280
H	51.638255	45.136872	46.733633
H	49.828988	50.336243	41.685811
H	44.731752	45.691144	38.761061
H	48.730554	40.865044	45.311301
H	47.069215	40.316953	43.344364
H	44.676835	43.139888	39.484504

DHT_17 -OH_UP:RC

C	39.258440	40.791617	45.777334
H	38.650229	40.158687	45.124320
H	38.962986	41.834663	45.591708
O	40.632370	40.584754	45.469441
H	41.052212	41.441643	45.684228
C	36.894235	52.751948	39.083034
O	37.949661	53.301404	38.807081
N	35.682416	53.360227	39.152302
H	34.854636	52.817050	39.376505
C	43.242127	40.371207	40.604974
H	43.394421	39.307903	40.392847
H	44.067263	40.740823	41.219672
H	42.300444	40.490931	41.149011
C	47.525891	44.631743	38.692870
H	48.426737	44.898041	39.254046
C	46.316228	44.900714	39.588743
H	45.370060	44.665385	39.080065
H	46.370512	44.294638	40.500034
H	46.271624	45.953993	39.890890
O	47.668775	43.250900	38.352398
H	46.782679	42.815502	38.399020
C	48.156085	50.976800	39.523709
H	47.182302	50.831775	39.031268
C	49.117941	51.560202	38.466078
H	50.116863	51.747034	38.880051
H	49.244498	50.864168	37.628133
H	48.749348	52.506006	38.053053
C	48.656094	49.591683	39.978027
H	48.754588	48.991403	39.062831
H	49.674398	49.674300	40.385048
C	47.776177	48.813993	40.962713
H	46.725806	48.817119	40.646855
H	48.100170	47.768846	41.027298
H	47.822406	49.227606	41.976041
C	45.524285	50.290828	44.481241
H	46.310938	50.541224	45.199184
H	44.582843	50.156076	45.023156
H	45.776468	49.360009	43.964952
S	49.237460	41.962416	45.262116
N	47.613409	41.392344	42.724481
C	48.424316	41.385215	41.607829
C	47.046182	40.141171	42.808694

C	48.358078	40.088019	40.972417	H	43.339276	44.068951	41.468488
C	47.512385	39.331690	41.711634	C	41.854258	45.184439	40.338519
C	46.125561	39.726081	43.761837	H	40.757630	45.189662	40.241016
H	45.775984	38.702824	43.672964	H	42.249030	44.835275	39.375963
C	45.596849	40.498019	44.790220	C	42.335032	46.618993	40.614004
N	45.955941	41.795688	45.055996	H	43.433894	46.614576	40.579636
C	45.217289	42.181000	46.150393	C	41.878381	47.109242	42.024606
C	44.353716	41.099289	46.572804	H	40.774807	47.093717	42.006781
C	44.590646	40.062289	45.731894	C	42.297247	48.580974	42.261324
C	45.306577	43.422640	46.765679	H	43.388321	48.646174	42.343000
H	44.678819	43.586777	47.634007	H	41.902958	48.934061	43.220345
C	46.130409	44.467946	46.371804	C	41.814802	49.538094	41.153581
C	46.180994	45.767368	46.993252	H	42.182183	50.551658	41.354535
C	47.611159	45.679194	45.261028	H	40.717421	49.588516	41.175696
N	47.014566	44.435432	45.310711	C	42.266533	49.067239	39.762681
C	47.085019	46.511252	46.314837	C	43.790848	49.265447	39.612541
C	48.580904	46.060113	44.346181	H	44.035644	50.318946	39.779202
H	48.969268	47.067410	44.424032	H	44.361228	48.676677	40.334150
C	49.097214	45.278912	43.327618	H	44.153502	48.994211	38.613665
C	50.107231	45.717173	42.393677	C	41.813688	47.602897	39.556523
N	48.689383	43.998629	43.018516	H	40.717564	47.609252	39.670430
C	49.383239	43.641211	41.877082	C	42.084535	47.355141	38.060910
C	49.230408	42.441683	41.199178	H	41.509136	46.516554	37.656910
H	49.789086	42.315767	40.279025	H	43.143830	47.127866	37.898349
Fe	47.209347	42.944961	43.948607	C	41.683961	48.704699	37.397225
C	50.267862	44.716859	41.490097	H	40.730713	48.600810	36.874692
O	46.023367	43.536792	43.004123	H	42.396064	49.042833	36.633237
C	41.593789	46.533377	44.501643	C	41.548836	49.736904	38.563368
C	42.300319	46.131061	43.179279	H	40.488815	49.839855	38.808460
C	43.833826	46.152138	43.388457	O	41.960911	51.076314	38.279389
H	44.381764	45.961112	42.462415	H	42.823511	51.036921	37.835071
H	44.153972	47.127288	43.771349	O	40.244144	53.070100	37.012943
H	44.175963	45.398685	44.101425	H	39.402598	52.881807	37.472215
H	41.911970	47.531133	44.822616	H	40.778984	52.285621	37.263967
H	40.511328	46.594504	44.319578	H	39.001503	40.539396	46.834885
C	41.853901	45.555362	45.665747	H	36.822292	51.680435	39.355566
H	41.212170	45.772496	46.526508	H	35.558438	54.371911	39.103149
H	42.890950	45.668028	46.013606	H	43.199288	40.944787	39.652123
C	41.696071	44.086749	45.312967	H	47.504989	45.270915	37.773863
O	41.359543	43.271752	46.166151	H	47.992028	51.755024	40.319150
C	42.069570	43.658883	43.910977	H	45.412028	51.114789	43.743703
H	41.565247	42.708227	43.700216	H	49.261524	42.518001	46.540404
H	43.147747	43.436565	43.922486	H	50.966550	44.667707	40.763596
C	41.768691	44.706625	42.819319	H	47.349839	38.341266	41.627620
H	40.671508	44.803805	42.756086	H	50.662043	46.654276	42.464056
C	42.250507	44.210868	41.451167	H	48.897327	39.783965	40.077047
H	41.812960	43.223320	41.254629	H	44.034126	39.128984	45.683009

H	47.349265	47.549872	46.504431
H	45.609734	46.071022	47.863684
H	43.604604	41.136865	47.368625

DHT_17-OH_UP:TS_19

C	39.274034	40.759353	45.780351
H	38.625486	40.160526	45.132921
H	39.026644	41.816570	45.604082
O	40.632466	40.487932	45.456494
H	41.089039	41.337559	45.618870
C	36.925784	52.753997	39.045980
O	37.968944	53.313498	38.746960
N	35.711387	53.351123	39.131182
H	34.878798	52.826966	39.383106
C	43.246197	40.310231	40.567215
H	43.405024	39.248112	40.355082
H	44.063807	40.683040	41.189394
H	42.298941	40.424095	41.102783
C	47.506948	44.629073	38.645542
H	48.397704	44.902205	39.218774
C	46.283951	44.889726	39.524039
H	45.346893	44.647870	39.002149
H	46.326769	44.286493	40.437803
H	46.230397	45.944226	39.820962
O	47.661668	43.250523	38.302089
H	46.785005	42.799220	38.372859
C	48.135691	51.035356	39.507270
H	47.158041	50.881599	39.025535
C	49.076971	51.639978	38.443575
H	50.077601	51.838100	38.848059
H	49.204329	50.951441	37.599510
H	48.689857	52.583043	38.041511
C	48.659843	49.653685	39.943711
H	48.732376	49.053724	39.025889
H	49.690236	49.743829	40.317225
C	47.818982	48.873084	40.959066
H	46.757776	48.879604	40.685097
H	48.144311	47.827928	41.007913
H	47.905261	49.285153	41.970652
C	45.546333	50.312803	44.499669
H	46.332444	50.583255	45.210856
H	44.609807	50.169938	45.048014
H	45.808923	49.380545	43.991650
S	48.906475	41.897966	45.239067
N	47.436535	41.543117	42.663979
C	48.276549	41.528032	41.567279
C	46.889303	40.286337	42.752048
C	48.238922	40.219682	40.945409
C	47.376137	39.466674	41.666860
C	45.999064	39.857674	43.730047
H	45.629983	38.842194	43.630581
C	45.555746	40.598036	44.813734
N	45.951595	41.888907	45.108722
C	45.247322	42.256486	46.241260
C	44.397430	41.164825	46.666686
C	44.587611	40.151615	45.787188
C	45.340139	43.496401	46.850046
H	44.765625	43.640385	47.758336
C	46.097125	44.579468	46.405755
C	46.166229	45.864253	47.056970
C	47.489218	45.843470	45.239373
N	46.895530	44.594495	45.283655
C	47.013612	46.641963	46.342585
C	48.430155	46.236120	44.301018
H	48.836627	47.235191	44.391325
C	48.916128	45.463651	43.254212
C	49.958755	45.874845	42.344885
N	48.475402	44.202117	42.932044
C	49.200097	43.810962	41.828713
C	49.083761	42.585360	41.175120
H	49.682975	42.445842	40.282652
Fe	47.090790	43.114789	43.934969
C	50.121241	44.859210	41.454994
O	45.672022	43.582582	43.006621
C	42.299002	46.315040	44.486419
C	42.855012	45.876831	43.103749
C	44.396783	45.815856	43.137012
H	45.061342	44.564497	43.205552
H	44.882110	46.115784	42.207414
H	44.855520	46.318147	43.991607
H	42.766356	47.250770	44.811102
H	41.226003	46.524591	44.375551
C	42.492199	45.271733	45.599964
H	41.964513	45.556848	46.516306
H	43.559389	45.208033	45.847874
C	42.074298	43.866555	45.217652
O	41.558581	43.117850	46.041384
C	42.409221	43.422618	43.815692
H	41.831780	42.518612	43.589610
H	43.472487	43.140703	43.797944
C	42.158082	44.518723	42.759611

H	41.076651	44.735490	42.772036
C	42.497859	44.025001	41.352669
H	41.971667	43.078033	41.179553
H	43.571517	43.806611	41.289077
C	42.079534	45.044786	40.289070
H	40.980125	45.103914	40.266952
H	42.388550	44.698953	39.294555
C	42.641749	46.453658	40.550425
H	43.728308	46.419878	40.387438
C	42.360129	46.911874	42.012985
H	41.260914	46.919558	42.114845
C	42.828971	48.366057	42.253982
H	43.924592	48.415075	42.216128
H	42.547338	48.687863	43.262377
C	42.224888	49.361599	41.241919
H	42.604908	50.372338	41.435849
H	41.135383	49.402265	41.390603
C	42.512071	48.938815	39.792493
C	44.009414	49.130751	39.471493
H	44.297398	50.167463	39.672381
H	44.648920	48.488067	40.079686
H	44.244161	48.918256	38.421466
C	42.024554	47.483999	39.590822
H	40.948079	47.496674	39.827706
C	42.125535	47.287387	38.067224
H	41.514905	46.457559	37.698892
H	43.162096	47.076398	37.784469
C	41.649393	48.656239	37.500783
H	40.636778	48.566280	37.100509
H	42.262743	49.022132	36.665486
C	41.660091	49.648266	38.709230
H	40.641037	49.737201	39.091028
O	42.029190	50.997098	38.418643
H	42.858562	50.978410	37.913249
O	40.312543	52.997147	37.068608
H	39.451377	52.825797	37.497123
H	40.822939	52.203659	37.340191
H	39.016966	40.514612	46.839600
H	36.875341	51.666829	39.332697
H	35.571419	54.361934	39.087310
H	43.209262	40.884903	39.614891
H	47.492965	45.269876	37.728535
H	47.970985	51.802804	40.312763
H	45.418808	51.128058	43.755430
H	49.042993	42.478109	46.500798
H	50.843065	44.779121	40.753959
H	47.262042	38.466526	41.615254
H	50.556020	46.783427	42.439575
H	48.824654	39.896565	40.086323
H	44.027942	39.219733	45.736195
H	47.294428	47.669802	46.562654
H	45.618310	46.145247	47.949529
H	43.647488	41.200178	47.459576

DHT_17 -OH_UP:TS_4β

C	39.446843	40.747560	45.811926
H	38.704096	40.312661	45.126858
H	39.384267	41.840235	45.708377
O	40.746936	40.269463	45.494701
H	41.297975	41.060148	45.329067
C	36.831458	52.738851	39.052310
O	37.884756	53.271341	38.732220
N	35.651948	53.377728	39.142511
H	34.806575	52.869022	39.375095
C	43.120590	40.199842	40.610650
H	43.288873	39.140549	40.393048
H	43.930540	40.567942	41.247503
H	42.165238	40.308250	41.132590
C	47.632861	44.480896	38.507980
H	48.562868	44.638030	39.057640
C	46.471425	44.730925	39.459799
H	45.513491	44.600374	38.944922
H	46.524982	44.030335	40.297856
H	46.512138	45.747929	39.864464
O	47.616104	43.139366	38.031309
H	46.797810	42.701193	38.355458
C	48.164320	51.002008	39.487251
H	47.191480	50.878370	38.986296
C	49.149748	51.558747	38.436355
H	50.148354	51.724638	38.860101
H	49.268955	50.855364	37.603441
H	48.807347	52.510743	38.014453
C	48.629604	49.607859	39.951736
H	48.729145	49.004298	39.038688
H	49.644221	49.672080	40.371264
C	47.726940	48.844462	40.927042
H	46.679350	48.854856	40.600446
H	48.040388	47.796743	40.997709
H	47.767408	49.257535	41.940532
C	45.447749	50.275036	44.319634
H	46.219764	50.462397	45.069711

H	44.491945	50.122800	44.827736	H	43.079589	42.542184	43.104186
H	45.698143	49.374969	43.753510	H	44.615249	43.539238	43.525917
S	49.060829	41.889429	45.274775	C	42.821485	44.630869	42.521093
N	47.507401	41.426174	42.779833	H	41.748885	44.451945	42.311445
C	48.320082	41.437122	41.657438	C	43.543045	44.492045	41.182228
C	46.960461	40.167034	42.859923	H	43.557783	43.441659	40.882262
C	48.271755	40.137979	41.021453	H	44.589398	44.778287	41.301163
C	47.430901	39.370055	41.754464	C	42.859012	45.359900	40.129695
C	46.067877	39.726802	43.828705	H	41.820280	45.020490	39.987485
H	45.715711	38.705582	43.728903	H	43.354358	45.256086	39.156587
C	45.598358	40.468096	44.900179	C	42.867822	46.832933	40.557706
N	45.964423	41.767755	45.188622	H	43.919240	47.156626	40.562228
C	45.245017	42.130381	46.312809	C	42.277667	47.040557	41.988653
C	44.401085	41.030116	46.727452	H	41.210228	46.768180	41.913499
C	44.624250	40.010567	45.865763	C	42.334060	48.537421	42.372437
C	45.324689	43.364025	46.932774	H	43.380591	48.843895	42.482359
H	44.716687	43.510919	47.818114	H	41.871458	48.693156	43.353259
C	46.104697	44.440424	46.511232	C	41.642402	49.458262	41.347761
C	46.147387	45.737483	47.138903	H	41.776011	50.503305	41.650182
C	47.512859	45.696961	45.351991	H	40.562251	49.260236	41.356506
N	46.938593	44.441610	45.419851	C	42.183290	49.232016	39.924034
C	47.012989	46.506624	46.435601	C	43.611569	49.802111	39.813942
C	48.431269	46.110530	44.400382	H	43.584890	50.876944	40.023535
H	48.826630	47.114130	44.496399	H	44.306539	49.353832	40.526485
C	48.898555	45.364128	43.325572	H	44.044439	49.665401	38.814439
C	49.921350	45.810258	42.404575	C	42.087313	47.727243	39.589316
N	48.472087	44.109480	42.986804	H	41.023175	47.459283	39.716164
C	49.190560	43.741454	41.879839	C	42.356957	47.662700	38.074046
C	49.091238	42.509345	41.234765	H	41.955399	46.751307	37.621448
H	49.679825	42.373463	40.333262	H	43.433157	47.679167	37.864450
Fe	47.127408	42.970716	44.052561	C	41.659864	48.940838	37.528126
C	50.088233	44.811501	41.497732	H	40.744088	48.689249	36.985634
O	45.780815	43.550683	43.159353	H	42.283303	49.489603	36.812616
C	42.009004	46.116334	44.352196	C	41.320899	49.813821	38.777489
C	42.888810	46.088073	43.075831	H	40.269381	49.653966	39.026154
C	44.330749	46.508359	43.446077	O	41.423735	51.224736	38.600039
H	44.941434	46.709434	42.560283	H	42.330793	51.418659	38.310972
H	44.321410	47.416423	44.058063	O	40.214051	53.219788	37.208925
H	44.852760	45.733455	44.008585	H	39.300280	53.173331	37.541683
H	42.024344	47.110460	44.814299	H	40.609500	52.445586	37.665616
H	40.964587	45.921027	44.067873	H	39.134213	40.488200	46.854830
C	42.441163	45.077576	45.406784	H	36.763651	51.663549	39.341930
H	41.724316	45.035262	46.233769	H	35.548557	54.390108	39.093667
H	43.412060	45.363198	45.830153	H	43.097628	40.780811	39.661507
C	42.580127	43.672980	44.835461	H	47.594839	45.192069	37.649012
O	42.142077	42.709864	45.471602	H	48.011921	51.789701	40.275731
C	43.240647	43.542574	43.520717	H	45.368976	51.142677	43.630398

H	49.172982	42.462038	46.542417
H	50.809851	44.746890	40.794703
H	47.286463	38.376558	41.669046
H	50.502400	46.728481	42.508555
H	48.812709	39.841643	40.123900
H	44.063623	39.079090	45.801933
H	47.252763	47.553140	46.616712
H	45.594355	46.020772	48.026764
H	43.631499	41.069064	47.499752

TES_17 -OH_DOWN:RC

C	41.069587	36.593980	47.312770
H	41.544695	37.551582	47.558155
H	41.750599	35.786378	47.606997
O	40.783450	36.563718	45.916625
H	41.370001	35.892715	45.481195
C	44.798212	43.995212	43.818273
H	45.263457	44.371247	44.735215
H	43.896201	44.573850	43.605944
H	44.524908	42.946181	43.963215
C	43.315258	47.320952	39.384105
H	42.462927	47.979675	39.185255
C	42.793986	45.901268	39.599748
H	42.285769	45.512791	38.709943
H	43.606379	45.200497	39.838665
H	42.086248	45.885817	40.435492
O	43.929946	47.856198	40.560173
H	44.577247	47.181848	40.894294
C	36.246535	41.743979	37.520457
H	37.152346	41.996839	38.077717
H	35.416768	42.354077	37.883241
H	36.015780	40.688429	37.690799
S	37.809208	46.755747	45.824011
C	41.273832	40.454165	33.722088
H	42.129281	40.152055	33.102280
H	41.327908	41.548617	33.802535
C	41.439950	39.876015	35.145419
H	42.256549	40.461603	35.588390
C	40.199605	40.101226	36.024572
H	39.355523	39.480464	35.697687
H	40.406676	39.847020	37.070705
H	39.878567	41.148971	35.997933
C	41.885706	38.406813	35.175490
H	42.769806	38.243524	34.547047
H	42.145372	38.100854	36.197123
H	41.099854	37.725533	34.828752
N	40.601071	46.002454	45.027352
C	41.357649	46.997546	44.442908
C	41.298921	45.575017	46.133247
C	42.576021	47.182201	45.197599
C	42.532395	46.317068	46.239724
C	40.870058	44.611354	47.038547
H	41.527688	44.418567	47.880136
C	39.689974	43.889840	46.960783
N	38.777018	43.977224	45.923411
C	37.817048	43.010963	46.170991
C	38.116762	42.329929	47.413220
C	39.265973	42.871495	47.891602
C	36.755798	42.725151	45.327718
H	36.070814	41.945763	45.644172
C	36.493409	43.333271	44.102859
C	35.406316	42.982979	43.218260
C	36.624919	44.658938	42.335346
N	37.227437	44.354798	43.540041
C	35.484007	43.797533	42.138676
C	37.050373	45.636904	41.449401
H	36.452703	45.778843	40.555243
C	38.165272	46.453243	41.601956
C	38.596889	47.448460	40.644353
N	39.005427	46.444825	42.687797
C	39.947181	47.418213	42.453166
C	41.032483	47.685291	43.283940
H	41.728838	48.456399	42.974537
Fe	38.973477	45.110736	44.221870
C	39.685219	48.066594	41.182465
O	39.852348	43.987667	43.444431
C	42.985961	40.559446	39.913556
C	41.804317	41.237882	40.660183
C	42.354559	42.173052	41.771102
H	41.560560	42.614183	42.379389
H	43.029746	41.623154	42.435614
H	42.925621	42.996453	41.331080
H	43.665179	40.107319	40.642880
H	42.586755	39.737360	39.303467
C	43.776512	41.498565	38.993366
H	44.544401	40.955824	38.431170
H	44.306497	42.262371	39.580418
C	42.865111	42.217884	38.013321
O	43.244470	42.597014	36.909808
C	41.506345	42.502392	38.488789
H	40.898763	43.096840	37.811042

C	41.008351	42.104674	39.679733
C	39.632619	42.550770	40.100114
H	39.154263	43.096821	39.278196
H	39.722347	43.251879	40.942750
C	38.771599	41.367458	40.558407
H	38.593998	40.683216	39.714524
H	37.791006	41.731932	40.885509
C	39.467323	40.615342	41.697455
H	39.581999	41.324316	42.528323
C	40.868554	40.111795	41.249594
H	40.676371	39.423604	40.408895
C	41.548328	39.278773	42.365046
H	41.850747	39.940815	43.183922
H	42.472319	38.827056	41.983320
C	40.655932	38.157079	42.942449
H	41.185842	37.674188	43.772977
H	40.498388	37.386788	42.171380
C	39.286208	38.689970	43.397821
C	39.435751	39.599821	44.638397
H	38.473672	40.003762	44.963293
H	39.841976	39.011995	45.465726
H	40.106889	40.443264	44.457479
C	38.651769	39.410647	42.181732
H	38.654591	38.676169	41.356492
C	37.178989	39.610401	42.577981
H	36.523981	39.718643	41.706692
H	37.064877	40.521119	43.174913
C	36.847692	38.346661	43.414006
H	36.144597	37.686622	42.895707
H	36.401789	38.603871	44.377852
C	38.189970	37.619380	43.695365
H	38.319664	36.791182	42.975073
O	38.143392	37.109990	45.017539
H	39.003063	36.681206	45.209663
H	40.126474	36.498066	47.896373
H	45.514389	44.079081	42.971191
H	43.977289	47.395697	38.488626
H	36.413337	41.921480	36.435550
H	36.430450	46.547657	45.753162
H	40.340996	40.228795	33.143914
H	40.280882	48.785028	40.788203
H	43.301609	46.055903	46.836265
H	38.128394	47.606263	39.669795
H	43.401030	47.827965	44.902278
H	39.809575	42.555309	48.782420
H	34.837739	43.791798	41.262759
H	34.657921	42.215634	43.406976
H	37.596758	41.453797	47.818234

TES_17 -OH_DOWN:TS_19

C	41.075169	36.627997	47.347591
H	41.561885	37.569187	47.630711
H	41.744436	35.801546	47.615139
O	40.799755	36.656620	45.948839
H	41.380332	35.991195	45.496119
C	44.828470	44.037992	43.902365
H	45.313589	44.396611	44.815519
H	43.929562	44.629876	43.718941
H	44.551857	42.987865	44.037593
C	43.341016	47.570355	39.509667
H	42.556827	48.314297	39.331800
C	42.689403	46.221371	39.775943
H	42.107277	45.873128	38.917711
H	43.446025	45.455282	39.986478
H	42.027637	46.292633	40.644677
O	44.049750	48.056836	40.654026
H	44.653319	47.332343	40.966309
C	36.200277	41.703674	37.486663
H	37.101480	41.943568	38.054772
H	35.371760	42.316880	37.846474
H	35.959967	40.648558	37.645343
S	37.852142	46.709998	45.735036
C	41.289386	40.476754	33.749715
H	42.146909	40.114064	33.166374
H	41.370168	41.572675	33.761122
C	41.412762	39.989588	35.210830
H	42.269045	40.546227	35.620260
C	40.189232	40.361183	36.063940
H	39.308974	39.770454	35.778743
H	40.379290	40.174568	37.127631
H	39.934102	41.421608	35.954697
C	41.754424	38.499127	35.348658
H	42.619724	38.226315	34.732199
H	41.997113	38.251074	36.389579
H	40.917793	37.855180	35.053670
N	40.604886	45.830691	44.938601
C	41.365377	46.838720	44.377512
C	41.277503	45.415536	46.067378
C	42.570061	47.024613	45.152210
C	42.507440	46.160042	46.194245
C	40.819198	44.478661	46.987157

H	41.469583	44.286397	47.834770
C	39.616542	43.790716	46.929773
N	38.696956	43.894082	45.905601
C	37.717873	42.957171	46.163420
C	38.014879	42.270248	47.404700
C	39.178149	42.787679	47.872933
C	36.643979	42.694949	45.325532
H	35.945631	41.928735	45.645525
C	36.392979	43.299129	44.095504
C	35.321194	42.942782	43.193626
C	36.551444	44.617301	42.327415
N	37.133945	44.314944	43.537871
C	35.409411	43.758660	42.115967
C	36.996764	45.589397	41.441133
H	36.387690	45.749842	40.557858
C	38.132746	46.380632	41.578215
C	38.537657	47.412544	40.645825
N	39.010087	46.323754	42.635296
C	39.936627	47.317292	42.414815
C	41.034414	47.557251	43.238626
H	41.730453	48.337264	42.951714
Fe	38.906414	45.034726	44.206107
C	39.638769	48.015800	41.177962
O	39.592538	43.594812	43.456528
C	42.800515	41.466056	40.504317
C	41.412251	41.937648	41.068544
C	41.673141	43.007995	42.134109
H	40.519574	43.488659	42.814413
H	42.234637	42.657470	43.000821
H	42.059530	43.950812	41.743863
H	43.457219	41.180510	41.332114
H	42.619640	40.556295	39.916579
C	43.513484	42.484318	39.611691
H	44.387544	42.036216	39.123967
H	43.894458	43.325010	40.203375
C	42.609375	43.078465	38.543292
O	43.061383	43.678087	37.573686
C	41.166413	43.018959	38.793094
H	40.547028	43.457183	38.014588
C	40.600165	42.562466	39.930592
C	39.119113	42.702345	40.143707
H	38.654374	43.129239	39.247473
H	38.948203	43.406226	40.968753
C	38.479000	41.362475	40.527332
H	38.585460	40.645773	39.698039
H	37.404138	41.503430	40.689337
C	39.134437	40.801451	41.792553
H	38.947947	41.516311	42.602468
C	40.668946	40.647386	41.602457
H	40.785954	39.910689	40.790094
C	41.324472	40.029196	42.860088
H	41.276090	40.746293	43.687334
H	42.389812	39.839219	42.678341
C	40.659397	38.704707	43.301680
H	41.136668	38.345278	44.221237
H	40.842163	37.939464	42.529899
C	39.140703	38.864169	43.493257
C	38.843912	39.752429	44.720985
H	37.768514	39.889141	44.862953
H	39.228507	39.260396	45.619202
H	39.298606	40.741777	44.640817
C	38.564620	39.428632	42.171432
H	38.883715	38.727152	41.379215
C	37.040250	39.256270	42.305488
H	36.543596	39.190418	41.331285
H	36.599335	40.113130	42.826555
C	36.887765	37.954858	43.136871
H	36.419833	37.146369	42.565457
H	36.275464	38.118907	44.025744
C	38.304766	37.553340	43.623246
H	38.735510	36.802380	42.935983
O	38.180784	37.009215	44.925475
H	39.063533	36.690738	45.206412
H	40.128381	36.522922	47.923808
H	45.529066	44.130009	43.044318
H	43.964094	47.559124	38.583413
H	36.380722	41.890140	36.406001
H	36.463910	46.533341	45.706782
H	40.360463	40.238038	33.170009
H	40.205991	48.776331	40.818933
H	43.257193	45.917702	46.822677
H	38.042659	47.600561	39.689017
H	43.386153	47.695319	44.888660
H	39.721558	42.464135	48.760977
H	34.785419	43.743435	41.224530
H	34.566463	42.183966	43.380856
H	37.489938	41.398963	47.807396

TES_17 -OH_DOWN:TS_6β

C	41.167649	36.410171	47.299435
H	41.664071	37.365341	47.510267

H	41.813989	35.599830	47.657326	C	36.410668	43.317175	44.007524
O	40.925393	36.311751	45.897640	C	35.312895	42.991176	43.131582
H	41.521622	35.621700	45.508564	C	36.519670	44.680915	42.258717
C	44.845767	44.098888	43.931098	N	37.148303	44.341948	43.444087
H	45.350733	44.461224	44.832060	C	35.375254	43.827545	42.066513
H	43.953261	44.701781	43.750616	C	36.929082	45.678620	41.386659
H	44.551379	43.057041	44.084124	H	36.310445	45.846018	40.512314
C	43.401007	47.658638	39.640094	C	38.044369	46.491531	41.546410
H	42.628007	48.420835	39.491774	C	38.460395	47.524804	40.621613
C	42.730515	46.334157	39.969281	N	38.901878	46.440852	42.617460
H	42.097876	45.993282	39.146319	C	39.834253	47.434214	42.414669
H	43.470007	45.543141	40.149575	C	40.924766	47.677800	43.243116
H	42.120851	46.436265	40.871925	H	41.610871	48.469194	42.965091
O	44.182295	48.145192	40.736455	Fe	38.868438	45.087978	44.138342
H	44.777436	47.405125	41.027505	C	39.552037	48.128834	41.170452
C	36.295387	41.787149	37.580102	O	39.861256	43.950476	43.316188
H	37.209519	42.042028	38.124548	C	42.628831	40.958509	39.966909
H	35.471590	42.400901	37.950296	C	41.651311	41.490144	41.051401
H	36.063812	40.732767	37.757246	C	42.450231	42.135737	42.214379
S	37.833244	46.726763	45.669897	H	41.796166	42.435815	43.037100
C	41.317852	40.458268	33.763570	H	43.207790	41.442352	42.596631
H	42.165720	40.079538	33.176348	H	42.965808	43.037037	41.873973
H	41.416469	41.552808	33.770320	H	43.315241	40.233634	40.416430
C	41.444116	39.975266	35.225488	H	42.044104	40.411714	39.214391
H	42.331303	40.496860	35.615692	C	43.433065	42.053039	39.255121
C	40.252084	40.406648	36.095095	H	44.014478	41.643181	38.421052
H	39.338780	39.864474	35.817806	H	44.165474	42.506402	39.937921
H	40.443384	40.199317	37.155409	C	42.546600	43.167962	38.725018
H	40.051688	41.479736	35.996278	O	42.915271	43.933340	37.836374
C	41.723566	38.472739	35.371917	C	41.244243	43.343276	39.375292
H	42.556976	38.154349	34.733880	H	40.621321	44.132304	38.963064
H	41.986310	38.226145	36.408297	C	40.792265	42.604987	40.433810
H	40.850555	37.863664	35.110688	C	39.481852	42.901289	40.992284
N	40.536509	45.933154	44.933198	H	38.951381	43.712470	40.485705
C	41.271833	46.953258	44.374528	H	39.683842	43.538957	42.229855
C	41.238528	45.492381	46.033201	C	38.638258	41.701488	41.353272
C	42.491222	47.139147	45.131803	H	38.327981	41.199655	40.417798
C	42.460659	46.251488	46.154576	H	37.718367	42.011888	41.853683
C	40.824237	44.507041	46.916329	C	39.424194	40.719176	42.222737
H	41.477547	44.312368	47.760703	H	39.642857	41.244146	43.160091
C	39.653409	43.768979	46.816488	C	40.756734	40.294798	41.548609
N	38.749856	43.853567	45.780673	H	40.463693	39.745570	40.638096
C	37.768618	42.922342	46.040751	C	41.522490	39.296954	42.455947
C	38.057557	42.238982	47.287075	H	41.890897	39.824743	43.342313
C	39.218875	42.760964	47.756367	H	42.412164	38.920387	41.935527
C	36.680264	42.682383	45.216258	C	40.673561	38.091668	42.919596
H	35.973503	41.925561	45.539396	H	41.263448	37.489707	43.623401

H	40.456684	37.447447	42.053688
C	39.341084	38.531269	43.553640
C	39.574998	39.227102	44.914242
H	38.635447	39.533653	45.379870
H	40.067899	38.529094	45.593121
H	40.210987	40.111125	44.824031
C	38.628108	39.447312	42.528291
H	38.574901	38.874189	41.585098
C	37.185836	39.551779	43.043908
H	36.480168	39.847690	42.260056
H	37.115086	40.302235	43.837540
C	36.902988	38.132141	43.591706
H	36.230259	37.571435	42.934313
H	36.447568	38.162084	44.582136
C	38.270317	37.409359	43.734983
H	38.395699	36.679845	42.914629
O	38.265526	36.742286	44.983309
H	39.147782	36.345029	45.135417
H	40.203662	36.373209	47.857343
H	45.536233	44.167412	43.061966
H	43.971883	47.616304	38.679528
H	36.448571	41.960480	36.492083
H	36.448462	46.525979	45.657801
H	40.381841	40.231622	33.190480
H	40.131562	48.878227	40.808023
H	43.227042	46.001483	46.759819
H	37.976976	47.713837	39.659054
H	43.306041	47.805962	44.854128
H	39.753019	42.452650	48.655456
H	34.727471	43.825342	41.192210
H	34.564588	42.224277	43.317899
H	37.518381	41.381664	47.706395

TES_17 -OH_DOWN:TS_8

C	41.127226	36.684050	47.380976
H	41.609857	37.615678	47.698584
H	41.789999	35.850505	47.641544
O	40.900448	36.757190	45.974571
H	41.414302	36.035783	45.527267
C	44.909516	44.065188	43.921706
H	45.404999	44.461605	44.813629
H	43.997716	44.634086	43.732569
H	44.647267	43.018639	44.099315
C	43.354288	47.408593	39.393989
H	42.524166	48.096277	39.201436
C	42.797523	46.003730	39.592628
H	42.252297	45.650425	38.710246
H	43.598720	45.278889	39.789656
H	42.115423	45.985700	40.448391
O	43.986954	47.914694	40.572560
H	44.616055	47.221657	40.903554
C	36.285614	41.771438	37.537286
H	37.197229	42.030242	38.083106
H	35.458082	42.380505	37.906857
H	36.059870	40.715728	37.713732
S	37.832251	46.737713	45.741720
C	41.418300	40.404281	33.677729
H	42.235083	40.130998	32.995413
H	41.480874	41.493446	33.806857
C	41.672583	39.756876	35.058196
H	42.510888	40.326429	35.479452
C	40.488450	39.929113	36.022036
H	39.625317	39.324425	35.714377
H	40.759386	39.616436	37.038236
H	40.167129	40.975632	36.073202
C	42.127343	38.291562	34.987162
H	42.971951	38.167875	34.298246
H	42.452350	37.937866	35.974170
H	41.326379	37.620231	34.655751
N	40.617489	45.655524	44.865689
C	41.394542	46.648822	44.295971
C	41.267592	45.261179	46.018561
C	42.581041	46.846204	45.094467
C	42.492300	46.007689	46.155424
C	40.793546	44.346160	46.946147
H	41.422883	44.163767	47.810895
C	39.579829	43.681647	46.871829
N	38.677578	43.788317	45.834701
C	37.640454	42.927675	46.122923
C	37.903167	42.255998	47.378262
C	39.092339	42.724510	47.834449
C	36.531458	42.729218	45.315365
H	35.777600	42.034694	45.670539
C	36.314502	43.317830	44.074055
C	35.216556	43.012654	43.192833
C	36.544397	44.568729	42.252562
N	37.126462	44.256200	43.467648
C	35.355273	43.773437	42.080509
C	37.012117	45.506970	41.345233
H	36.402547	45.681825	40.465788

C	38.161287	46.271977	41.488072
C	38.581842	47.311434	40.572989
N	39.043093	46.178037	42.539118
C	39.993846	47.153161	42.328761
C	41.097487	47.363135	43.146923
H	41.813474	48.124158	42.860023
Fe	38.970689	44.783613	44.065455
C	39.699542	47.880691	41.107421
O	39.774640	43.410606	43.283649
C	43.147704	41.002135	40.021252
C	41.993098	41.795095	40.697434
C	42.580519	42.845078	41.670178
H	41.801549	43.347276	42.248967
H	43.258482	42.354494	42.374527
H	43.153167	43.599478	41.125920
H	43.862670	40.679943	40.784478
H	42.733895	40.089203	39.570627
C	43.889782	41.779372	38.926900
H	44.651642	41.159251	38.441991
H	44.416924	42.643245	39.356262
C	42.928056	42.297740	37.872008
O	43.242283	42.438146	36.694711
C	41.599996	42.689052	38.361892
H	40.960797	43.167681	37.624545
C	41.165455	42.512258	39.628473
C	39.818227	43.044466	40.038794
H	39.313088	43.489346	39.173941
H	39.951783	43.838011	40.784670
C	38.947112	41.951274	40.670753
H	38.709910	41.186851	39.909516
H	37.991926	42.373668	40.997593
C	39.655656	41.264671	41.827880
H	39.706826	42.344177	42.717172
C	41.072669	40.773849	41.460207
H	40.869027	39.982521	40.713164
C	41.765174	40.061701	42.647645
H	41.976424	40.786715	43.440974
H	42.734478	39.663422	42.325953
C	40.919321	38.903660	43.219054
H	41.442771	38.446236	44.068573
H	40.821675	38.121407	42.448948
C	39.519082	39.381336	43.635594
C	39.610005	40.272114	44.888672
H	38.620887	40.562198	45.241543
H	40.089206	39.707477	45.695043
H	40.180945	41.185295	44.706819
C	38.876247	40.090901	42.415706
H	38.913956	39.336824	41.602748
C	37.389434	40.225466	42.773152
H	36.754457	40.312634	41.885065
H	37.227268	41.119630	43.372206
C	37.089900	38.944954	43.595745
H	36.417165	38.260665	43.067557
H	36.617181	39.178488	44.554638
C	38.453689	38.264612	43.888806
H	38.627256	37.459388	43.151393
O	38.407121	37.725167	45.195693
H	39.233303	37.217904	45.341390
H	40.168492	36.569472	47.938888
H	45.592552	44.136159	43.046733
H	44.013212	47.461871	38.496494
H	36.439040	41.945715	36.449772
H	36.448059	46.545515	45.704448
H	40.453122	40.206851	33.143159
H	40.270837	48.646034	40.765021
H	43.224120	45.785256	46.811653
H	38.075094	47.535569	39.630497
H	43.397108	47.523792	44.847200
H	39.622579	42.390146	48.726195
H	34.724139	43.760215	41.193762
H	34.470442	42.244650	43.370404
H	37.367987	41.385959	47.767878

DHT_17 -OH_DOWN:RC

C	37.1547780	47.3148670	41.4416360
H	37.5855740	46.8158370	40.5659860
H	37.1717620	48.3962460	41.2642080
O	37.9114000	46.9484570	42.5941880
H	38.3144130	47.7643790	42.9951640
C	44.6272410	43.4141700	37.5622120
H	44.0122870	43.2174080	36.6795730
H	44.6336400	42.5300900	38.2033540
H	44.1973040	44.2564570	38.1128780
C	48.3871700	40.5144770	39.9620330
H	48.4345850	39.6717040	40.6605180
C	47.3790740	41.5262240	40.4950600
H	46.3949560	41.0567250	40.5945100
H	47.6801470	41.9020590	41.4780390
H	47.2698720	42.3892310	39.8251700
O	47.9501260	39.9303210	38.7365000
H	47.7779160	40.6496290	38.0792670

C	43.6946930	42.1196160	48.8438290	O	42.4052790	41.2969120	41.6629770
H	43.6207670	41.2892580	49.5531410	C	45.8816090	46.5808640	42.2411310
H	42.8900590	42.8326680	49.0519600	C	45.1923870	45.2664180	42.7056640
H	43.5799430	41.7469340	47.8230010	C	44.9960080	44.3207090	41.4974000
S	40.6967010	37.5303180	40.9496150	H	44.4003910	43.4379550	41.7453620
C	47.6475180	47.3364640	48.1176640	H	44.4769300	44.8449170	40.6864940
H	48.4002230	48.1057640	47.8950340	H	45.9482850	43.9701830	41.0880760
H	48.0264640	46.4028570	47.6801880	H	45.3066250	47.0475920	41.4328760
C	46.3278060	47.6860270	47.4086710	H	45.8736920	47.2922530	43.0773580
H	45.8885680	48.5909770	47.8507830	C	47.3412340	46.4113810	41.7588290
C	46.6318130	48.0027000	45.9369250	H	47.8249590	47.3826560	41.6118390
H	47.2083490	47.1941960	45.4717190	H	47.3490100	45.9071940	40.7804000
H	45.7156440	48.1391260	45.3523960	C	48.2220010	45.5839180	42.6809380
H	47.2222160	48.9193720	45.8419720	O	49.4084950	45.8192130	42.8524660
C	45.3132020	46.5429870	47.5385440	C	47.5528880	44.3792160	43.3155420
H	45.0455070	46.3463330	48.5823020	H	48.1770340	44.0290330	44.1430950
H	44.3850990	46.7611350	46.9983500	H	47.5492780	43.5803050	42.5625380
H	45.7301250	45.6176220	47.1246500	C	46.1153030	44.6419140	43.8005110
N	42.1563850	39.7957370	39.4703230	H	46.1866000	45.3991780	44.5954960
C	43.3525360	39.3295770	38.9742620	C	45.5232630	43.3837240	44.4477630
C	41.4912890	40.3831700	38.4135950	H	46.2307900	43.0024020	45.1959980
C	43.4516000	39.6509310	37.5701740	H	45.4029010	42.5924110	43.6958340
C	42.3026490	40.2789490	37.2251380	C	44.1821380	43.6910080	45.1133460
C	40.2341540	40.9650200	38.4798220	H	44.3558580	44.3340750	45.9860840
H	39.8333110	41.3498660	37.5467440	H	43.7289520	42.7670660	45.4945940
C	39.4689040	41.1350940	39.6306020	C	43.1961970	44.3811390	44.1594350
N	39.8525630	40.7679890	40.8981660	H	42.8743330	43.6305470	43.4282590
C	38.8622030	41.2043630	41.7458850	C	43.8230650	45.5959010	43.4000180
C	37.8226690	41.8683050	40.9897140	H	44.0676320	46.3408730	44.1763550
C	38.1937900	41.8112400	39.6845550	C	42.7671870	46.2565610	42.4732350
C	38.8621640	41.0316390	43.1264470	H	42.5555490	45.5924920	41.6278760
H	38.0321670	41.4567760	43.6805000	H	43.1800550	47.1770120	42.0401520
C	39.8503110	40.4014090	43.8679170	C	41.4292280	46.6145890	43.1730930
C	39.8727420	40.2965840	45.3084860	H	40.7169390	46.9717730	42.4158210
C	41.7071780	39.3344220	44.4215380	H	41.6034360	47.4500880	43.8679770
N	40.9851590	39.8098800	43.3482450	C	40.8455230	45.4276660	43.9604820
C	41.0104400	39.6434710	45.6480400	C	40.3754960	44.3305290	42.9791080
C	42.9051630	38.6350310	44.3352250	H	40.0192650	43.4477070	43.5084430
H	43.3425460	38.2868930	45.2650090	H	39.5477550	44.7131330	42.3741180
C	43.5979240	38.3445480	43.1724120	H	41.1686910	44.0178220	42.2974370
C	44.8723640	37.6640310	43.1146710	C	41.9784120	44.9401390	44.9073150
N	43.2167440	38.7370950	41.9030020	H	42.3563760	45.8478620	45.4117320
C	44.2441550	38.3549670	41.0570500	C	41.2428760	44.1917060	46.0251020
C	44.3221130	38.6533640	39.7073690	H	41.8747400	43.9713570	46.8923440
H	45.2263510	38.3620070	39.1816450	H	40.8007210	43.2491840	45.6828410
Fe	41.6129730	39.9011590	41.4228330	C	40.1641110	45.2433880	46.3431870
C	45.2604680	37.6680770	41.8151300	H	40.6614950	46.0780370	46.8528150

H	39.3609850	44.9146720	47.0085080
C	39.6587150	45.7533600	44.9717300
H	39.4853970	46.8445840	45.0132150
O	38.4313730	45.0902640	44.6664290
H	38.0648670	45.5581050	43.8929740
H	36.1013226	46.9820561	41.5673316
H	45.6614917	43.6665500	37.2458586
H	49.4267333	40.9281496	39.9174228
H	44.6763794	42.6293707	48.9531430
H	39.9097191	37.1159169	42.0302831
H	47.6208211	47.2085041	49.2281659
H	46.1327014	37.3346015	41.4362184
H	42.1052525	40.7093586	36.3355111
H	45.4351557	37.3017651	43.9768380
H	44.2864056	39.3966016	36.9211523
H	37.6560600	42.2240128	38.8316942
H	41.3937854	39.4707344	46.6524463
H	39.0950119	40.6567868	45.9803210
H	36.9554565	42.3944623	41.4025751

DHT_17 -OH_DOWN:TS_19

C	37.077332	47.685679	41.158243
H	37.515932	47.257021	40.247777
H	36.893917	48.753080	40.985576
O	37.936142	47.456474	42.265949
H	38.471203	48.274630	42.456566
C	44.658355	43.415678	37.489646
H	44.057417	43.214485	36.597957
H	44.662475	42.528351	38.125833
H	44.215044	44.256724	38.032296
C	48.492357	40.260800	39.716521
H	48.565189	39.345620	40.314758
C	47.448461	41.172508	40.342757
H	46.490260	40.648816	40.421684
H	47.766582	41.491569	41.337962
H	47.291836	42.074155	39.736195
O	48.096178	39.801498	38.428007
H	47.894768	40.581076	37.852345
C	43.746184	42.092648	48.816786
H	43.657866	41.255765	49.516655
H	42.948475	42.813112	49.026792
H	43.634915	41.731345	47.791657
S	40.693059	37.756864	40.979116
C	47.764481	47.144196	47.855552
H	48.511739	47.912122	47.611296
H	48.166835	46.194388	47.482020
C	46.460360	47.432598	47.095190
H	46.020164	48.367649	47.467898
C	46.775056	47.632187	45.604305
H	47.246142	46.735333	45.180469
H	45.865538	47.837319	45.026342
H	47.463930	48.470278	45.447833
C	45.442783	46.299393	47.283215
H	45.146785	46.173190	48.330178
H	44.529226	46.477806	46.706074
H	45.869328	45.348677	46.939112
N	42.259633	39.913589	39.712568
C	43.444562	39.416494	39.191446
C	41.568365	40.484974	38.657880
C	43.509558	39.731072	37.786007
C	42.353636	40.361209	37.460404
C	40.305851	41.052589	38.709842
H	39.903470	41.428840	37.774638
C	39.525058	41.176894	39.848029
N	39.898708	40.786918	41.111249
C	38.882444	41.163292	41.957941
C	37.839322	41.822933	41.199726
C	38.231707	41.816443	39.901205
C	38.866477	40.949020	43.327516
H	38.013143	41.322585	43.882176
C	39.871898	40.334540	44.058875
C	39.897102	40.226193	45.497243
C	41.766377	39.324193	44.619425
N	41.025546	39.776924	43.547666
C	41.053748	39.609408	45.841385
C	42.989097	38.676355	44.536874
H	43.423083	38.315937	45.461550
C	43.706840	38.441737	43.375319
C	44.961732	37.726954	43.312349
N	43.361480	38.881205	42.117320
C	44.357439	38.460897	41.262748
C	44.416328	38.728489	39.902958
H	45.290727	38.389512	39.356068
Fe	41.691772	39.935151	41.640784
C	45.350660	37.730112	42.012324
O	42.300553	41.491348	42.147047
C	45.264053	45.346104	41.572875
C	44.444707	44.298613	42.408812
C	44.108126	43.138934	41.471791
H	43.164615	42.218231	41.885229
H	43.603914	43.448025	40.553415

H	44.916487	42.436240	41.262074
H	44.685258	45.682486	40.706305
H	45.413631	46.227848	42.211148
C	46.633833	44.841241	41.084860
H	47.230212	45.655649	40.658213
H	46.484561	44.111463	40.277866
C	47.468833	44.134140	42.142433
O	48.688672	44.128863	42.108758
C	46.714392	43.327652	43.187191
H	47.370407	43.207482	44.056308
H	46.564525	42.322596	42.771497
C	45.355131	43.899775	43.609861
H	45.555954	44.840437	44.147238
C	44.645929	42.964288	44.595882
H	45.347715	42.666287	45.385792
H	44.330921	42.046330	44.081894
C	43.441800	43.669752	45.215355
H	43.804366	44.481821	45.859806
H	42.888983	42.981307	45.865839
C	42.481815	44.236348	44.160161
H	41.951493	43.386382	43.717947
C	43.191904	45.021176	43.011581
H	43.608067	45.929215	43.480263
C	42.146291	45.500452	41.976670
H	41.711879	44.632459	41.467741
H	42.630687	46.103938	41.198218
C	41.012230	46.350293	42.602657
H	40.265459	46.576519	41.831887
H	41.435855	47.314075	42.929530
C	40.354476	45.653748	43.810441
C	39.537854	44.438077	43.313125
H	39.093992	43.887008	44.144317
H	38.721282	44.787020	42.675667
H	40.151086	43.749623	42.731106
C	41.497022	45.239036	44.775743
H	42.098937	46.152200	44.936330
C	40.795433	45.039559	46.125214
H	41.489212	44.947992	46.968191
H	40.136566	44.163506	46.137083
C	39.993780	46.354184	46.165431
H	40.706564	47.166500	46.348570
H	39.240980	46.439034	46.953333
C	39.425994	46.546153	44.738208
H	39.512514	47.609671	44.454058
O	38.057198	46.157268	44.712965
H	37.714772	46.510339	43.870874
H	36.112511	47.180874	41.378044
H	45.693732	43.673811	37.184732
H	49.508821	40.726323	39.741470
H	44.733058	42.589349	48.938105
H	39.914654	37.258495	42.034488
H	47.706053	47.077616	48.971372
H	46.216629	37.375881	41.637610
H	42.148999	40.841518	36.598423
H	45.497463	37.309238	44.166490
H	44.331068	39.477534	37.119989
H	37.703182	42.249430	39.051773
H	41.441448	39.456512	46.846727
H	39.110370	40.575290	46.163167
H	36.967499	42.338455	41.611551

DHT_17 -OH_DOWN:TS_18

C	37.128997	47.029916	41.426115
H	37.494315	46.534311	40.519499
H	37.254793	48.111159	41.298645
O	37.872314	46.531245	42.537981
H	38.288133	47.299877	43.010662
C	44.619919	43.403352	37.493610
H	44.031988	43.168698	36.600549
H	44.618726	42.539600	38.161403
H	44.163081	44.255603	38.006264
C	48.460865	40.407721	39.885507
H	48.535537	39.537845	40.547679
C	47.435407	41.374360	40.457795
H	46.464534	40.879457	40.561501
H	47.736933	41.744691	41.443412
H	47.294730	42.241939	39.801372
O	48.028418	39.866253	38.639150
H	47.829818	40.608556	38.015721
C	43.735348	42.083638	48.822495
H	43.668437	41.258636	49.538606
H	42.923983	42.790732	49.025104
H	43.624821	41.701425	47.804984
S	40.730416	37.651440	41.013076
C	47.652380	47.333932	48.057263
H	48.397849	48.110648	47.836415
H	48.046351	46.402002	47.629531
C	46.333255	47.662534	47.335852
H	45.879054	48.563456	47.771036
C	46.640477	47.975586	45.863724
H	47.226879	47.170635	45.404782

H	45.724999	48.100102	45.274362	C	48.444604	45.625325	42.648203
H	47.222789	48.897538	45.766988	O	49.602083	45.958472	42.850135
C	45.334973	46.505125	47.466055	C	47.864514	44.377886	43.284838
H	45.051896	46.321144	48.507959	H	48.480290	44.106045	44.144863
H	44.413335	46.696834	46.904792	H	47.960315	43.558603	42.559086
H	45.776322	45.580863	47.076346	C	46.398877	44.534665	43.718601
N	42.086915	39.966996	39.614021	H	46.377753	45.339549	44.469503
C	43.273955	39.514801	39.083756	C	45.911345	43.271853	44.433934
C	41.386336	40.534241	38.576258	H	46.640703	43.000321	45.208265
C	43.334075	39.840143	37.676061	H	45.862756	42.429767	43.730596
C	42.162334	40.444173	37.364133	C	44.541963	43.510465	45.059740
C	40.117002	41.088369	38.668771	H	44.634743	44.253244	45.862711
H	39.694265	41.474925	37.746162	H	44.170022	42.590073	45.527509
C	39.349584	41.210408	39.821598	C	43.529707	43.989632	44.015613
N	39.725416	40.794715	41.077774	H	43.330472	43.139230	43.353615
C	38.702174	41.140234	41.925007	C	44.023172	45.187380	43.140407
C	37.671611	41.833495	41.189455	H	44.076537	46.060790	43.813165
C	38.062552	41.859683	39.890138	C	42.946314	45.497540	42.066199
C	38.686230	40.883682	43.293466	H	42.869483	44.644821	41.381936
H	37.829918	41.236876	43.856479	H	43.251753	46.360622	41.459720
C	39.718352	40.299941	44.016805	C	41.544307	45.774812	42.652075
C	39.766264	40.172540	45.452111	H	40.820139	45.906486	41.835756
C	41.670857	39.391889	44.536959	H	41.567094	46.725763	43.208376
N	40.901614	39.832718	43.478015	C	41.096382	44.654393	43.603362
C	40.962278	39.622196	45.773477	C	40.734515	43.374226	42.848924
C	42.912123	38.773888	44.432800	H	40.230064	42.658759	43.483728
H	43.364344	38.421827	45.354018	H	40.157002	43.568947	41.942958
C	43.614047	38.528190	43.263455	H	41.655414	42.462726	42.316093
C	44.877600	37.825097	43.180915	C	42.217190	44.455139	44.654315
N	43.220860	38.945552	42.010946	H	42.429441	45.458850	45.065243
C	44.217255	38.540606	41.143172	C	41.528078	43.718427	45.808482
C	44.260453	38.833081	39.789264	H	42.129592	43.689745	46.722893
H	45.143267	38.525145	39.236556	H	41.265437	42.687082	45.550682
Fe	41.507820	40.002031	41.569555	C	40.279034	44.605764	45.969017
C	45.236139	37.822362	41.873017	H	40.585176	45.517700	46.497123
O	42.276620	41.563329	41.823908	H	39.469281	44.173546	46.562513
C	46.030826	46.393733	42.111234	C	39.835726	45.011277	44.535232
C	45.468735	45.015009	42.561705	H	39.682212	46.102952	44.495030
C	45.462673	44.038795	41.366136	O	38.615985	44.356145	44.190396
H	44.965699	43.093353	41.602146	H	38.212728	44.907086	43.492985
H	44.929524	44.481536	40.517963	H	36.048055	46.799753	41.552073
H	46.471901	43.812230	41.019875	H	45.660410	43.659304	37.200124
H	45.445487	46.792338	41.273850	H	49.487975	40.850578	39.855045
H	45.910909	47.104517	42.939547	H	44.712439	42.602670	48.927958
C	47.520580	46.371059	41.695248	H	39.930708	37.176252	42.061608
H	47.914125	47.385223	41.570246	H	47.616441	47.212786	49.168547
H	47.616750	45.877394	40.716433	H	46.078008	37.446489	41.464725

H	41.920065	40.900029	36.499402
H	45.436738	37.424870	44.028409
H	44.166934	39.617768	37.011817
H	37.527260	42.307616	39.053599
H	41.369465	39.477331	46.773043
H	39.001895	40.533309	46.133937
H	36.818320	42.354919	41.621612

DHT_17 -OH_DOWN:TS_8

C	37.130124	47.333278	41.421551
H	37.567167	46.847298	40.541376
H	37.123805	48.415830	41.250712
O	37.890069	46.976740	42.574413
H	38.331239	47.788840	42.943141
C	44.618116	43.428013	37.386835
H	44.067325	43.207371	36.467323
H	44.595907	42.554903	38.040627
H	44.139771	44.270954	37.893165
C	48.454677	40.295964	39.713845
H	48.518726	39.376961	40.306926
C	47.399480	41.203734	40.328515
H	46.419547	40.718713	40.297110
H	47.633443	41.427580	41.373806
H	47.316368	42.154535	39.786680
O	48.075815	39.843858	38.416642
H	47.876741	40.627127	37.844995
C	43.737209	42.085898	48.721372
H	43.635464	41.246243	49.415826
H	42.931881	42.802422	48.916318
H	43.651637	41.729001	47.691711
S	40.689193	37.736171	40.982404
C	47.649004	47.168438	47.855161
H	48.395706	47.932742	47.598385
H	48.050268	46.215196	47.487885
C	46.340832	47.453519	47.096174
H	45.867947	48.359658	47.499453
C	46.671392	47.726124	45.620706
H	47.222596	46.886210	45.178944
H	45.763418	47.882357	45.026045
H	47.296588	48.619269	45.510838
C	45.348414	46.289577	47.231277
H	45.071055	46.105902	48.275163
H	44.422819	46.481459	46.675449
H	45.788047	45.365642	46.833790
N	42.292445	40.104070	39.809805
C	43.453224	39.572474	39.274041
C	41.593729	40.659173	38.756074
C	43.496362	39.847093	37.856966
C	42.348131	40.494216	37.541963
C	40.326339	41.215626	38.820003
H	39.911445	41.591285	37.889948
C	39.539228	41.294345	39.957056
N	39.920639	40.895434	41.218845
C	38.868037	41.194332	42.054253
C	37.803081	41.817476	41.298186
C	38.216205	41.866520	40.006713
C	38.849941	40.956101	43.419967
H	37.971163	41.271621	43.970799
C	39.883827	40.392392	44.153288
C	39.880666	40.217659	45.583719
C	41.818537	39.464923	44.717393
N	41.084978	39.935623	43.644617
C	41.066256	39.660873	45.931423
C	43.056936	38.846520	44.632354
H	43.489317	38.469523	45.551412
C	43.766430	38.616520	43.464258
C	44.992936	37.856966	43.380484
N	43.410565	39.073506	42.214948
C	44.376091	38.613709	41.343992
C	44.416079	38.866577	39.980028
H	45.267347	38.489669	39.421104
Fe	41.788885	40.246028	41.778605
C	45.355075	37.842811	42.072667
O	42.483755	41.841438	42.103252
C	45.762590	46.179907	41.882391
C	45.102697	44.817077	42.240886
C	44.967726	43.945999	40.976325
H	44.390669	43.036096	41.159787
H	44.443098	44.510078	40.196989
H	45.939888	43.661074	40.563881
H	45.214460	46.668279	41.068874
H	45.687098	46.846426	42.752493
C	47.248642	46.087266	41.470202
H	47.694327	47.082161	41.373732
H	47.328006	45.608140	40.482667
C	48.105736	45.278291	42.427505
O	49.249907	45.589273	42.720693
C	47.468172	44.001554	42.943231
H	48.059398	43.637024	43.789464
H	47.555973	43.256033	42.143877
C	45.989426	44.163054	43.346235

H	45.977564	44.868550	44.193441
C	45.403117	42.843241	43.864125
H	46.085152	42.418170	44.609524
H	45.319525	42.112792	43.051204
C	44.036988	43.067290	44.513929
H	44.186644	43.626673	45.454404
H	43.582119	42.110670	44.797101
C	43.067079	43.866268	43.647965
H	42.655742	42.835860	42.781638
C	43.707670	45.073194	42.898143
H	43.941650	45.774523	43.721865
C	42.669071	45.820954	42.025571
H	42.337671	45.179418	41.202131
H	43.140015	46.697210	41.564431
C	41.444858	46.306276	42.840933
H	40.720998	46.780605	42.163842
H	41.777334	47.084447	43.546808
C	40.781477	45.152904	43.611911
C	40.108235	44.203003	42.606199
H	39.597455	43.397427	43.121970
H	39.360740	44.756766	42.027731
H	40.828861	43.766384	41.914357
C	41.897902	44.450575	44.441461
H	42.406115	45.281654	44.969750
C	41.152402	43.754139	45.586410
H	41.811744	43.388558	46.380623
H	40.537266	42.919069	45.242674
C	40.286703	44.945755	46.041990
H	40.956947	45.680476	46.508096
H	39.509560	44.731836	46.782475
C	39.726307	45.558548	44.737944
H	39.663545	46.658797	44.823044
O	38.421299	45.020484	44.527450
H	38.037176	45.542432	43.798217
H	36.084956	46.978675	41.552988
H	45.665808	43.690981	37.133178
H	49.473973	40.757465	39.746336
H	44.719163	42.584948	48.868093
H	39.912109	37.274128	42.051685
H	47.602139	47.114467	48.971704
H	46.200264	37.456301	41.681756
H	42.122539	40.948019	36.670918
H	45.518130	37.402312	44.222213
H	44.298350	39.556300	37.182104
H	37.676589	42.287671	39.158261
H	41.433907	39.479050	46.939142
H	39.087310	40.550787	46.248060
H	36.925299	42.319796	41.712993

TES_19A1:RC

C	47.953682	45.085459	48.228244
H	47.856833	44.258182	48.948678
C	46.615476	45.283773	47.501709
H	46.652790	46.152011	46.834779
H	46.386215	44.410145	46.889446
H	45.773517	45.425032	48.185389
C	49.068561	44.737991	47.208737
H	49.121251	45.528534	46.447275
H	50.053386	44.729320	47.692230
C	48.872227	43.384080	46.511878
H	49.742590	43.132541	45.895053
H	48.738771	42.577514	47.243235
H	47.999379	43.370455	45.852779
C	45.497777	39.481453	40.344098
H	46.330826	40.148737	40.583392
H	44.582882	40.089700	40.335626
C	45.311374	38.424627	41.436388
O	44.801582	37.341672	41.241932
O	45.677358	38.814897	42.670139
H	46.060301	39.728838	42.670537
C	46.688445	43.496503	37.708031
H	46.535549	44.345064	37.038696
C	46.237428	43.902391	39.100960
H	46.794682	44.781120	39.443874
H	45.168294	44.149859	39.115583
H	46.390990	43.092445	39.828099
O	48.086027	43.220106	37.645841
H	48.334575	42.694536	38.444826
C	41.957690	48.221786	38.683519
H	42.444839	48.883501	37.954814
C	42.000789	48.898113	40.062087
H	41.504672	48.281221	40.823132
H	43.041370	49.036957	40.375986
H	41.517803	49.883289	40.067885
C	42.749854	46.901119	38.700606
H	42.823767	46.436134	37.710216
H	43.774393	47.086319	39.039494
H	42.296291	46.167412	39.380180
C	39.682604	53.163849	45.630870
O	39.249445	54.281711	45.849121

N	39.761235	52.132145	46.515136	C	45.016050	45.195225	42.912685
H	40.415234	51.368654	46.322812	C	45.016976	46.041954	41.614244
S	50.354231	49.565886	39.822572	H	44.008944	46.159481	41.209728
C	36.959963	43.162307	45.471572	H	45.431931	47.033865	41.796533
H	36.156625	42.895650	46.173135	H	45.644541	45.596855	40.848061
H	37.249058	42.235104	44.964571	H	47.081552	45.809561	43.189399
C	38.167591	43.637068	46.313578	H	46.460638	44.773349	44.473760
H	38.164318	42.986145	47.201359	C	47.164997	43.725424	42.732138
C	39.512042	43.388970	45.620999	H	48.106926	43.490572	43.239201
H	39.628465	43.987000	44.713142	H	47.430226	43.977019	41.697455
H	40.346724	43.639612	46.287175	C	46.307353	42.482076	42.708678
H	39.619461	42.334445	45.337719	O	46.806196	41.347750	42.707223
C	38.039499	45.077354	46.831241	C	44.866055	42.680009	42.612034
H	38.886539	45.336742	47.477577	H	44.273779	41.776311	42.471019
H	38.015954	45.807472	46.011959	C	44.268222	43.892765	42.643678
H	37.125395	45.209051	47.420110	C	42.783337	43.998543	42.394510
N	50.218052	46.646226	39.843725	H	42.340218	43.001167	42.305819
C	50.338534	46.088171	38.592770	H	42.625480	44.490643	41.424195
C	51.270122	46.193653	40.599130	C	42.072571	44.819708	43.480989
C	51.557320	45.309947	38.542414	H	42.078636	44.254151	44.423071
C	52.095601	45.320601	39.788824	H	41.019992	44.956466	43.200642
C	51.455204	46.431420	41.955992	C	42.751114	46.177435	43.696868
H	52.302305	45.933941	42.421152	H	42.677693	46.734930	42.754825
C	50.642330	47.213478	42.769486	C	44.248218	45.962868	44.053854
N	49.554706	47.934085	42.357208	H	44.251572	45.297700	44.932885
C	49.062238	48.590717	43.456182	C	44.982516	47.258036	44.483565
C	49.860204	48.246436	44.623699	H	45.200572	47.883884	43.614996
C	50.826777	47.392357	44.194654	H	45.957769	46.995654	44.902176
C	47.986908	49.474830	43.442171	C	44.205163	48.091940	45.515953
H	47.741720	49.980841	44.371384	H	44.738903	49.030412	45.709849
C	47.212447	49.796570	42.330408	H	44.153928	47.553139	46.474352
C	46.160430	50.791485	42.294746	C	42.777665	48.361625	45.025168
C	46.364658	49.796255	40.278684	C	42.800873	49.256356	43.764772
N	47.320255	49.216505	41.084109	H	41.792545	49.511395	43.413523
C	45.641333	50.788632	41.041041	H	43.346142	50.187397	43.951306
C	46.095373	49.444009	38.960858	H	43.311091	48.787299	42.924611
H	45.261718	49.939824	38.471292	C	42.073134	47.007917	44.785903
C	46.784422	48.495043	38.216476	H	42.170695	46.445130	45.729760
C	46.411805	48.061905	36.884321	C	40.583524	47.376619	44.636442
N	47.905150	47.819713	38.622883	H	39.924093	46.547728	44.907222
C	48.273022	46.993626	37.588688	H	40.347968	47.643924	43.598542
C	49.416796	46.208507	37.558737	C	40.401798	48.596674	45.581443
H	49.599181	45.636309	36.655613	H	39.749942	48.356568	46.424090
Fe	48.727509	47.845967	40.495459	H	39.943064	49.441250	45.058729
C	47.325964	47.137299	36.499418	C	41.826957	48.988871	46.065649
O	47.844015	46.528256	40.953911	H	42.028102	48.554262	47.049548
C	46.470419	44.925419	43.387657	O	42.014099	50.400742	46.257225

H	42.203396	50.752565	45.369252
H	48.209079	46.009969	48.809829
H	45.636770	39.048699	39.325686
H	46.070432	42.650410	37.315186
H	40.899111	48.076988	38.344242
H	40.026884	52.832529	44.617934
H	39.619710	52.254172	47.522221
H	49.877088	50.823261	40.193614
H	36.485385	43.825455	44.699193
H	47.272826	46.554487	35.677763
H	53.035048	45.048801	40.037866
H	45.560508	48.430461	36.312448
H	51.879542	44.711358	37.693694
H	51.570008	46.853877	44.781395
H	44.883885	51.467898	40.651669
H	45.838950	51.425899	43.120785
H	49.682905	48.600744	45.645299

TES_19A1:TS_19

C	47.937365	44.924799	48.301899
H	47.932878	44.129257	49.062266
C	46.549284	44.987889	47.650477
H	46.501478	45.775625	46.889357
H	46.323944	44.036449	47.163373
H	45.748094	45.170714	48.369585
C	49.014446	44.595993	47.236598
H	49.011781	45.382651	46.467509
H	50.018074	44.621125	47.677867
C	48.824837	43.231829	46.558144
H	49.684028	42.984588	45.923980
H	48.721199	42.431917	47.299725
H	47.934409	43.195780	45.923609
C	45.572180	39.499630	40.376398
H	46.412021	40.171509	40.581065
H	44.655329	40.105512	40.382871
C	45.408565	38.457463	41.484838
O	44.875453	37.384765	41.319648
O	45.814396	38.857152	42.706061
H	46.268452	39.730267	42.677300
C	46.660686	43.486972	37.761260
H	46.467710	44.353318	37.123104
C	46.227309	43.827433	39.180134
H	46.789736	44.693748	39.539628
H	45.153976	44.052153	39.224754
H	46.411449	42.995037	39.872882
O	48.068668	43.265175	37.673321
H	48.343163	42.692470	38.432074
C	41.926028	48.219972	38.778776
H	42.450859	48.846124	38.044691
C	41.902410	48.955277	40.127863
H	41.351287	48.381071	40.883833
H	42.924895	49.090241	40.499588
H	41.441085	49.947945	40.064961
C	42.700231	46.894669	38.897663
H	42.817295	46.381092	37.936264
H	43.708295	47.088439	39.281695
H	42.203275	46.200913	39.588156
C	39.703206	53.169224	45.619793
O	39.278494	54.288942	45.836211
N	39.773679	52.139041	46.506274
H	40.393979	51.352214	46.313508
S	50.300517	49.489957	39.854086
C	37.026193	43.184597	45.404969
H	36.255322	42.916581	46.140198
H	37.295928	42.257588	44.887447
C	38.266614	43.665660	46.194611
H	38.317263	42.999414	47.068869
C	39.583363	43.455179	45.435277
H	39.653321	44.082194	44.540054
H	40.443053	43.696117	46.073237
H	39.690494	42.411497	45.115535
C	38.139667	45.094063	46.743781
H	38.997122	45.345324	47.380088
H	38.096176	45.837652	45.937434
H	37.234962	45.209374	47.350547
N	50.139715	46.651509	39.868866
C	50.254514	46.080510	38.618504
C	51.222873	46.230287	40.610058
C	51.479708	45.320582	38.559432
C	52.060577	45.393787	39.783631
C	51.420549	46.478916	41.959905
H	52.284714	46.009205	42.419585
C	50.593391	47.240919	42.772774
N	49.491189	47.954061	42.365631
C	49.006201	48.610250	43.471278
C	49.804656	48.260732	44.629906
C	50.776902	47.417362	44.194145
C	47.946822	49.508885	43.452373
H	47.707337	50.026280	44.375121
C	47.190996	49.832000	42.334128
C	46.170027	50.845907	42.280394

C	46.336730	49.834393	40.268567
N	47.283333	49.223266	41.085415
C	45.653706	50.847879	41.024309
C	46.050848	49.485527	38.958113
H	45.227773	49.995289	38.468028
C	46.723449	48.519571	38.225441
C	46.356732	48.078074	36.899091
N	47.832752	47.832155	38.652653
C	48.195716	46.987001	37.624421
C	49.324998	46.187099	37.594370
H	49.491255	45.597819	36.700837
Fe	48.646602	47.877638	40.516529
C	47.257297	47.131823	36.534772
O	47.820613	46.409898	40.978266
C	46.708875	44.942843	43.549201
C	45.309940	45.282981	42.941567
C	45.428195	46.172961	41.689184
H	45.101212	47.205671	41.804764
H	46.700447	46.375052	41.350280
H	45.057936	45.744717	40.760363
H	47.351931	45.824020	43.498855
H	46.572742	44.711778	44.611201
C	47.432400	43.768530	42.879461
H	48.346281	43.508071	43.423102
H	47.735862	44.047589	41.861858
C	46.557549	42.538046	42.787958
O	47.023685	41.397044	42.824232
C	45.130517	42.774313	42.583571
H	44.530819	41.886180	42.391594
C	44.558566	43.997120	42.575897
C	43.099706	44.135890	42.221823
H	42.660494	43.149712	42.038618
H	43.014949	44.696597	41.278922
C	42.321473	44.893074	43.305360
H	42.279630	44.284285	44.219067
H	41.285151	45.037731	42.975976
C	42.980907	46.242149	43.609702
H	42.958836	46.831506	42.681642
C	44.458757	46.029595	44.047285
H	44.420020	45.341158	44.904523
C	45.153027	47.318728	44.549590
H	45.414950	47.965560	43.707016
H	46.105066	47.055885	45.021196
C	44.308192	48.124367	45.554222
H	44.817016	49.066973	45.787431
H	44.227232	47.568365	46.499729
C	42.897345	48.380684	45.008996
C	42.951342	49.315677	43.780663
H	41.954796	49.525749	43.371088
H	43.419981	50.268841	44.041924
H	43.541195	48.904409	42.961695
C	42.236042	47.022273	44.694819
H	42.307689	46.427293	45.621361
C	40.744726	47.361065	44.497125
H	40.094982	46.505994	44.700109
H	40.545525	47.669023	43.462728
C	40.493688	48.534943	45.484613
H	39.824134	48.242247	46.297081
H	40.020589	49.383388	44.980576
C	41.888896	48.957718	46.028417
H	42.069327	48.509147	47.009584
O	42.027453	50.367288	46.256304
H	42.125672	50.759485	45.370711
H	48.181042	45.886616	48.827408
H	45.682546	39.051915	39.361220
H	46.068676	42.635418	37.347228
H	40.886101	48.061450	38.394818
H	40.049040	52.834792	44.607971
H	39.627109	52.255083	47.512675
H	49.850432	50.765654	40.204099
H	36.518278	43.847709	44.654718
H	47.216993	46.551419	35.710704
H	52.992319	45.099140	40.037788
H	45.510335	48.447836	36.322174
H	51.839334	44.774468	37.690065
H	51.523114	46.882665	44.779626
H	44.885796	51.518236	40.641640
H	45.845043	51.477748	43.106608
H	49.633736	48.614732	45.651463

TES_19A1:TS_2β

C	47.964712	44.885093	48.289375
H	47.996610	44.085491	49.046185
C	46.564641	44.904906	47.664020
H	46.470159	45.697127	46.913291
H	46.367558	43.953095	47.164877
H	45.772940	45.045771	48.404193
C	49.038592	44.598599	47.207181
H	49.036078	45.414442	46.470759
H	50.041631	44.606810	47.652793

C	48.849650	43.267591	46.464788	N	50.253469	46.551109	39.869692
H	49.727727	43.035520	45.851674	C	50.386653	46.031122	38.600189
H	48.705349	42.435920	47.165675	C	51.323355	46.111019	40.610561
H	47.989136	43.281819	45.789813	C	51.627744	45.295306	38.528399
C	45.907091	39.786445	40.443111	C	52.190235	45.321620	39.763331
H	46.709743	40.528409	40.411491	C	51.487117	46.302496	41.977124
H	44.969199	40.350003	40.544131	H	52.347977	45.824267	42.435422
C	46.056310	38.951314	41.708117	C	50.642173	47.036165	42.801626
O	45.747382	37.783418	41.812446	N	49.531152	47.732889	42.396016
O	46.486933	39.660703	42.763701	C	49.066832	48.421823	43.493670
H	46.801985	40.573547	42.504044	C	49.883713	48.096619	44.650792
C	46.615243	43.512511	37.715047	C	50.843701	47.235619	44.219485
H	46.404317	44.367802	37.067005	C	48.015747	49.331859	43.481461
C	46.167962	43.856806	39.127221	H	47.784069	49.843606	44.410211
H	46.695802	44.749496	39.475416	C	47.248058	49.669681	42.370493
H	45.087941	44.049567	39.172297	C	46.210749	50.677137	42.336693
H	46.391549	43.045967	39.830484	C	46.405122	49.692583	40.313985
O	48.028181	43.306913	37.627952	N	47.352180	49.092383	41.121543
H	48.345835	42.868666	38.454041	C	45.696681	50.689095	41.080627
C	41.929319	48.161115	38.743766	C	46.136131	49.360588	38.992162
H	42.446236	48.803503	38.017077	H	45.304438	49.863823	38.507833
C	41.953344	48.853941	40.115663	C	46.824340	48.419096	38.239283
H	41.417211	48.261999	40.869045	C	46.454450	47.996406	36.902748
H	42.987180	48.963939	40.461303	N	47.945944	47.745156	38.641547
H	41.501197	49.853499	40.095806	C	48.315149	46.927258	37.604391
C	42.689707	46.822579	38.796117	C	49.462000	46.148382	37.570191
H	42.765911	46.338334	37.815271	H	49.641729	45.578767	36.666067
H	43.712883	46.987027	39.148567	Fe	48.742597	47.707540	40.533201
H	42.210727	46.112295	39.482462	C	47.368997	47.073299	36.515731
C	39.631466	53.218645	45.663990	O	47.740192	46.320464	40.789681
O	39.270237	54.361802	45.881692	C	46.570250	45.451609	43.558097
N	39.627023	52.176474	46.539636	C	45.089904	45.589075	43.109434
H	40.258277	51.394040	46.350435	C	44.964951	46.448209	41.826132
S	50.342372	49.432220	39.915972	H	43.931867	46.490973	41.471625
C	36.914958	43.202215	45.522753	H	45.314719	47.469873	41.988213
H	36.115075	42.928940	46.225226	H	45.578976	46.037736	41.027741
H	37.206375	42.277700	45.012233	H	47.037579	46.439791	43.556827
C	38.124667	43.677405	46.363257	H	46.565737	45.113316	44.609148
H	38.121937	43.026341	47.250746	C	47.399747	44.499437	42.717843
C	39.467214	43.430425	45.666270	H	48.451406	44.408592	42.997738
H	39.583457	44.034922	44.762107	H	47.698247	45.484302	41.528714
H	40.304503	43.674689	46.330894	C	46.766842	43.229413	42.463817
H	39.572048	42.378283	45.374622	O	47.370243	42.170978	42.141744
C	38.001881	45.117444	46.881551	C	45.293473	43.164089	42.564722
H	38.859200	45.377140	47.513964	H	44.865015	42.183542	42.368277
H	37.968042	45.844421	46.060826	C	44.498815	44.218617	42.807952
H	37.097316	45.251528	47.484101	C	42.995701	44.112813	42.674680

H	42.691730	43.060562	42.671109
H	42.710628	44.513771	41.689303
C	42.218238	44.889193	43.747643
H	42.270136	44.351700	44.704500
H	41.156788	44.925759	43.468373
C	42.769146	46.307982	43.932556
H	42.644376	46.831765	42.977632
C	44.280551	46.221168	44.285343
H	44.344516	45.514007	45.127312
C	44.934983	47.531391	44.783364
H	45.185110	48.179962	43.939845
H	45.890769	47.290556	45.257529
C	44.072142	48.311007	45.776908
H	44.531338	49.282497	45.985691
H	44.010712	47.772589	46.734849
C	42.661901	48.491672	45.219521
C	42.701290	49.352956	43.934063
H	41.700245	49.560272	43.536050
H	43.203113	50.310224	44.113693
H	43.262666	48.877288	43.130940
C	42.020653	47.110132	44.988374
H	42.108969	46.567696	45.944354
C	40.525557	47.426274	44.776160
H	39.881213	46.586537	45.048119
H	40.321854	47.649242	43.721124
C	40.269601	48.676006	45.666909
H	39.576241	48.452459	46.479218
H	39.827550	49.492428	45.088527
C	41.663207	49.101858	46.207753
H	41.829295	48.676285	47.201907
O	41.847723	50.512796	46.391189
H	42.077549	50.855065	45.509419
H	48.193535	45.852174	48.814328
H	45.867891	39.220086	39.483025
H	46.049859	42.640792	37.301975
H	40.875885	48.032912	38.381737
H	39.976011	52.868928	44.655389
H	39.519882	52.294723	47.551534
H	49.867670	50.710330	40.230793
H	36.437547	43.864463	44.751229
H	47.326517	46.498218	35.688287
H	53.131375	45.054950	40.015472
H	45.591115	48.355103	36.342541
H	52.013066	44.797170	37.642523
H	51.603183	46.715234	44.801531
H	44.929028	51.365107	40.705187
H	45.882840	51.304255	43.166015
H	49.724858	48.470406	45.670161

TES_19A1:TS_1β

C	47.930409	45.097834	48.187489
H	47.794909	44.267906	48.898286
C	46.614927	45.341261	47.427734
H	46.700194	46.209441	46.763842
H	46.370635	44.479418	46.803940
H	45.759724	45.509569	48.089779
C	49.056164	44.726979	47.188978
H	49.144980	45.521459	46.435150
H	50.030744	44.688241	47.690670
C	48.836496	43.384040	46.477712
H	49.712379	43.112312	45.877496
H	48.665979	42.575610	47.199738
H	47.977993	43.400796	45.799757
C	45.629491	39.568388	40.428476
H	46.473373	40.246357	40.591633
H	44.716773	40.179817	40.455208
C	45.513173	38.557414	41.569902
O	45.068297	37.438307	41.436179
O	45.858426	39.027891	42.781261
H	46.243391	39.939439	42.740383
C	46.681635	43.484418	37.711943
H	46.507623	44.341872	37.057155
C	46.246627	43.857871	39.118694
H	46.827703	44.712465	39.480442
H	45.180625	44.114441	39.148069
H	46.394806	43.025946	39.821756
O	48.083561	43.222972	37.630972
H	48.356195	42.737171	38.447943
C	41.950181	48.241727	38.614414
H	42.415801	48.923801	37.890286
C	42.017810	48.887274	40.006835
H	41.538678	48.252088	40.763597
H	43.062562	49.024901	40.306050
H	41.529457	49.869136	40.042167
C	42.749563	46.926189	38.576439
H	42.805533	46.492488	37.570889
H	43.779363	47.107235	38.898205
H	42.315575	46.168979	39.242212
C	39.689932	53.153035	45.627473
O	39.245915	54.267043	45.845265
N	39.783225	52.126107	46.514384

H	40.436680	51.361220	46.325652	C	44.878812	46.274819	41.396021
S	50.308086	49.495878	39.869235	H	43.821341	46.380642	41.151589
C	36.983262	43.165238	45.449781	H	45.308205	47.267852	41.527831
H	36.192085	42.895388	46.163276	H	45.388016	45.838798	40.541874
H	37.265525	42.240081	44.935486	H	47.313498	46.055858	41.959620
C	38.203872	43.639665	46.274357	H	46.950325	45.661833	43.872029
H	38.218072	42.982301	47.157003	C	47.318309	43.931571	42.610466
C	39.538393	43.403172	45.556488	H	48.197785	43.784607	43.246290
H	39.638456	44.010643	44.652068	H	47.710369	43.951827	41.580766
H	40.383713	43.649786	46.210392	C	46.455213	42.692440	42.702108
H	39.644250	42.352221	45.260655	O	46.958334	41.568930	42.837230
C	38.077672	45.074803	46.805567	C	45.020336	42.865164	42.551686
H	38.930379	45.329613	47.446308	H	44.441830	41.944085	42.490117
H	38.047347	45.809795	45.991785	C	44.402700	44.064383	42.484566
H	37.168071	45.200869	47.402968	C	42.905188	44.101226	42.300135
N	50.194215	46.599259	39.890611	H	42.509212	43.083439	42.219246
C	50.321952	46.051331	38.637936	H	42.661534	44.606241	41.355974
C	51.255358	46.159600	40.639896	C	42.230198	44.856207	43.457048
C	51.548420	45.282381	38.579814	H	42.319307	44.261635	44.377590
C	52.106920	45.324678	39.814562	H	41.157406	44.956755	43.247589
C	51.428924	46.390702	41.999295	C	42.865426	46.235277	43.666540
H	52.285718	45.909173	42.463538	H	42.701414	46.806400	42.745851
C	50.606787	47.159255	42.819529	C	44.400496	46.098224	43.910160
N	49.509694	47.877782	42.420895	H	44.517570	45.407840	44.761691
C	49.041496	48.548317	43.523328	C	45.075150	47.431774	44.319005
C	49.851052	48.202261	44.679384	H	45.203989	48.075658	43.446414
C	50.809542	47.342796	44.240099	H	46.088224	47.237550	44.679233
C	47.983036	49.454235	43.512142	C	44.313407	48.209850	45.406972
H	47.756137	49.975088	44.437879	H	44.810852	49.170731	45.585520
C	47.206416	49.775408	42.401751	H	44.343875	47.657243	46.358242
C	46.174462	50.789477	42.351494	C	42.849739	48.410780	44.999471
C	46.359588	49.762732	40.347776	C	42.760824	49.301639	43.738625
N	47.299205	49.167944	41.165464	H	41.724874	49.491356	43.431905
C	45.657529	50.779865	41.096332	H	43.253329	50.267229	43.897659
C	46.096468	49.418847	39.025504	H	43.265161	48.863678	42.878508
H	45.276859	49.933542	38.531662	C	42.208024	47.020727	44.799218
C	46.779240	48.468845	38.274485	H	42.386803	46.465211	45.735008
C	46.416453	48.060847	36.930154	C	40.694285	47.310211	44.722660
N	47.883953	47.772119	38.686369	H	40.092377	46.453652	45.036347
C	48.254532	46.959559	37.642985	H	40.394581	47.544680	43.693133
C	49.397485	46.172833	37.606632	C	40.494621	48.540916	45.650949
H	49.578393	45.605042	36.700643	H	39.869460	48.301951	46.513826
Fe	48.709959	47.818616	40.566434	H	39.998807	49.360428	45.121141
C	47.322803	47.128184	36.544382	C	41.918479	48.987775	46.085808
O	47.818664	46.323672	40.951194	H	42.176548	48.548545	47.054865
C	46.627403	45.254708	42.915232	O	42.049576	50.404876	46.285604
C	45.109485	45.408953	42.668154	H	42.240299	50.767414	45.402538

H	48.194129	46.011805	48.783792
H	45.705661	39.099847	39.418223
H	46.074418	42.633404	37.316242
H	40.884431	48.099075	38.298557
H	40.032010	52.823595	44.613705
H	39.635303	52.248616	47.520334
H	49.850113	50.766355	40.226943
H	36.496030	43.830299	44.687250
H	47.276243	46.557123	35.714013
H	53.034380	45.024402	40.077214
H	45.588757	48.462718	36.345932
H	51.913369	44.746917	37.706273
H	51.559483	46.804844	44.818595
H	44.898405	51.455162	40.703098
H	45.854626	51.433571	43.170244
H	49.692317	48.566361	45.699514

DHT_19A1:RC

C	47.950359	45.177051	48.174882
H	47.791645	44.334391	48.865806
C	46.659100	45.455347	47.391127
H	46.772551	46.341451	46.757129
H	46.425154	44.617519	46.732369
H	45.787428	45.614418	48.033689
C	49.096896	44.818827	47.194915
H	49.184354	45.611296	46.439917
H	50.065719	44.793718	47.707869
C	48.907235	43.473577	46.481474
H	48.074870	43.491018	45.773376
H	49.804229	43.204215	45.912370
H	48.712072	42.666043	47.197955
C	45.517906	39.426944	40.309788
H	46.282277	40.169208	40.559812
H	44.544105	39.932047	40.370244
C	45.498263	38.294451	41.340161
O	45.224611	37.145349	41.073198
O	45.738574	38.676933	42.606898
H	45.987590	39.630741	42.660292
C	46.688442	43.559791	37.525049
H	46.543469	44.362660	36.797364
C	46.278029	44.083186	38.888251
H	46.436380	43.318522	39.661235
H	46.875598	44.956723	39.165359
H	45.218004	44.364822	38.906047
O	48.078000	43.238410	37.476302
H	48.325788	42.792282	38.322262
C	41.996789	48.272290	38.656835
H	42.489714	48.912818	37.912811
C	41.993367	48.996537	40.011430
H	41.453701	48.415511	40.770608
H	43.020965	49.123802	40.367992
H	41.530758	49.990147	39.962860
C	42.802768	46.962960	38.748594
H	42.908333	46.458135	37.780595
H	43.814910	47.172614	39.109837
H	42.336782	46.253851	39.444910
C	39.681720	53.186606	45.580985
O	39.292441	54.322047	45.794042
N	39.721280	52.160074	46.471885
H	40.328107	51.359079	46.282458
S	50.367946	49.459865	39.863635
C	36.918671	43.230179	45.493631
H	36.116031	42.930952	46.182453
H	37.231517	42.319042	44.971443
C	38.105255	43.719496	46.356088
H	38.103034	43.063307	47.239693
C	39.457402	43.504780	45.668919
H	39.553376	44.107525	44.761758
H	40.285091	43.777455	46.335204
H	39.592407	42.454246	45.383167
C	37.951253	45.155510	46.876383
H	37.946565	45.886198	46.057857
H	37.021271	45.280696	47.441175
H	38.779732	45.418284	47.544238
N	50.324554	46.540324	39.938792
C	50.510761	46.022576	38.680270
C	51.352439	46.079776	40.723380
C	51.721959	45.230377	38.664991
C	52.224196	45.248232	39.922439
C	51.496267	46.315995	42.082541
H	52.335308	45.829456	42.572697
C	50.660367	47.106092	42.863774
N	49.580056	47.820786	42.423135
C	49.071441	48.491798	43.506866
C	49.851669	48.163517	44.689531
C	50.824273	47.304307	44.285935
C	47.996387	49.374600	43.467579
H	47.735732	49.889393	44.387579
C	47.236092	49.680807	42.341974
C	46.194015	50.684488	42.275646
C	46.411307	49.642137	40.279138

N	47.354909	49.076426	41.108617
C	45.687913	50.657683	41.016255
C	46.164655	49.260527	38.962142
H	45.330996	49.738763	38.455344
C	46.891358	48.325469	38.230339
C	46.577268	47.897275	36.876911
N	48.031888	47.697689	38.656961
C	48.480535	46.930586	37.612688
C	49.647026	46.177667	37.606073
H	49.898360	45.648156	36.693458
Fe	48.788425	47.721970	40.551681
C	47.567681	47.047452	36.493123
O	47.884612	46.409323	40.985550
C	46.275534	44.788417	43.518751
C	44.847426	45.078400	42.996805
C	44.955091	45.810925	41.645937
H	43.981069	45.971267	41.179164
H	45.444571	46.778749	41.761621
H	45.579929	45.267430	40.942731
H	46.896085	45.682961	43.418159
H	46.222496	44.553110	44.587222
C	47.017392	43.651415	42.784142
H	47.929237	43.373515	43.320095
H	47.323143	44.026267	41.797054
C	46.170453	42.419490	42.593149
O	46.584479	41.297141	42.876121
C	44.780425	42.659429	42.054225
H	44.222399	41.716772	42.068873
H	44.856149	42.988191	41.011247
C	44.052798	43.736319	42.884831
H	43.971459	43.337510	43.908168
C	42.618753	43.954701	42.389286
H	42.088483	42.995825	42.331900
H	42.626467	44.355515	41.367991
C	41.873062	44.899937	43.333450
H	41.736470	44.389591	44.296774
H	40.868403	45.105690	42.942396
C	42.619432	46.219205	43.585668
H	42.628593	46.787528	42.646590
C	44.082693	45.953392	44.039741
H	44.003725	45.343132	44.957348
C	44.834589	47.245337	44.441855
H	45.070988	47.841309	43.555859
H	45.800642	46.980666	44.878896
C	44.057206	48.111774	45.449141
H	44.605856	49.042748	45.640312
H	43.982418	47.585624	46.412992
C	42.641813	48.402105	44.930076
C	42.716666	49.325982	43.694185
H	41.729681	49.535116	43.265250
H	43.187359	50.285061	43.941391
H	43.325356	48.902186	42.896022
C	41.926076	47.061297	44.652391
H	41.994846	46.488424	45.592884
C	40.441765	47.437664	44.479079
H	39.777900	46.624642	44.784725
H	40.205817	47.656401	43.429844
C	40.260110	48.706604	45.358236
H	39.509014	48.570102	46.138791
H	39.919493	49.528861	44.724533
C	41.663671	48.997618	45.959697
H	41.768390	48.445062	46.899272
O	41.917688	50.365827	46.323654
H	42.273220	50.803041	45.531952
H	48.203271	46.077617	48.792700
H	45.663463	39.073853	39.263496
H	46.044185	42.707398	37.194157
H	40.949513	48.091434	38.303784
H	40.019932	52.837233	44.571134
H	39.569798	52.288639	47.477170
H	49.862227	50.714435	40.202978
H	36.438946	43.899378	44.730154
H	47.637821	46.479386	35.659625
H	53.115471	44.888300	40.226930
H	45.697962	48.217901	36.316765
H	52.133029	44.733466	37.789275
H	51.557272	46.766083	44.884000
H	44.953818	51.352218	40.611147
H	45.872367	51.345499	43.083865
H	49.662025	48.533381	45.702035

DHT_19A1:TS_19

C	47.920394	45.165524	48.214980
H	47.781244	44.332525	48.921817
C	46.611103	45.418372	47.453609
H	46.700945	46.292824	46.800125
H	46.373514	44.564358	46.817655
H	45.752026	45.580351	48.111438
C	49.049328	44.797692	47.218365
H	49.108421	45.572036	46.441785
H	50.030068	44.799685	47.709125

C	48.861363	43.430415	46.548048	N	50.203474	46.519574	39.962896
H	48.004385	43.409920	45.870567	C	50.410622	46.025198	38.699638
H	49.742819	43.160596	45.955297	C	51.235149	46.067773	40.744937
H	48.706446	42.641186	47.293639	C	51.633651	45.247176	38.680726
C	45.601587	39.507225	40.366848	C	52.126562	45.253874	39.941969
H	46.373118	40.254953	40.576914	C	51.368051	46.305569	42.105276
H	44.630299	40.014885	40.444244	H	52.205312	45.821997	42.601848
C	45.613145	38.401909	41.425672	C	50.531508	47.103102	42.879960
O	45.339356	37.244611	41.196627	N	49.455778	47.825816	42.435886
O	45.881955	38.818635	42.675437	C	48.966136	48.524186	43.511941
H	46.136225	39.772415	42.703217	C	49.745806	48.197680	44.694257
C	46.745653	43.571305	37.513550	C	50.702584	47.316948	44.297766
H	46.589414	44.373068	36.787568	C	47.914274	49.435841	43.459658
C	46.363529	44.090241	38.885089	H	47.676291	49.977585	44.370358
H	46.567027	43.326863	39.647066	C	47.153616	49.738898	42.332829
H	46.943689	44.974843	39.162136	C	46.141668	50.772831	42.248100
H	45.295478	44.338206	38.932457	C	46.317544	49.677802	40.275034
O	48.133221	43.242593	37.446044	N	47.246060	49.097246	41.113626
H	48.393751	42.823148	38.301113	C	45.621763	50.724590	40.994618
C	41.980972	48.266034	38.706754	C	46.071587	49.294287	38.957664
H	42.491735	48.890167	37.960945	H	45.244817	49.779698	38.445727
C	41.947581	49.020223	40.044922	C	46.802747	48.360785	38.226335
H	41.393631	48.454154	40.805123	C	46.504342	47.939727	36.868103
H	42.967451	49.157887	40.419054	N	47.939223	47.731658	38.662977
H	41.485267	50.012059	39.966475	C	48.400414	46.969429	37.618773
C	42.781771	46.958245	38.849123	C	49.562077	46.207365	37.617584
H	42.898299	46.423999	37.898373	H	49.822653	45.693495	36.698792
H	43.789372	47.177336	39.217242	Fe	48.686959	47.762382	40.558805
H	42.305363	46.273271	39.562155	C	47.500888	47.093469	36.490005
C	39.694183	53.209637	45.564795	O	47.775305	46.271606	40.976269
O	39.304440	54.344273	45.782073	C	46.484777	44.903078	43.595938
N	39.737624	52.181662	46.452946	C	45.110347	45.261847	42.963660
H	40.335926	51.375112	46.261011	C	45.321108	46.032295	41.649210
S	50.293247	49.412597	39.922601	H	45.039103	45.501030	40.742270
C	36.986082	43.255936	45.434366	H	44.929428	47.048789	41.630977
H	36.210762	42.961421	46.155603	H	46.694859	46.292819	41.332225
H	37.278966	42.341897	44.905697	H	47.135386	45.780711	43.592954
C	38.204832	43.751091	46.246968	H	46.330839	44.619393	44.642710
H	38.254532	43.076807	47.115686	C	47.245284	43.777118	42.860719
C	39.534420	43.577384	45.502484	H	48.139769	43.482480	43.417114
H	39.601306	44.216493	44.616687	H	47.571587	44.184341	41.890950
H	40.381054	43.830050	46.152616	C	46.390127	42.560957	42.629163
H	39.668009	42.540067	45.171660	O	46.753317	41.429777	42.947214
C	38.041770	45.172358	46.804134	C	45.041077	42.829873	42.012485
H	38.010573	45.921173	46.002378	H	44.457227	41.903218	42.007607
H	37.120063	45.270122	47.388779	H	45.177544	43.142828	40.973995
H	38.879218	45.431076	47.462276	C	44.296513	43.929280	42.789035

H	44.149355	43.538177	43.807862
C	42.898385	44.164645	42.212513
H	42.382167	43.204968	42.073259
H	42.973691	44.625843	41.219122
C	42.095878	45.056930	43.155921
H	41.911064	44.504836	44.089042
H	41.111246	45.280108	42.724805
C	42.827600	46.364992	43.489205
H	42.865481	46.965488	42.570023
C	44.281941	46.111862	43.986431
H	44.183890	45.486233	44.891022
C	44.988542	47.413693	44.427030
H	45.228392	48.027716	43.554432
H	45.953472	47.168935	44.879389
C	44.167614	48.242316	45.433255
H	44.686628	49.185333	45.645146
H	44.096288	47.699786	46.388269
C	42.748529	48.501248	44.904042
C	42.801466	49.467408	43.701201
H	41.817966	49.619307	43.241323
H	43.186646	50.449926	43.997150
H	43.471722	49.118692	42.916402
C	42.084928	47.149664	44.567863
H	42.155577	46.546220	45.489327
C	40.590655	47.481205	44.383259
H	39.949685	46.632807	44.638650
H	40.364424	47.739588	43.341063
C	40.349952	48.702823	45.315058
H	39.601894	48.500274	46.085346
H	39.974717	49.535859	44.715305
C	41.736833	49.026507	45.940579
H	41.852279	48.446692	46.862610
O	41.935678	50.390908	46.349676
H	42.312882	50.859150	45.586867
H	48.182183	46.075713	48.814507
H	45.715485	39.130028	39.324449
H	46.092228	42.720777	37.195019
H	40.942271	48.079150	38.332681
H	40.032285	52.864092	44.553918
H	39.584193	52.306697	47.458294
H	49.811025	50.686997	40.226771
H	36.477664	43.923007	44.688015
H	47.579871	46.528239	35.655139
H	53.040506	44.944778	40.235592
H	45.626669	48.255895	36.303778
H	52.064624	44.776767	37.798914
H	51.439160	46.787855	44.899311
H	44.898559	51.422884	40.576755
H	45.816744	51.439886	43.050395
H	49.567186	48.578024	45.703918

DHT_19A1:TS_2β

C	47.943152	45.189994	48.186557
H	47.783188	44.351957	48.881195
C	46.648124	45.475296	47.412826
H	46.754989	46.370940	46.790131
H	46.412671	44.644450	46.745534
H	45.780448	45.624794	48.062193
C	49.080113	44.816397	47.201623
H	49.173735	45.609570	46.446897
H	50.049903	44.780385	47.710778
C	48.872747	43.474711	46.484760
H	48.063036	43.511552	45.750662
H	49.778052	43.175350	45.944245
H	48.634411	42.676080	47.197015
C	45.866793	39.726009	40.357794
H	46.652667	40.486496	40.358214
H	44.910682	40.259403	40.450512
C	46.005448	38.861301	41.605206
O	45.708389	37.694304	41.689012
O	46.416080	39.560359	42.690097
H	46.750150	40.448341	42.425915
C	46.647010	43.515272	37.555986
H	46.442129	44.337949	36.864839
C	46.198818	43.924378	38.946163
H	46.434153	43.136195	39.669867
H	46.718807	44.830704	39.267541
H	45.117446	44.110614	38.979933
O	48.061974	43.305325	37.495840
H	48.356297	42.818305	38.302802
C	41.962000	48.225523	38.684506
H	42.469022	48.869244	37.951960
C	41.953211	48.934843	40.047727
H	41.394189	48.355997	40.793718
H	42.977554	49.040548	40.423288
H	41.508179	49.936134	40.003478
C	42.744910	46.901050	38.765621
H	42.852622	46.409305	37.791416
H	43.756001	47.083705	39.145485
H	42.260324	46.188426	39.444782
C	39.616924	53.248629	45.617334

O	39.324304	54.409881	45.834416	C	46.410349	45.367097	43.564127
N	39.558416	52.211158	46.498051	C	44.925476	45.465335	43.139297
H	40.113689	51.381174	46.298276	C	44.828510	46.179446	41.775108
S	50.333597	49.357237	39.918422	H	43.802725	46.214749	41.404756
C	36.882376	43.242991	45.536655	H	45.195656	47.206797	41.840084
H	36.073965	42.941245	46.216916	H	45.436566	45.690439	41.015401
H	37.200447	42.332403	45.016457	H	46.870783	46.360658	43.563773
C	38.059411	43.731531	46.412420	H	46.449861	45.028281	44.612534
H	38.049397	43.072648	47.293530	C	47.244475	44.433207	42.726580
C	39.420607	43.519589	45.742035	H	48.290546	44.309654	43.013650
H	39.531466	44.128547	44.840131	H	47.637818	45.444729	41.542538
H	40.238871	43.785092	46.422957	C	46.626615	43.189975	42.285990
H	39.557301	42.471002	45.450722	O	47.301589	42.185316	42.002245
C	37.897288	45.165231	46.936798	C	45.114636	43.094869	42.175346
H	37.872842	45.897595	46.120031	H	44.851313	42.051291	42.378921
H	36.973847	45.279612	47.513277	H	44.850108	43.279386	41.127361
H	38.730269	45.436063	47.596104	C	44.323888	44.032909	43.085461
N	50.294503	46.460348	39.918852	H	44.399730	43.642386	44.107051
C	50.469175	45.954584	38.647628	C	42.830142	44.030027	42.735971
C	51.367402	46.027335	40.666075	H	42.443663	43.003673	42.783915
C	51.713309	45.228035	38.586839	H	42.684199	44.360629	41.698805
C	52.255797	45.255952	39.829339	C	42.034333	44.920223	43.694421
C	51.509803	46.233842	42.031126	H	42.009166	44.443922	44.684933
H	52.372426	45.774025	42.503887	H	40.993507	44.990066	43.357217
C	50.650927	46.971431	42.839259	C	42.630222	46.328161	43.844711
N	49.533107	47.650721	42.411809	H	42.531804	46.840158	42.879574
C	49.064154	48.350223	43.505719	C	44.132842	46.231852	44.234788
C	49.878925	48.051522	44.666585	H	44.160252	45.600705	45.139953
C	50.846343	47.191567	44.249971	C	44.793640	47.570259	44.637599
C	48.010506	49.258134	43.474499	H	45.003588	48.175062	43.750029
H	47.770120	49.773061	44.398752	H	45.765224	47.364129	45.094563
C	47.260359	49.603424	42.354919	C	43.952982	48.385792	45.623620
C	46.243545	50.628132	42.293527	H	44.415532	49.364239	45.791639
C	46.436411	49.594382	40.292714	H	43.918486	47.877788	46.598573
N	47.377830	48.999542	41.115005	C	42.530232	48.544380	45.088985
C	45.740189	50.617889	41.033377	C	42.551762	49.401782	43.802754
C	46.172146	49.225526	38.976752	H	41.552768	49.543675	43.377272
H	45.338658	49.716227	38.483733	H	42.979805	50.392924	43.993747
C	46.870122	48.282893	38.233969	H	43.162155	48.956912	43.017212
C	46.534943	47.860781	36.886959	C	41.894320	47.156108	44.886403
N	48.004678	47.636892	38.659270	H	42.005848	46.630652	45.848696
C	48.414088	46.850521	37.613125	C	40.389856	47.445842	44.704224
C	49.569419	46.083237	37.598977	H	39.769181	46.625266	45.071352
H	49.787030	45.534901	36.690816	H	40.137238	47.577783	43.644244
Fe	48.748271	47.589663	40.566559	C	40.147425	48.766229	45.493128
C	47.495580	46.980692	36.503004	H	39.389094	48.652809	46.267891
O	47.709941	46.217969	40.786026	H	39.785690	49.529411	44.799427

C	41.534884	49.136146	46.089273
H	41.651133	48.618254	47.046991
O	41.779556	50.511466	46.406756
H	42.031428	50.955584	45.580249
H	48.205978	46.091919	48.798046
H	45.865250	39.183826	39.384950
H	46.066165	42.635483	37.188245
H	40.916560	48.065036	38.318178
H	39.946784	52.878749	44.609658
H	39.456263	52.333487	47.510482
H	49.842874	50.631631	40.219467
H	36.409433	43.912389	44.768831
H	47.551214	46.419625	35.664001
H	53.179696	44.954102	40.103207
H	45.661221	48.201173	36.331505
H	52.117000	44.736714	37.704680
H	51.604061	46.676430	44.836986
H	44.983705	51.298440	40.646613
H	45.910006	51.279068	43.105430
H	49.710396	48.433483	45.680390

DHT_19A1:TS_1β

C	47.904910	45.166553	48.106506
H	47.692064	44.321619	48.777896
C	46.650007	45.503944	47.279100
H	46.808360	46.411624	46.683297
H	46.416137	44.696353	46.580679
H	45.758888	45.663870	47.894256
C	49.067723	44.778909	47.158857
H	49.200003	45.575894	46.414153
H	50.020152	44.720139	47.697140
C	48.858362	43.443923	46.430444
H	48.033731	43.481202	45.711946
H	49.757859	43.156228	45.873921
H	48.635863	42.636104	47.137552
C	45.575551	39.464113	40.341386
H	46.345706	40.214059	40.551670
H	44.600421	39.965692	40.410997
C	45.585445	38.359713	41.403131
O	45.310625	37.205488	41.177619
O	45.853429	38.779699	42.657066
H	46.104913	39.727625	42.680747
C	46.688181	43.527426	37.461018
H	46.540690	44.308352	36.710763
C	46.252407	44.069290	38.805849
H	46.432947	43.324777	39.591213
H	46.819859	44.964307	39.070797
H	45.183099	44.311906	38.809139
O	48.084661	43.233063	37.441196
H	48.317987	42.744816	38.267906
C	41.984649	48.290413	38.575711
H	42.448835	48.967615	37.846243
C	42.014284	48.956167	39.959172
H	41.504454	48.338825	40.709731
H	43.050262	49.083414	40.292879
H	41.537606	49.943683	39.964105
C	42.797165	46.982115	38.576357
H	42.900856	46.546526	37.575414
H	43.809955	47.169314	38.947929
H	42.337607	46.223159	39.221613
C	39.696490	53.207376	45.589328
O	39.320665	54.344225	45.808085
N	39.724059	52.173358	46.474397
H	40.316382	51.367320	46.277601
S	50.306343	49.355066	39.954670
C	36.942796	43.241970	45.479419
H	36.150170	42.946563	46.180453
H	37.245579	42.328268	44.955840
C	38.142359	43.731362	46.323588
H	38.156159	43.072162	47.204426
C	39.487747	43.520366	45.619180
H	39.576793	44.129068	44.714092
H	40.322036	43.786367	46.280552
H	39.617544	42.471425	45.325529
C	37.989780	45.164588	46.852138
H	37.972853	45.899394	46.037042
H	37.064035	45.283142	47.425032
H	38.822473	45.427061	47.515423
N	50.223689	46.481193	40.057033
C	50.422222	45.973958	38.793201
C	51.275210	46.039441	40.825438
C	51.647888	45.208759	38.762901
C	52.161384	45.235660	40.015017
C	51.423164	46.287808	42.179820
H	52.273675	45.819595	42.666426
C	50.589126	47.078900	42.961880
N	49.495492	47.790421	42.528395
C	49.025164	48.493639	43.616107
C	49.820335	48.170523	44.781070
C	50.777896	47.297338	44.372483
C	47.981582	49.410521	43.570581

H	47.744857	49.949991	44.481808
C	47.229867	49.718802	42.442677
C	46.215907	50.741378	42.352586
C	46.395059	49.651047	40.380514
N	47.332545	49.071458	41.222415
C	45.706476	50.698091	41.095786
C	46.135857	49.265702	39.069807
H	45.317870	49.768162	38.563345
C	46.837527	48.314976	38.337542
C	46.544942	47.925440	36.970074
N	47.951477	47.649840	38.786526
C	48.406141	46.900518	37.725601
C	49.564896	46.139349	37.717593
H	49.825243	45.625411	36.799715
Fe	48.693520	47.684873	40.700595
C	47.525183	47.063231	36.589837
O	47.770358	46.254012	41.099073
C	46.449700	45.091091	42.969397
C	44.945087	45.280817	42.723820
C	44.761054	45.979863	41.355967
H	43.717614	46.014721	41.047906
H	45.136596	47.003600	41.400640
H	45.331164	45.481800	40.577405
H	47.210798	45.994315	42.042434
H	46.814138	45.474949	43.922713
C	47.148773	43.805582	42.559883
H	48.010458	43.598261	43.199913
H	47.543252	43.918638	41.535279
C	46.267693	42.563217	42.505434
O	46.675551	41.483822	42.906597
C	44.888388	42.777175	41.949187
H	44.326196	41.841383	42.021082
H	44.941466	43.062352	40.894582
C	44.206607	43.883354	42.767214
H	44.245246	43.558577	43.816068
C	42.719422	44.006218	42.417403
H	42.249410	43.017959	42.466220
H	42.590423	44.346198	41.382973
C	42.027946	44.948506	43.399536
H	41.986293	44.457958	44.382304
H	40.989073	45.112098	43.091136
C	42.740538	46.299291	43.557258
H	42.628365	46.854503	42.617743
C	44.261680	46.121587	43.862410
H	44.320075	45.509148	44.777923
C	44.960157	47.461020	44.194920
H	45.072387	48.065286	43.290491

H	45.978490	47.268430	44.547131
C	44.217861	48.282430	45.270669
H	44.732366	49.237884	45.429407
H	44.248926	47.746989	46.230990
C	42.752370	48.500834	44.865564
C	42.684226	49.405785	43.616461
H	41.657792	49.554601	43.264440
H	43.116438	50.392020	43.820078
H	43.246020	49.001447	42.775128
C	42.093729	47.120807	44.665441
H	42.264401	46.570344	45.605500
C	40.580530	47.409230	44.590015
H	39.985981	46.572093	44.964026
H	40.260183	47.580891	43.554208
C	40.391370	48.694918	45.441175
H	39.694596	48.551444	46.268632
H	39.975473	49.481122	44.803117
C	41.813325	49.062964	45.953211
H	42.002491	48.523780	46.887841
O	42.014127	50.440762	46.294823
H	42.225362	50.910980	45.471162
H	48.163471	46.050623	48.748227
H	45.696837	39.083129	39.301070
H	46.050131	42.666093	37.149566
H	40.928499	48.119496	38.249196
H	40.031979	52.860376	44.577621
H	39.577736	52.294233	47.480919
H	49.833887	50.638009	40.245556
H	36.453147	43.910666	44.721837
H	47.616363	46.523655	35.738827
H	53.067100	44.903045	40.307511
H	45.703542	48.297943	36.385179
H	52.068613	44.738452	37.876327
H	51.529270	46.777844	44.962834
H	44.958871	51.374858	40.686146
H	45.890488	51.406475	43.153421
H	49.647773	48.549922	45.789731
